# SALL4‐related gene signature defines a specific stromal subset of pancreatic ductal adenocarcinoma with poor prognostic features

**DOI:** 10.1002/1878-0261.13370

**Published:** 2023-01-21

**Authors:** Angélique Vienot, Franck Monnien, Caroline Truntzer, Virginie Mougey, Adeline Bouard, Jean‐René Pallandre, Chloé Molimard, Romain Loyon, Kamal Asgarov, Gerlinde Averous, François Ghiringhelli, Frédéric Bibeau, Paul Peixoto, Christophe Borg

**Affiliations:** ^1^ INSERM, EFS BFC, UMR1098, RIGHT, University of Bourgogne Franche‐Comté, Interactions Greffon‐Hôte‐Tumeur/Ingénierie Cellulaire et Génique Besançon France; ^2^ Department of Medical Oncology University Hospital of Besançon France; ^3^ Clinical Investigational Center, CIC‐1431 Besançon France; ^4^ ITAC Platform University of Bourgogne Franche‐Comté Besançon France; ^5^ Department of Pathology University Hospital of Besançon France; ^6^ Platform of Transfer in Cancer Biology Georges François Leclerc Cancer Center, Center–Unicancer Dijon France; ^7^ UMR INSERM 1231 Dijon France; ^8^ Department of Pathology University Hospital of Strasbourg France; ^9^ EPIgenetics and GENe EXPression Technical Platform (EPIGENExp) University of Bourgogne Franche‐Comté Besançon France

**Keywords:** fibroblast, pancreatic cancer, SALL4, TGF‐β

## Abstract

Pancreatic ductal adenocarcinoma (PDAC) is marked by molecular heterogeneity and poor prognosis. Among the stemness‐related transcription factors, Spalt‐like Transcription Factor 4 (SALL4) is correlated with unfavorable outcomes; however, its roles in PDAC remain unclear. *SALL4* ^high^ expression defines a PDAC subpopulation characterized by a shortened patient survival. Although *SALL4* expression was mostly evaluated in tumor cells, our findings identify this embryonic transcription factor as a new biomarker in PDAC‐derived stroma. Gene expression analysis reveals that the *SALL4* ^high^ PDAC subset is enriched in cancer stem cell properties and stromal enrichment pathways; notably, an interaction with cancer‐associated fibroblasts (CAF) activated by TGF‐β. A particular oncogenic network was unraveled where Netrin‐1 and TGF‐β1 collaborate to induce *SALL4* expression in CAF and drive their cancer‐stemness‐promoting functions. A 7‐gene stromal signature related to *SALL4* ^high^ PDAC samples was highlighted and validated by immunochemistry for prognosis and clinical application. This *SALL4*‐related stroma discriminated pancreatic preinvasive from invasive lesions and was enriched in short‐term survivors. Our results show that *SALL4* transcriptional activity controls a molecular network defined by a specific stromal signature that characterizes PDAC invasiveness and worse clinical outcomes.

## Graphical abstract

Our findings identify the transcription factor *SALL4* as a new biomarker in pancreatic cancer (PDAC)‐derived stroma. *SALL4* ^high^ PDAC subset is associated with poor prognosis. TGF‐β1 in collaboration with Netrin‐1 acts on fibroblast to promote SALL4 expression. *SALL4* transcriptional activity controls a molecular network defined by a specific stromal signature that characterizes PDAC invasiveness and worse clinical outcomes.

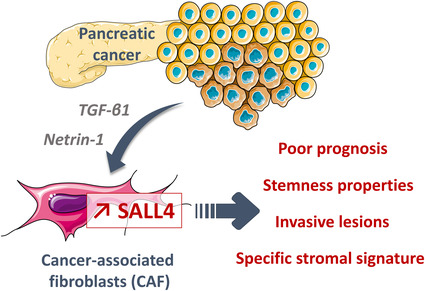

**Abbreviations**
CAF: cancer‐associated fibroblasts
CI: confidence intervals
CSC: cancer stem cells
ECM: extracellular matrix
EMT: epithelial‐to‐mesenchymal transition
FAP: fibroblast activation protein
FFPE: formalin‐fixed and paraffin‐embedded
ICGC: International Cancer Genome Consortium
MMP: metalloproteinases
mRNA: messenger RNA
OS: overall survival
PanIN: pancreatic intraepithelial neoplasia
PDAC: pancreatic ductal adenocarcinoma
RFS: relapse‐free survival
SALL4: Spalt‐like Transcription Factor 4
scRNA‐seq: single‐cell RNA‐sequencing
TCGA: The Cancer Genome Atlas
TGF‐β: transforming growth factor β
αSMA: alpha‐smooth muscle actin

## 1 Introduction

Pancreatic ductal adenocarcinoma (PDAC) has the poorest prognosis among digestive malignancies and is expected to become the second leading cause of cancer death in 2030 [1]. Characterization of recurrent genetic alterations and gene expression studies have identified few molecular or genomic elements allowing prognostic risk stratification or the development of targeted therapies [2, 3, 4, 5]. Moreover, current pathological or molecular classifications are discordant and not applied in routine clinical practice. Indeed, PDAC is marked by a high level of molecular and cellular heterogeneity. Cancer stem cells (CSC) may sustain the occurrence of tumor heterogeneity and have the unique ability to self‐renew and differentiate into heterogeneous tumor cell lineages [6]. The stem‐like characteristics of CSC are due to the dysregulation of stemness signaling pathways, such as Notch, Hedgehog, Wnt, transforming growth factor β (TGF‐β), and pluripotent transcription factor (e.g., NANOG, SOX2, OCT4, KLF4) pathways [7]. However, none of these molecules has yet been introduced in clinical practice as a potential therapeutic target or to predict the risk of death. Notwithstanding, CSC possesses a high tumorigenic ability and similar characteristics to cells that have undergone epithelial‐to‐mesenchymal transition (EMT), allowing CSC to contribute to the metastatic properties of invasive cancers [8].

Spalt‐like Transcription Factor 4 (SALL4) plays an essential role in maintaining the pluripotency and self‐renewal functions of embryonic and hematopoietic stem cells, through interactions with SOX2, OCT4, KLF4, and MYC [9, 10, 11, 12]. SALL4 is a member of the Spalt‐like (SALL) gene family, encodes for a C2H2 zinc finger transcription factor, and enables the transcription of two isoforms according to alternative splicing (SALL4A and SALL4B). Of note, both SALL4 isoforms are co‐expressed when SALL4 is transcriptionally upregulated [13]. During natural development, its expression gradually decreases with tissue differentiation. SALL4 is an oncogenic protein reported as re‐expressed in various solid cancers such as breast cancers, endometrial cancers, lung cancers, esophageal squamous cell carcinoma, liver cancers, and glioma [13, 14, 15]. The functional roles of SALL4 during oncogenesis are still controversial. SALL4 is an unfavorable predictor of survival expectancy, drug resistance, and metastasis in many cancer subsets [15, 16]. SALL4 activates Wnt/β‐catenin and TGF‐β/SMAD signaling pathways, uncovering a mechanism underlying EMT in esophageal squamous cell carcinoma and gastric cancer [17, 18]. A SALL4 expression is highlighted in PDAC cell lines [19], but the role of this protein in human PDAC and the molecular network leading to SALL4 upregulation remain unclear.

In this study, we assessed the prognostic value of SALL4 in human PDAC. In order to characterize the SALL4 overexpressing tumors, we aimed to identify, using *in silico* analyses, the molecular pathways related to SALL4 prognostic value. We established that SALL4 correlated with PDAC stemness properties and the presence of cancer‐associated fibroblasts (CAF) activated by TGF‐β. We next unraveled that SALL4 oncogenic functions in PDAC were potentially mediated by its expression in CAF. The control of SALL4 expression in fibroblasts by TGF‐β1 and Netrin‐1 was reconstituted *in vitro*. Then, we investigated a SALL4‐related stromal signature and validated specific markers by immunohistochemistry in patient samples for a clinical application. Finally, our results identified a molecular network controlling SALL4 expression in PDAC‐associated CAF and correlated with cancer stemness properties and worse clinical outcomes.

## 2 Materials and methods

### 2.1 Patients

Patients with histologically confirmed PDAC, who underwent complete surgical resection at the University Hospital of Besançon between January 2000 and December 2017, were included in a prospective cohort. The database was registered and declared to the National French Commission for bioinformatics data and patient liberty (CNIL; No. of CNIL declaration: 1906173 v 0). The study methodologies conformed to the standards set by the Declaration of Helsinki. A general informed consent was signed by all patients with cancer at the time of their first visit to the Department of Medical Oncology. The experiments were undertaken with the understanding and written consent of each subject. Samples were provided by the regional tumor bank of Franche‐Comté (University Hospital of Besançon, France; registration number BB‐0033‐00024). The project was approved by the scientific board of the biobank (#F1860‐PAC‐MA).

#### 2.1.1 Sample selection and tissue microarray manufacturing

All samples were provided by the regional tumor bank of the Franche‐Comté. For each patient, the most appropriate Formalin‐Fixed and Paraffin‐Embedded (FFPE) sample was chosen from the corresponding hematoxylin–eosin slide. A tissue microarray was constructed from 1 mm tissue cores obtained from FFPE tumor specimens. To evaluate tumor heterogeneity, three spots (1 mm diameter) were punched out per patient by selecting stromal areas including cancer cells, representative of the entire block, avoiding muscle, normal pancreatic tissue, and blood vessels.

Thirty‐four PDAC samples from short‐term survivors (relapse‐free survival [RFS] < 5 months, *n* = 17) and long‐term survivors (RFS > 80 months, *n* = 17) were selected for immunohistochemistry evaluation. For 11 patients from each category, an RNA analysis was performed with the Nanostring assay. Fourteen patients with both pancreatic intraepithelial neoplasia (PanIN) and PDAC were selected to perform PTK7 and SERPINH1 immunohistochemistry on whole slides.

#### 2.1.2 Immunohistochemistry

For each sample, serial 4‐μm‐thick tissue sections were cut from FFPE blocks and placed on positively charged slides. Immunostaining was performed on the Ventana Benchmark ULTRA automated slide stainer (Ventana Medical Systems, Tucson, AZ, USA). Sections were deparaffinized with xylene and rehydrated with graded series of alcohol. Antigen retrieval was carried out by incubating slides for 60 min in CC1 buffer (pH = 8.4). Sections were then incubated for 32 min with the primary antibodies targeting PTK7, SERPINH1, LRRC15, SMA, FAP, and CD3 (Table S1). The chromogenic stainings were visualized using the Ventana Ultraview DAB Detection System (Ventana Medical Systems, Tucson, AZ, USA). The slides were counterstained with hematoxylin.

#### 2.1.3 Pathological analyses

Each slide was digitized after manual optimization with NanoZoomer 2.0 HT digital slide scanner (Hamamatsu®, Hamamatsu City, Japan) to generate a whole slide image (WSI) file in ndpi format. Analysis of the immunohistochemical staining was interpreted with qupath software (v0.2.3) [20] by automatic surface detection expressed in percentage of tissue stained for SERPINH1, LRRC15, αSMA, and FAP antibodies. Positive cells were taken into account to evaluate the cell densities (cells·mm^−2^) of CD3+. Due to the expression of PTK7 antibody in both tumor and stroma cells, we used a semi‐quantitative visual scale to quantify PTK7 in the stroma (0, 1+, 2+, 3+). In case of discrepancies, two pathologists reviewed the slides together and reached a consensus. The mean of the spots for each patient was calculated.

#### 2.1.4 Nanostring

RNA was isolated from FFPE tumor samples of 22 PDAC patients. A morphological control on the corresponding HES slides was carried out to delimit the stromal cell‐rich tumor area. Macrodissections were then realized to maximize the ratio of the stromal cells. Immediately, FFPE slides were deparaffinized. Briefly, 1 mL of xylene was applied for 5 min at room temperature and then centrifuged at 20 000 ***g*** for 5 min. The xylene was removed and 1 mL of absolute ethanol was applied. A new centrifugation step was performed to remove ethanol before total RNA extraction using the RNeasy Mini Kit (Qiagen, Hilden, Germany) according to the manufacturer's instructions. The RNA purity and quantity were assessed on the 2100 Bioanalyzer Instrument (Agilent, Santa Clara, CA, USA). For the Nanostring assay, 100 ng of RNA was used for gene expression profiling using nCounter Metabolic Pathways panel and nCounter Fibrosis panel along with a custom CodeSet according to the manufacturer's instructions (NanoString Technologies, Seattle, WA, USA). Counts of the reporter probes were tabulated for each sample by the nCounter Digital Analyzer and raw data output was imported into nsolver version 4.0 (http://www.nanostring.com/products/nSolver). nsolver (advanced analysis 2.0) data analysis package was used for normalization, cell type analysis, and differential gene expression.

#### 2.1.5 RNAscope

RNAscope is a recent RNA *in situ* hybridization (ISH) method for FFPE tissues, which represents a robust alternative to immunohistochemical techniques in case of the absence of reliable antibodies.

As previously described [21], three micrometre tissue sections were used for RNA ISH. *In situ* detection of SALL4 transcripts was performed with RNAscope 2.5 Assay for Ventana Discovery Ultra system (Advanced Cell Diagnostics, Hayward, CA, USA) using RNAscope 2.5 vs Reagent kit—RED (ACD #310036). After deparaffinization (ACD #323742) and cell conditioning with Universal Target Retrieval V2 (ACD #323741) slides were maintained at 97 °C for 40 min and treated with protease (RNAscope 2.5 vs mRNA pretreat 3‐Protease, #322218) at 37 °C for 16 min. SALL4 probes (RNAscope 2.5 vs probe Hs‐ SALL4, ACD Bio, #505709) were hybridized for 2 h (hardcoded) at 43 °C. To ensure results interpretability, a positive control probe (RNAscope 2.5 vs PPIB #313909) and a negative control probe (RNAscope 2.5 vs DapB #312039) were used.

SALL4 RNA expression was quantified according to the methodology provided by Advanced Cell Diagnostics. Using qupath [20], we evaluated the percentage of CAF within each bin characterized by a number of dots: negative = no staining or < 1 dot/10 cells; positive = ≥ 1 dot/cell. The evaluation was carried out for each case on two representative areas of 1 mm^2^.

### 2.2 *In vitro* experiments

#### 2.2.1 Cell lines

MRC5 human lung fibroblast cells (RRID:CVCL_0440) were purchased from RD Biotech (Besançon, France). Colo320 colon cancer cells (RRID:CVCL_1989) were purchased from ATCC (American Type Culture Collection, Manassas, VA, USA). MRC5 and Colo320 cell lines were cultured in Roswell Park Memorial Institute medium (RPMI 1640) supplemented with 10% of heat inactivated fetal calf serum and 1% penicillin plus streptomycin (Gibco, Illkirch, France). Panc‐1 pancreatic cancer cells (RRID:CVCL_0480) were kindly provided by IGBMC (Institute of Genetics and of Molecular and Cellular Biology, Illkirch, France). BPC8 cell lines were generated in our laboratory from ascites of patients with pancreatic ductal adenocarcinoma. Panc‐1 and BPC8 were cultured in Dulbecco's Modified Eagle Medium (DMEM; Gibco) supplemented with 10% of heat‐inactivated fetal calf serum and 1% penicillin plus streptomycin (Gibco). Cells were cultured at 37 °C in a humidified atmosphere of 5% CO_2_ in air. All cells were periodically authenticated by morphologic inspection. All cell lines were routinely tested for *Mycoplasma* by PCR.

#### 2.2.2 Cell transduction and treatments

The lentiviral particles were produced from a HEK293 packaging cell line that had been co‐transfected with human SALL4 (NM_020436) expressing pLVX‐IRES‐zsGreen plasmid from GeneCust (Boynes, France), pMD.G and Pax2. Forty‐eight hours after transfection, the viral supernatant was collected and stored at −80 °C. MRC5 cell line was transduced with the viral supernatant. For TGF‐β1 treatment, MRC5 were incubated with 10 ng·mL^−1^ TGF‐β1 (100‐21C; Peprotech, Cranbury, NJ, USA) for 48 h before treatment with 50 ng·mL^−1^ Netrin‐1 (6419‐N1; R&D Systems, Minneapolis, MN, USA) for 4 days. MRC5 were used between passages 23–28 for all experiments: western blotting, colony formation assay, spheroid culture, and RT‐qPCR.

#### 2.2.3 Western blotting

Cellular proteins were separated by electrophoresis (SDS/PAGE) in a polyacrylamide gel in presence of sodium dodecylsulfate (SDS) according to their molecular weight, then transferred to a polyvinylidene difluoride film (PVDF) for staining by a specific antibody. Antibodies used were mouse anti‐human SALL4 (1/100) (sc‐101 147; Santa Cruz, Heidelberg, Germany), and mouse anti‐actin (1/1 000 000) (A5441; Sigma, Darmstadt, Germany).

Blotted proteins were detected and quantified on a bioluminescence imager and bio‐1d advanced software (Vilber‐Lourmat, Collégien, France) after blots were incubated with horseradish peroxidase–conjugated appropriate secondary antibody.

#### 2.2.4 Colony formation assay

The effect of SALL4 expression on colony formation *in vitro* was evaluated by soft agar colony formation assay. Eight thousand cells of BPC8 or Colo320 ± 16 000 MRC5^ctrl^ and MRC5^SALL4^ per well were seeded in 500 μL of 0.5% agarose medium in a 24‐well plate. Cells were incubated at 37 °C, 5% CO_2_. Photos were taken and colony number was counted after 10 days of culture.

#### 2.2.5 Generation of 3D pancreatic spheroid and cell isolation

For the generation of 3D spheroids, pancreatic tumor cells were seeded on a round‐bottomed 96‐well plate with ultralow attachment coating (174925; ThermoFisher, Illkirch, France) in RPMI medium at a density of 4000 cells per well for Panc‐1 in a volume of 100 μL and with a ratio of 1 : 2 for pancreatic cells: MRC5 heterospheroids. Panc‐1 spheroids were seeded with 3 μg·mL^−1^ of collagen I (A1048301; ThermoFisher). After 7/10 days of incubation at 37 °C in a humidified 5% CO_2_ atmosphere, spheroids were processed for other experiments. The size of the spheroids was monitored three times per week with image processing (http://imagej.1557.x6.nabble.com/A‐macro‐for‐automated‐spheroid‐size‐analysis‐td5009205.html).

For the preparation of cell suspensions, spheroid cells were collected after 10 days of culture and separated by trypsin digestion. Next, cells were passed through a 70‐μm nylon cell strainer and resuspended in flow cytometry buffer (PBS, 2% FBS with 2 mm EDTA). Cells were separated using magnetic microbeads for APC and an APC‐EPCAM staining (APC MicroBeads; Miltenyi, Bergisch Gladbach, Germany). Tumor cells corresponded to EPCAM positive fraction, and fibroblast population corresponded to the negative fraction. Trypan blue was used to distinguish viable cells before staining, then cells were used for flow cytometry analysis. After separating tumor cells to fibroblast, each population was used to RT‐qPCR, flow cytometry, immunofluorescence, and immunohistochemistry.

#### 2.2.6 RNA isolation, reverse transcription, and real‐time quantification

Total RNA was isolated using RNeasy Mini Kit (Qiagen). Reverse transcription was performed with High‐Capacity RNA‐to‐cDNA Reverse Transcription Kit according to the manufacturer's instructions (Applied Biosystems, Foster City, California, USA). Quantitative PCR (qPCR) was performed using gene‐specific TaqMan probes (*SALL4 [Hs04935855_g1] for transfected cells and [Hs05386497_g1] for other cells, SOX2 [Hs00602736_g1], OCT3/4 (POU5F1) [Hs04195369_s1], NANOG [Hs02387400_s1], SOX11 [Hs00846583_s1], MEIS3 [Hs00908777_m1], and UNC5B [Hs00900710_m1]*; ThermoFisher Scientific) and TaqMan Universal Master Mix II (Applied Biosystems), following the manufacturer's instructions. Gene expression was normalized to glyceraldehyde‐3‐phosphate dehydrogenase (*GAPDH*). Samples were realized in duplicate. Relative expression for the mRNA transcripts was calculated using the $2^{-\Delta \Delta C_{t}}$ method.

### 2.3 Bioinformatic databases

The publicly available datasets (open data source) of pancreatic cancer including at least 145 samples of localized adenocarcinoma with transcriptomic (microarray or RNA‐sequencing), clinical, and survival data were selected. Samples with a metastatic stage or non‐pancreatic tumor (cell lines, normal tissue, or other localizations) were excluded.

The development cohort was downloaded from the ICGC (International Cancer Genome Consortium) data portal (dcc.icgc.org, release 26), containing microarray gene expression of 259 treatment‐naïve resected PDAC [2]. The Cancer Genome Atlas (TCGA) project (RNA‐sequencing: TCGA_PAAD [22]) by the Broad Institute platform (https://gdac.broadinstitute.org, release 20160128, already normalized, level 3) and the National Center for Biotechnology Information Gene Expression Omnibus (NCBI GEO) repository (microarray: GSE85916 [5]), were chosen for validation cohorts.

The transcriptomic data resulted from different technologies (Illumina and Affymetrix) and were summarized in Table S2. For downstream analyses, we downloaded previously normalized, by quantile method for the ICGC and robust multi‐array average for the GSE85916.

#### 2.3.1 Mutational profile

The somatic mutations detected in 224 samples were downloaded from the ICGC project. The mutation dataset for 78 SALL4 positive samples and 146 SALL4 negative samples was available. The r package “maftools” was used for mutation spectrum visualization [23].

#### 2.3.2 Gene expression analysis

In each cohort of microarray data, a single probe analyzed the SALL4 expression and detected its two isoforms (SALL4A and SALL4B) (Table S3). For genes with several probes, the expression was evaluated from the mean of duplicate probes.

SALL4 expression was correlated (Pearson correlation) to the expression of each gene. The resulting *P*‐values were adjusted for multiple testing using the approach of Benjamini and Hochberg. The cut‐offs were adjusted *P*‐value < 0.05 and correlation coefficient > 0.4 for the ICGC and TCGA cohorts, and > 0.3 for the GSE85916. The intersected genes between these three cohorts were kept to identify the most relevant genes and define the SALL4 signature. The scaled expression of each relevant gene was used for clustering and heatmap visualization, using the r package “pheatmap”.

#### 2.3.3 Pathway analysis

To investigate the association between SALL4 expression and stemness phenotype, we used the Kyoto Encyclopedia of Genes and Genomes (KEGG) pathway namely “hsa04550” (Signaling pathways regulating pluripotency of stem cells). KEGG pathway and GeneOntology (GO) Biological Process (BP) enrichment analyses were performed using david software (https://david‐d.ncifcrf.gov/). The cut‐off was adjusted *P*‐value (FDR) < 0.01.

#### 2.3.4 Molecular and cancer‐associated fibroblasts subtypes of pancreatic cancer: published classifications

The molecular subtype labels from the Puleo classification were available in the GSE85916 dataset [5]. The gene expression signatures for each of the five subtypes were selected from a differential analysis based on a one‐versus‐all comparison, using the r package “limma” [24]. The 1000 most differentially expressed genes of each subtype were used as gene signatures. These signatures were used to build independent centroids for each subtype. The subtypes were assigned to the samples of the ICGC and TCGA datasets, by correlating the centroids to the transcriptomic profile of each patient, using Pearson correlation analysis.

Then, basal and classical subtypes were called based on gene expression levels of gene sets described in Moffitt et al. [3]. Consensus clustering was performed based on the correlation distance of the 50 gene signatures. Dendrograms were then cut and branches labeled semiautomatically to assign subtypes to samples consistent with their respective expression patterns.

The four pCAFassigner subtypes (A, B, C, and D) were taken from Table S4 of the article by Neuzillet et al. [25]. The dominant subtype labels were available for 62 samples among the selected samples profiled by whole‐transcriptome RNA sequencing in the ICGC database. The gene expression signatures for each CAF subset were identified by the same method as described above and applied to the 259 patients in the microarray set.

#### 2.3.5 Tumor microenvironment profile

The MCP‐counter algorithm was used to infer the tissue‐infiltrating immune and fibroblast proportions of the tumor microenvironment. The MCP‐counter consists of eight immune and two stromal cell populations (fibroblasts, endothelial cells) and was constructed from gene expression profiles of these cell types [26].

Several of the stromal TGF‐β response signatures, including fibroblasts (F‐TBRS), were identified in cohorts of patients with colorectal cancer [27, 28]. To evaluate the subtype of myofibroblasts, this F‐TBRS signature was applied in pancreatic cancer datasets. Another TGF‐β CAF signature was recently published and evaluated in our article [29].

Then, RNA subtypes clustering was performed based on the correlation distance of 12 gene signatures from different classifications [26, 29, 30, 31] and SALL4 signature. Dendrograms were then cut and branches labeled semiautomatically to assign four subtypes to samples consistent with their respective expression patterns. These cluster‐based subtype assignments were performed blinded with respect to all sample characteristics or clinical data.

#### 2.3.6 Pancreatic intraepithelial neoplasia (PanIN) dataset

PanIN dataset was downloaded previously normalized from the provided GEO entries GSE43288. The r package “limma” was used for differential gene expression (DGE) analysis between PDAC and PanIN, and PDAC and normal pancreas. The criteria for DGEs were log_2_ fold‐change (log_2_FC) > 1 (or < −1) and adjusted *P*‐value < 0.05 [24]. The intersected genes between these two DGEs and the SALL4 signature were kept to identify the most relevant genes for clinical application. The expression (scaled and clustered) of each relevant gene was used for heatmap visualization, using the r package “pheatmap”.

#### 2.3.7 PDAC single‐cell RNA‐sequencing data processing

Data from 24 PDAC patients and 11 control pancreas tissues were obtained from the Genome Sequence Archive under project PRJCA001063 in FASTQ format. Reads were processed using cell ranger 3.1.0 using default parameters and supplying a custom reference package based on human reference genome GRCh38. Samples of 22 patients and 11 control tissues for which the correct chemistry was detected by cell ranger (10x Genomics, Pleasanton, CA, USA) from the sequencing data were used for downstream analysis.

Subsequent data analysis was carried out in r 3.6.2 and the seurat package (v 3.1.2). Low‐quality cells (< 300 genes/cell, < 3 cells/gene, and > 5% mitochondrial genes) were excluded. To remove noise from droplets containing more than one cell, we focused on cells with at most 5000 measured genes, keeping 72 694 cells for further analyses. Subsequently, data were normalized to log(CPM/100 + 1) and scaled regressing out the number of distinct UMIs and the fraction of mitochondrial reads during scaling. The normalization and scaling of the data were performed using the functions NormalizeData (method “LogNormalize”) and ScaleData implemented in Seurat.

Dimensionality reduction was carried out with the Seurat package [32]. We identified the 2000 most variable genes and applied principal component analysis to cells in this gene space. Principal components 1–20 were provided as an input for dimensionality reduction via t‐Distributed Stochastic Neighbor Embedding (tSNE) with default parameters. Clusters of cells were identified based on a shared‐nearest neighbor (SNN) graph between cells and the smart moving (SLM) algorithm (resolution = 0.5). Markers for each cluster were identified by reducing the number of candidate genes to those genes which were (a) at least log(0.25)‐fold higher expressed in the cluster under consideration compared to all other clusters and (b) expressed in at least 10% of cells in the cluster under consideration. Then, the cluster‐specific marker genes were identified as in a previous study [33].

### 2.4 Statistical analysis

Overall survival (OS) was calculated from the date of cancer diagnosis to the date of death from any cause. Survival data were censored at the last follow‐up. OS was estimated using the Kaplan–Meier method and described using median or rate at specific time points with 95% confidence intervals (CI), and compared using the log‐rank test. A potential nonlinear relationship between SALL4 expression and OS was investigated using the restricted cubic splines method with graphical evaluation.

**Fig. 1:**
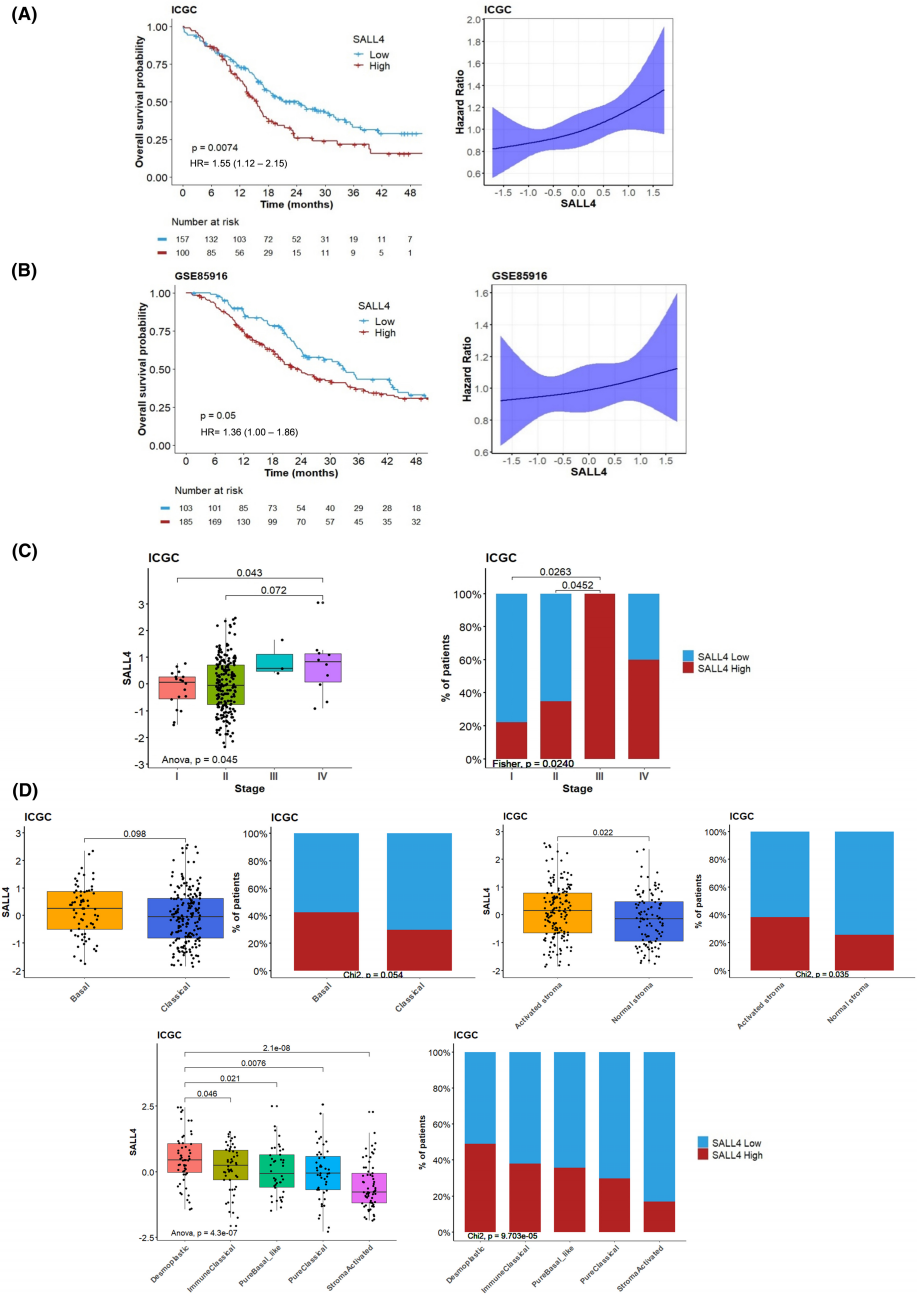
*SALL4* expression is a prognostic biomarker in pancreatic ductal adenocarcinoma. (A) Kaplan–Meier curves of overall survival for 257 patients with localized pancreatic carcinoma in the ICGC cohort. Groups were split by high (red) or low (blue) levels of *SALL4* expression (best cutoff) and compared using the log‐rank test. Hazard ratio modelization with 95% confidence intervals by restricted cubic spline according to *SALL4* expression. (B) Kaplan–Meier curves of overall survival for 288 patients with localized pancreatic carcinoma in the GSE85916 cohort. Groups were split by high (red) or low (blue) levels of *SALL4* expression (best cutoff) and compared using the log‐rank test. Hazard ratio modelization with 95% confidence intervals by restricted cubic spline according to *SALL4* expression. (C) Boxplots and barplots comparing the distribution of the *SALL4* expression in different tumor stages in the ICGC cohort, including advanced stages (stages III and IV, *n* = 13 available patients). (D) Boxplots and barplots comparing the distribution of the *SALL4* expression in basal/classical and activated/normal stroma subtypes from the Moffit classification [3] and Puleo classification [5] for 259 patients with localized pancreatic carcinoma in the ICGC cohort. Boxplots show the median and interquartile range. Medians were compared using Student's *t*‐tests.

Various thresholds (median, tertiles, and quartiles) were evaluated to identify different groups according to the SALL4 expression. To give a reasonable spread of risk, we distinguish two prognostic groups according to the maximizing of the log‐rank test, which was determined following the Hothorn and Lausen method (r package “maxstat” [34]). The SALL4 gene expression assumed a normal distribution and showed no evident cohort‐bias clustering (Fig. S1A). Besides, the potential linear relationship between SALL4 expression and OS was investigated by the restricted cubic splines method with graphical evaluation in the ICGC and GSE85916 cohorts (Fig. 1A,B). These observations allowed to divide the expression levels into intervals containing the same number of data (quantile). Therefore, these analyses let us select the upper tertile cut‐off (threshold value = 5.18) to categorize patients into two risk groups, such as one‐third of the samples had a high SALL4 expression (SALL4^high^), and 67% had a low SALL4 expression (SALL4^low^) (Fig. S1B).

Median value (interquartile range) and frequency (percentage) were provided for the description of continuous and categorical variables, respectively. Medians were compared using Student's *t*‐tests or ANOVA, and proportions were compared using chi‐square tests (or Fisher's exact test, if appropriate). Pearson's test was applied to determine the correlation between gene or signature expressions.

Cox proportional hazard models were performed to estimate hazard ratio and 95% CI for factors associated with OS. The association of the baseline parameters with OS was first assessed using univariate Cox analyses, and then parameters with *P* values of < 0.05 were entered into a final multivariable Cox regression model.

All analyses were performed using sas version 9.4 (SAS Institute, Cary, NC, USA), r software version 3.6.2 (R Development Core Team, Vienna, Austria; http://www.r‐project.org), and graphpad version 8.1.2 (GraphPad Software, San Diego, California, USA). *P* values < 0.05 were considered statistically significant, and all tests were two‐sided.

## 3 Results

### 3.1 SALL4 expression is a prognostic biomarker in PDAC

Among stemness‐related transcription factors, SOX2 and SALL4 were independently associated with a shortened patient survival (Table S3). However, the prognostic relevance of the SALL4 expression in PDAC and the mechanisms governing SALL4 expression in such cancers have so far not been thoroughly investigated.

Here we examined how SALL4 expression predicts the OS of patients with localized PDAC. Patients from the ICGC cohort were categorized into two groups according to SALL4 gene expression levels, based on the “maxstat” method (best performing threshold). The median OS was 24.5 months in the SALL4^low^ group (95% CI = 18.0–32.3 months), and 15.6 months (95% CI = 13.4–18.1 months) in the SALL4^high^ group (Fig. 1A). High expression of SALL4 also conferred poor survival outcomes, in a validation cohort (GSE85916; Fig. 1B). We then identified the upper tertile cut‐off as the threshold closest to the maxstat threshold and transposable to other datasets (Supplementary method and Fig. S1). This threshold followed previous observations in all solid tumors [16] and was applied for downstream analyses.

Localized PDAC patients' characteristics are summarized in Table S4 according to SALL4 expression. SALL4 expression differed only according to the stage (*P* = 0.04). We found no association between SALL4 levels and other clinicopathological factors (Table S4). Moreover, in the overall population including patients with metastatic PDAC, increased SALL4 expression was significantly associated with advanced diseases (Fig. 1C). Thereby, we identified a high SALL4 expression level as a poor prognostic factor for the survival of PDAC patients.

### 3.2 SALL4^high^ PDAC subset does not display a specific mutational profile

Genomic studies have revealed that some key molecular alterations drive the pathogenesis of PDAC, such as KRAS driver mutation and frequent inactivation of TP53, SMAD4, and CDKN2A tumor suppressors [2]. Genomic alterations of open‐source cohorts were retrieved to assess the mutational pattern associated with SALL4 expression, except the copy number variation. In the ICGC cohort, the distribution of genomic alterations did not differ according to the SALL4^high^ (94.9%) and SALL4^low^ (94.5%) tumors. Missense mutations were the main alterations highlighted for the KRAS gene and were well‐balanced between both SALL4 subsets. TP53, SMAD4, CDKN2A, and BRCA1/2 alterations were also distributed similarly whatever SALL4 expression level (Fig. S2A). Of note, the tumor mutation burden was similar between the SALL4^high^ and SALL4^low^ groups (Fig. S2B). The distribution of highly altered genes in PDAC such as KRAS, TP53, and CDKN2A was also similar in PDAC subsets defined by SALL4 expression level in the validation cohorts (GSE85915 and TCGA; Table S5). These results confirmed that genomic alteration patterns were not different between SALL4^high^ and SALL4^low^ expression in PDAC.

### 3.3 SALL4 expression has a better performance to predict patients' risk of death than PDAC molecular classifications

Recently, transcriptional classifications of PDAC including distinct subtypes associated with different molecular pathways have contributed to better‐characterizing PDAC oncogenesis [2, 5]. In particular, Moffitt et al. [3] identified two tumor‐ and two stromal‐specific subtypes: “basal‐like” tumors or with “activated stroma” have a worse prognosis, compared to the “classical” subset or with “normal stroma”. Puleo et al. [5] determined three tumor‐specific subtypes and also two stromal‐specific subtypes to include the effect of the microenvironment. We investigated the distribution of SALL4 expression among these classifications: all subtypes displayed a heterogeneous expression of SALL4 in ICGC and TCGA cohorts, with enrichment in “activated stroma” and “desmoplastic” subtypes (Fig. 1D; Fig. S3A).

Subsequently, we evaluated the independent prognostic value of SALL4 expression compared with clinical and pathological factors. Using univariate Cox analyses in the ICGC cohort, five parameters were identified as prognostic factors for OS, including the molecular classification of Moffitt (tumor subtypes), primary tumor site, tumor grade, and SALL4 expression. Only two independent risk factors for OS were determined in multivariable Cox analysis: tumor grade (*P* = 0.01) and SALL4 gene expression (*P* = 0.02) (Table 1).

**Table 1:** Prognostic factors associated with overall survival in univariate and multivariable analyses (*n* = 220) in the ICGC cohort.

Parameters	Univariate analysis				Multivariate analysis	
	No. of patients	No. of events	Hazard ratio (95% CI)	*P*‐value^a^	Hazard ratio (95% CI)	*P*‐value^a^
Demographic parameters						
Age	257	153	1.02 (0.98–1.00)	0.07		
Sex						
Male	143	90	1			
Female	116	63	0.76 (0.55–1.06)	0.11		
Ethnicity						
White	197	9	1			
Asian, Black or African American	19	121	0.89 (0.45–1.75)	0.73		
Missing	42	23				
Smoking status				0.80		
Never smoked	88	58	1			
Stopped smoking	30	17	0.90 (0.62–1.32)			
Still smoking	84	50	1.0.4 (0.61–1.79)			
Missing	56	28				
Pathologic parameters						
Histology						
Adenocarcinoma	183	112	1			
Other histology	41	25	0.80 (0.51–1.25)	0.32		
Missing	34	16				
Stage				0.1		
I	48	8	1			
II	203	126	1.54 (0.75–3.15)			
III	3	3	4.24 (1.12–16.1)			
Missing	34	16				
Primary tumor site						
Head	186	107	1			
Body and/or tail	38	30	0.65 (0.43–0.98)	0.04	0.86 (0.55–1.36)	0.53
Missing	31	16				
Tumor grade				0.002		0.01
G1	10	2	1		1	
G2	136	78	3.56 (0.88–14.5)		3.27 (0.80–13.37)	
G3	71	51	5.99 (1.46–24.6)		5.00 (1.20–20.79)	
G4	4	3	4.84 (0.81–29.0)		4.80 (0.80–28.98)	
Missing	37	19				
Classifications						
Molecular classification (Moffitt – tumor subtypes)						
Classical	191	103	1		1	
Basal	68	51	1.80 (1.28–2.53)	< 0.001	1.41 (0.95–2.09)	0.09
Molecular classification (Moffitt – stroma subtypes)						
Normal	105	65	1			
Activated	154	89	1.17 (0.62–1.18)	0.34		
Molecular classification (Puleo)				0.1		
Pure classical	47	30	1			
Immune classical	50	29	1.00 (0.60–1.68)			
Pure basal‐like	52	29	1.44 (0.86–2.42)			
Stroma activated	65	36	0.73 (0.45–1.19)			
Desmoplastic	55	30	0.98 (0.59–1.65)			
SALL4						
Low	172	97	1		1	
High	85	56	1.44 (1.03–2.01)	0.03	1.51 (1.06–2.16)	0.02

^a^ Cox‐proportional‐hazard models used to estimate the association of the parameters with overall survival. Values of *P* < 0.05 were considered statistically significant and all tests were two‐sided.

Taken together, these findings showed that SALL4 may be considered as an independent factor for prognosis prediction in localized PDAC, beyond available molecular classifications. A better understanding of the prognostic role of SALL4 is required through the identification of the molecular pathways related to this specific PDAC subset. We then decided to characterize the transcriptomic profile of PDAC according to SALL4 expression.

### 3.4 SALL4 expression and its transcriptional signature are correlated with stroma remodeling

The transcriptomic analyses allowed us to investigate the gene expression profile associated with SALL4^high^ level in PDAC. We identified the list of genes correlated with SALL4 expression in each cohort (ICGC, TCGA, and GSE859216). Genes with the highest correlation levels were selected and reported in Table S6.

**Fig. 2:**
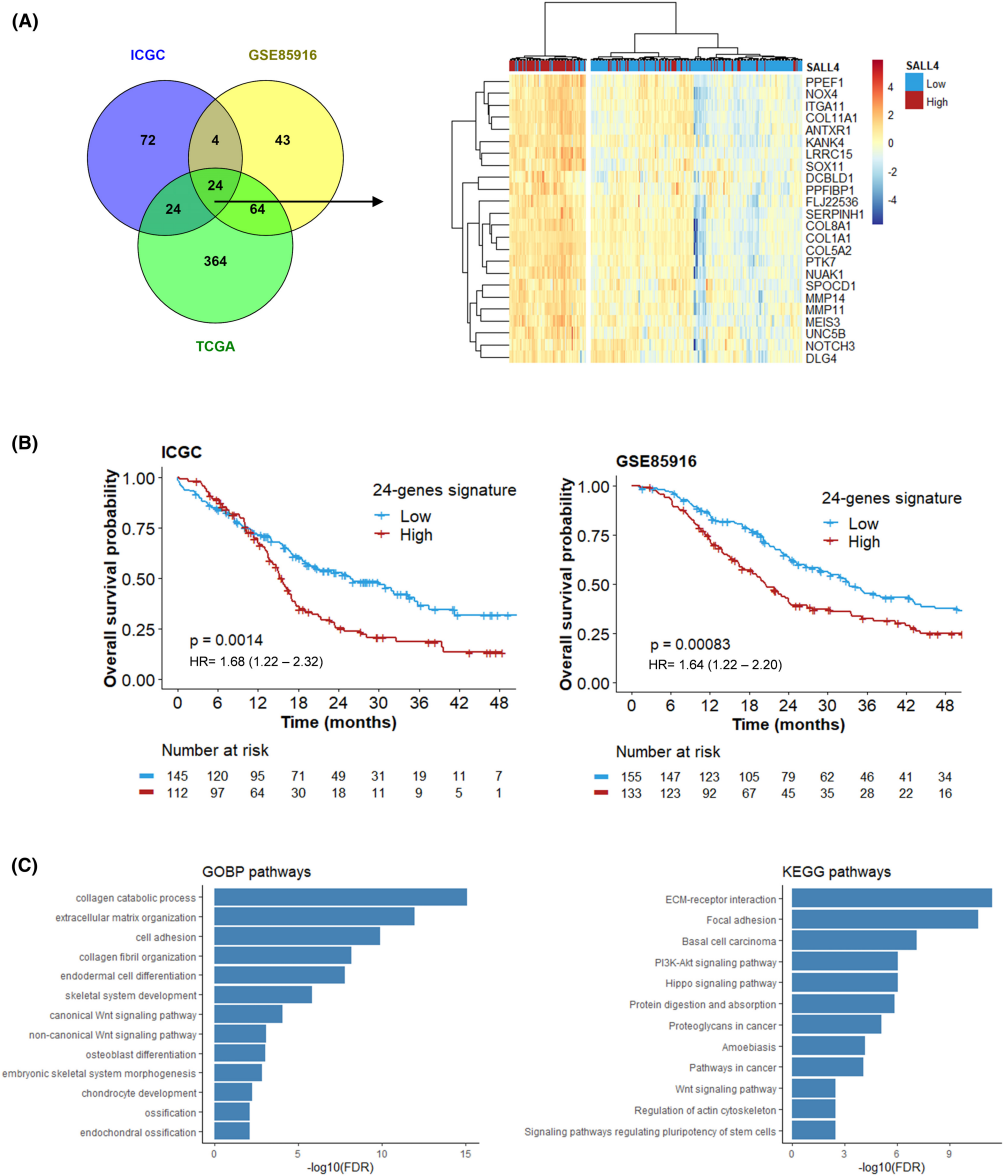
*SALL4* expression and its transcriptional signature are characterized by stromal pathways in pancreatic ductal adenocarcinoma. (A) Venn diagram displaying the number of genes correlated with *SALL4* expression that was intersected between the ICGC, TCGA, and GSE85916 cohorts. Heatmap visualizing the relative average expression of indicated genes (rows) according to *SALL4* expression for 259 patients with localized pancreatic carcinoma in the ICGC cohort. (B) Kaplan–Meier curves of overall survival according to 24‐genes signature expression (best cutoff), compared using the log‐rank test, in ICGC and GSE85916 cohorts. (C) Over‐representation analysis (ORA) of genes correlated with *SALL4* expression from KEGG pathways and GeneOntology (biological process; FDR < 0.01).

Among all genes correlated with SALL4, 24 genes enriched in the SALL4^high^ PDAC subset had expression overlapping in the three cohorts (Fig. 2A) and were selected for further explorations. The involved genes included ANTXR1, CASC15, COL1A1, COL5A2, COL8A1, COL11A1, DCBLD1, DLG4, ITGA11, KANK4, LRRC15, MEIS3, MMP11, MMP14, NOTCH3, NOX4, NUAK1, PPEF1¸ PPFIBP1, PTK7, SERPINH1, SOX11, SPOCD1, and UNC5B. The heatmap displayed that this signature composed of these 24 intersected genes was relevant to distinguishing the SALL4^high^ and SALL4^low^ patients (Fig. 2A; Fig. S4A). The prognostic value of SALL4‐related 24‐gene signature was also investigated. Patients with high level of 24‐gene signature showed a poor prognosis (median OS = 15.6 months [95% CI = 13.9–17.1 months] and 20.5 months [95% CI = 16.9–24.1 months], in ICGC and GSE85916 cohorts, respectively; Fig. 2B).

Functional enrichment analyses revealed the main features observed in SALL4^high^ tumors (Fig. 2C): embryologic development and pluripotency of stem cells (MEIS3 and SOX11), Wnt signaling pathway (ANTXR1 and PTK7; TWIST1 [SALL4‐correlated gene in two cohorts; Table S6]), extracellular structure organization and regulation (NUAK1, MMP11, MMP14, ITGA11, SERPINH1). The extracellular matrix (ECM) includes fibrillar collagens (e.g. COL1A1, COL5A2, COL11A1) as the major connective tissue components and tumor microenvironment. Due to a significant stromal enrichment in the SALL4^high^ PDAC subset, we hypothesized that SALL4 could exert an action to draw a specific molecular feature leading to PDAC invasiveness.

### 3.5 SALL4^high^ PDAC subset exhibits an enrichment for gene expression belonging to fibroblasts and stemness‐related signaling pathways

**Fig. 3:**
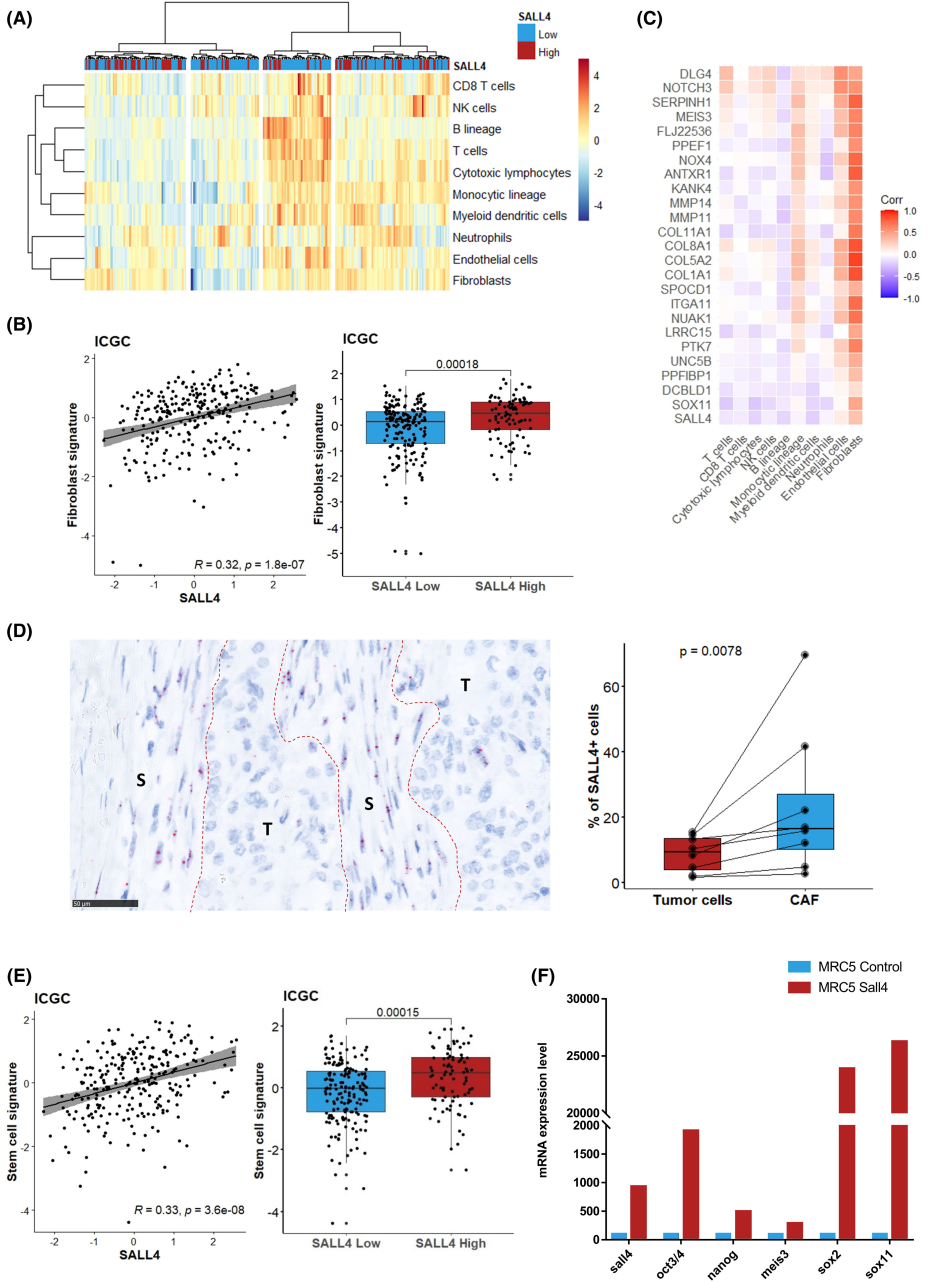
SALL4 gene expression correlates with fibroblasts and stemness phenotype. (A) Heatmap visualizing the relative average expression of MCP‐counter signature (rows) according to *SALL4* expression for 259 patients with localized pancreatic carcinoma in the ICGC cohort. (B) Scatter plot and boxplots comparing the distribution of the fibroblasts signature to the *SALL4* expression. (C) Correlation matrix showing Pearson's correlation coefficients from comparisons between genes of the *SALL4* signature and MCP‐counter signatures in the ICGC cohort. (D) SALL4 RNA expression in tumor (T) and stromal (S) compartments from eight localized pancreatic carcinomas by RNAscope (scale bars, 50 μm). Percentage of SALL4^+^ (≥ 1 dot/cell) cells. (E) Scatter plot and boxplots comparing the distribution of the stemness signature to the *SALL4* expression in the ICGC cohort. Boxplots show the median and interquartile range. Medians were compared using Student's *t*‐tests. (F) mRNA levels of SALL4, SOX2, OCT3/4, NANOG, SOX11, and MEIS3 determined by RT‐qPCR in MRC5^ctrl^ and MRC5^SALL4^ using lentivirus‐mediated transduction for one of a representative experiment (*n* = 2).

Then, we next sought to define the stromal composition related to SALL4 expression in PDAC. The average expression of the immune cell populations, such as T cells (*P* = 0.009), CD8^+^ T cells (*P* < 0.0001), cytotoxic lymphocytes (*P* = 0.004), and natural killer cells (*P* = 0.04), was lower in the SALL4^high^ subset. Similarly, myeloid dendritic cell‐related gene expression was also under‐expressed (*P* = 0.002; Fig. 3A; Fig. S4B). Conversely, this analysis highlighted a significant upregulation of the fibroblast‐related gene expression in SALL4^high^ PDAC (Fig. 3B; Fig. S3B). Among the micro‐environmental pathways, the fibroblast signature showed a strong positive correlation with the 24 genes related to SALL4 in our previous analyses (Fig. 3C). This association was confirmed in the TCGA and GSE859216 cohorts (Fig. S4C). The distribution of SALL4 expression was then assessed at the single cell level by analyzing tumor and stromal cells from patients with PDAC using *in situ* hybridization (RNAscope). The results revealed that SALL4 was expressed in both tumor cells and CAF. Of note, the expression of SALL4 was higher in CAF than tumor cells (*P* = 0.008; Fig. 3D).

Moreover, SALL4 is required for maintaining embryonic stem cell pluripotency and self‐renewal. Indeed, Zhang et al. [9] have highlighted a reduction of other stemness‐related transcription factors OCT4, SOX2, and NANOG in a condition of SALL4 knockdown. Our findings confirmed that SALL4 expression was correlated with signaling pathways regulating pluripotency of stem cells, surrogated by transcription factors such as OCT3/4 and SOX2 (Fig. 3E). In the same way, the SALL4^high^ subset was enriched in stemness signature (Fig. 3E; Fig. S3C). The correlation between SALL4, SOX2, OCT3/4, and NANOG expression was confirmed using lentivirus‐mediated transduction of SALL4 into MRC5 (Fig. S5A). MRC5^SALL4^ but not MRC5^ctrl^ showed increased expression of SOX2, OCT3/4, and NANOG (Fig. 3F). Overall, stemness properties, as well as stroma/fibroblast enrichment, were the main molecular features defining the SALL4^high^ PDAC subset.

### 3.6 Netrin‐1 and TGF‐β1 collaborate to control SALL4 expression in a subset of CAF‐promoting PDAC stemness properties

We next investigated how SALL4 expression is regulated in CAF. Different subsets of CAF contributing to PDAC microenvironment heterogeneity have been already proposed [25]. A heterogeneous expression of SALL4 was observed in all subpopulations of CAF, but the SALL4 gene was predominantly expressed in the CAF‐A and CAF‐C subtypes (Fig. S3D). The CAF‐A subpopulation is strongly enriched with an “activated stroma” signature and associated with unfavorable survival, whereas the CAF‐C subset is notably characterized by the expression of TEK (as known as TIE2, ANGPT2 receptor) associated with the angiogenesis [25].

**Fig. 4:**
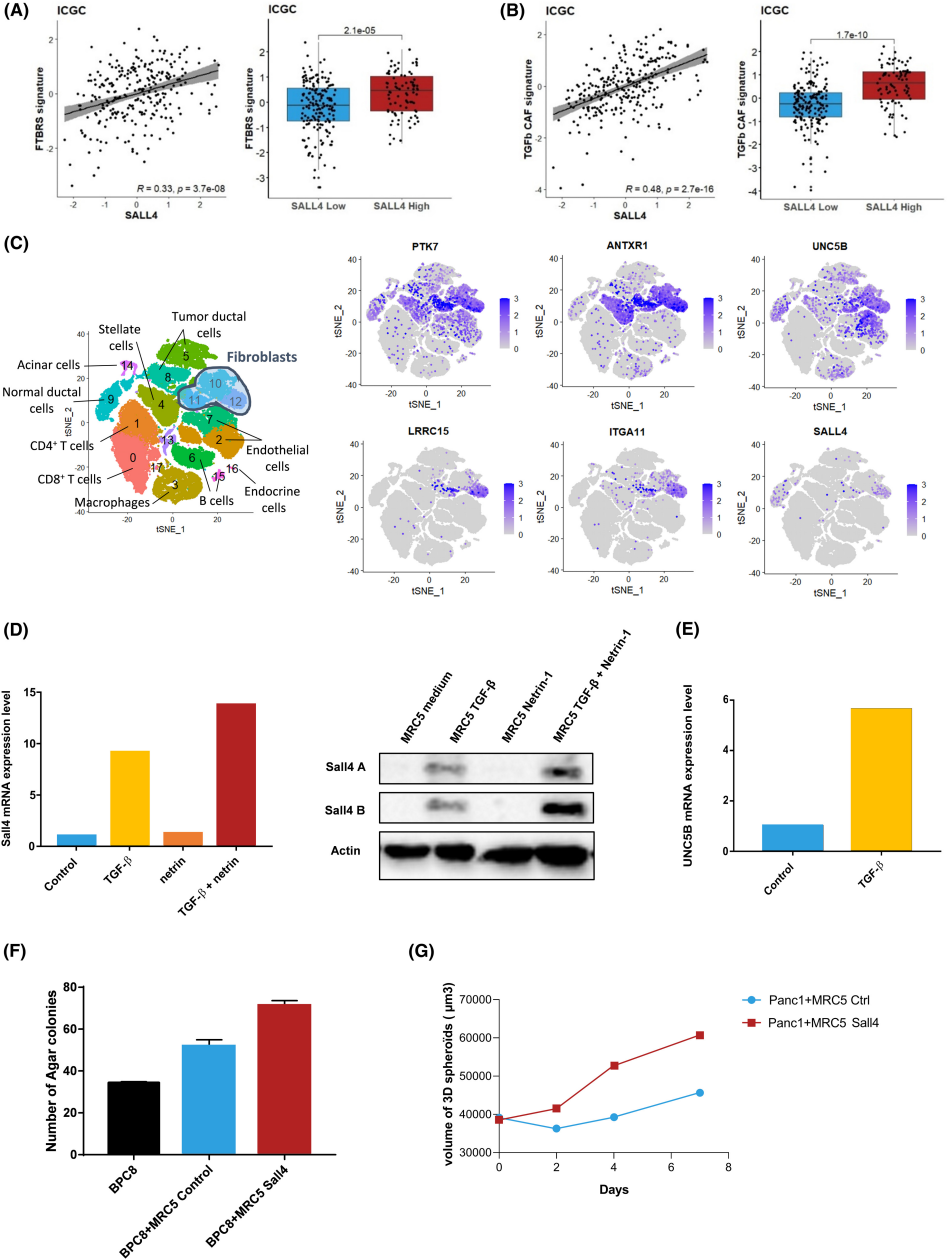
*SALL4* expression identifies a subtype of activated CAF in pancreatic ductal adenocarcinoma. (A) Scatter plot and boxplots comparing the distribution of the F‐TBRS signature [27] to the *SALL4* expression in the ICGC cohort. (B) Scatter plot and boxplots comparing the distribution of the TGF‐β CAF signature (Dominguez et al.) to the *SALL4* expression in the ICGC cohort. Boxplots show the median and interquartile range. Medians were compared using Student's *t*‐tests. (C) t‐Distributed stochastic neighbor embedding (tSNE) embedding of single cells sorted from 33 patients. Clusters identified through graph‐based clustering are indicated by color. (D) mRNA levels of SALL4 determined by RT‐qPCR in MRC5 pre‐treated by TGF‐β1 and/or Netrin‐1 for one of a representative experiment (*n* = 3). Protein expression of SALL4 isoforms (SALL4A: 165 kDa; SALLB: 95 kDa) was evaluated in the same culture condition, with Actin used as an internal control. (E) mRNA levels of UNC5B determined by RT‐qPCR in MRC5 pre‐treated by TGF‐β1 for one of a representative experiment (*n* = 3). (F) Number of colony formation with BPC8 ± MRC5 with or without SALL4 transduction using soft agar colony formation assay. Data are displayed as mean ± standard deviation. One representative experiment is shown (*n* = 3). (G) Size of the spheroids with Panc‐1 and MRC5 with or without SALL4 transduction (mean of triplicates). One representative experiment is shown (*n* = 2).

Furthermore, SALL4 expression was correlated to pan‐fibroblasts TGF‐β response signature (F‐TBRS) in the ICGC cohort (Fig. 4A). Similarly, a positive correlation was ascertained between SALL4 and the TGF‐β CAF signature [29] (Fig. 4B). These observations were also validated in the TCGA and GSE859216 cohorts (Fig. S3E). Interestingly, both F‐TBRS and the TGF‐β CAF signatures were not significantly associated with OS in univariate analysis (Table S7) suggesting that the prognostic role of the SALL4^high^ PDAC subset does not solely rely on the presence of TGF‐β‐driven CAF phenotypes. Likewise, the SALL4‐related 24‐gene signature, stemness, and TGF‐β/CAF molecular networks as well as low expression of immune‐related genes contribute to defining the SALL4 molecular network in ICGC, TCGA, and GSE85916 cohorts (Fig. S4D).

Based on scRNA‐seq performed in cells sorted from PDAC derived from 33 patients [33], Dominguez et al. [29] have identified a predominant cluster of TGF‐β‐activated CAF in human PDAC (Cluster 12; Fig. 4C). Interestingly, half of the genes identified in our study were over‐expressed in this cluster (e.g. ANTXR1, ITGA11, LRRC15, PTK7, UNC5B; Fig. 4C).

Analysis performed in the same scRNA‐seq showed that SALL4 transcription is restricted to a subset of CAF in the LRRC15^high^ cluster where TGF‐β1 driven CAF are located (Cluster 12; Fig. 4C). Consequently, we next addressed if TGF‐β1 could promote SALL4 expression in fibroblasts. MRC5 cell lines were exposed to TGF‐β1 for 6 days. In this first set of experiments, TGF‐β1 increased SALL4 RNA and protein expression in the MRC5 cell line (Fig. 4D). In the second set of experiments, we tested the ability of other SALL4‐related molecules to promote SALL4 expression when combined with TGF‐β1. Stem cell growth factors correlated to SALL4 expression were selected including WNT10A (ligand for ANTXR1, PTK7), Netrin‐1 (ligand for UNC5B), and DLL1 (ligand for NOTCH3). Combination of TGF‐β1 with Netrin‐1 (Fig. 4D), but not with DLL1 or WNT10A (data not shown), further induced SALL4 expression in fibroblasts. This observation is supported by the ability of TGF‐β1 to induce UNC5B expression (Fig. 4E).

The ability of SALL4 to confer oncogenic properties to fibroblasts was assessed in spheroid and colony formation assays. For this purpose, MRC5^ctrl^ and MRC5^SALL4^ were cocultured with the Panc‐1 cell line or with PDAC cells derived from patients' ascites (BPC8), or the Colo320 cell line. MRC5^SALL4^ did not form colonies or exhibit an increased proliferation rate (data not shown). Transduction of SALL4 confers to MRC5 the ability to promote colony formation of pancreatic cancer cells (BPC8) and Colo320 cell line (Fig. 4F; Fig. S5B). Likewise, MRC5^SALL4^ increased the size of the Panc‐1 cell line spheroids compared to MRC5^ctrl^ (Fig. 4G; Fig. S5C). Altogether, these results highlight that TGF‐β1 signaling in cancer stroma interacts with SALL4 transcriptional activity to promote PDAC oncogenic properties.

### 3.7 SALL4‐related stromal signature allows discriminating invasive from pre‐invasive pancreatic lesions and PDAC patients' clinical outcomes

**Fig. 5:**
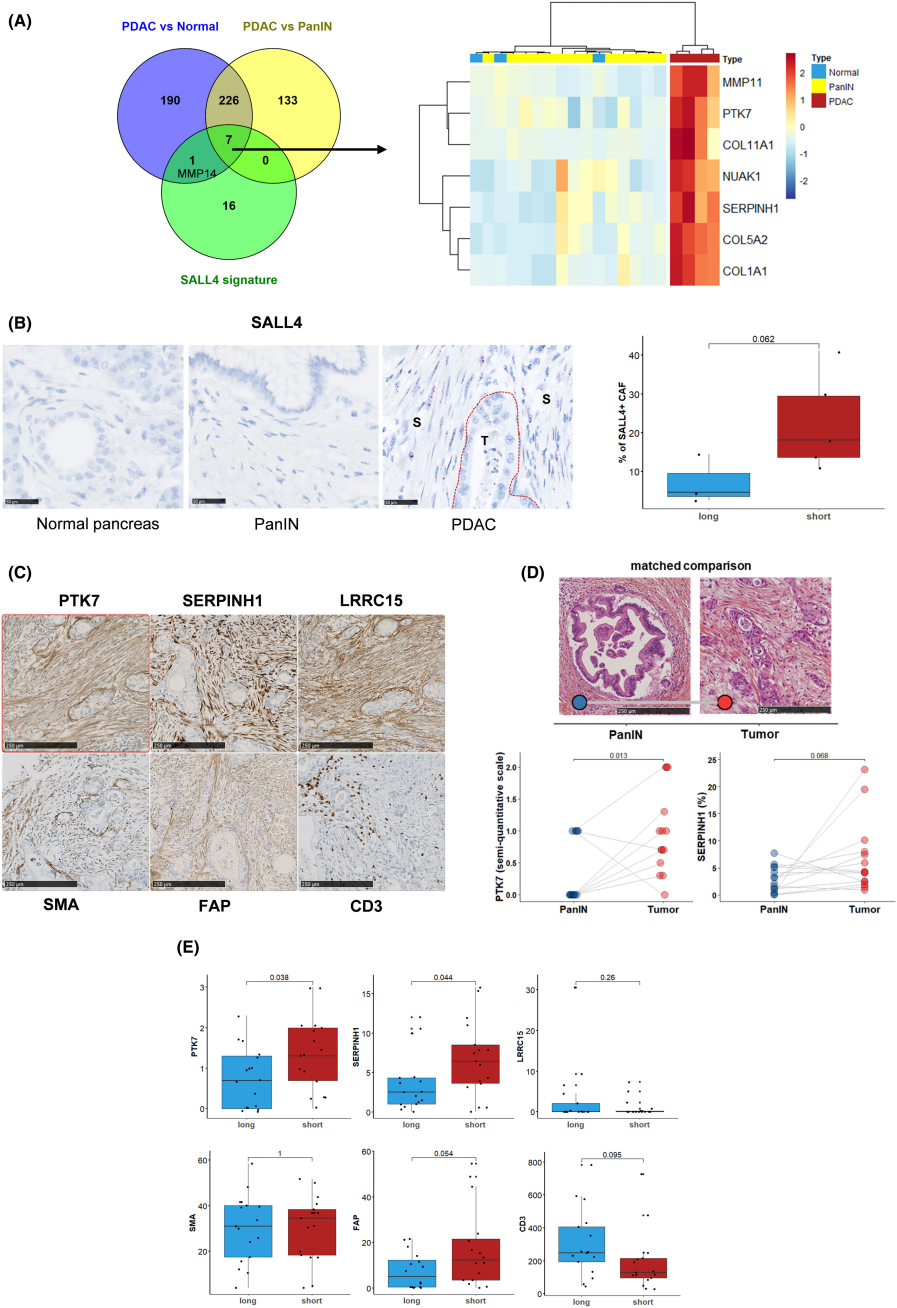
A transcriptional signature is correlated with SALL4 expression with seven genes specific to pancreatic ductal adenocarcinoma. (A) Venn diagram displaying the number of genes correlated with *SALL4* expression that was found specific to pancreatic ductal adenocarcinoma (PDAC), and pancreatic intraepithelial neoplasia (PanIN). Heatmap visualizing the relative average expression of indicated genes (rows) in normal pancreas, PanIN, and PDAC groups. (B) SALL4 RNA expression in pancreas normal (*n* = 6), PanIN (*n* = 5), and PDAC (T, tumor; S, stroma; *n* = 8) compartments from eight localized pancreatic carcinomas by RNAscope (right; scale bars, 250 μm). Percentage of SALL4+ (≥ 1 dot/cell) CAF between short‐term survivors (*n* = 5) and short‐term survivors (*n* = 3) in the same samples (left). (C) PTK7, SERPINH1, LRRC15, SMA, FAP, and CD3 immunohistochemistry staining in a patient with short survival (scale bars, 250 μm). (D) PTK7 and SERPINH1 immunohistochemistry staining in 14 patients with paired PanIN and PDAC samples (scale bars, 250 μm). (E) Immunohistochemistry evaluation in 17 short‐term survivors and 17 long‐term survivors. Boxplots show the median and interquartile range. Medians were compared using Student's *t*‐tests.

Next, we determined the SALL4 transcriptomic activity in the different stages of PDAC carcinogenesis. Data from the GSE43288 cohort were examined to identify genes selectively expressed in pancreatic carcinoma by comparison to preinvasive pancreatic lesions (PanIN) or normal pancreatic tissue. A total of 366 genes were overexpressed in PDAC compared to PanIN samples, and 424 genes were overexpressed in PDAC and not in normal pancreatic tissues. Altogether, seven genes belonged to these two genes lists and the SALL4 signature: COL1A1, COL5A2, COL11A1, MMP11, NUAK1, PTK7, and SERPINH1 (Fig. 5A), pointing out that this SALL4‐related 7‐gene signature discriminated the invasive stage of PDAC compared to PanIN stage and normal pancreatic tissue. Most of these selected genes were highly correlated, especially PTK7 and SERPINH1, and observed in a closed pattern, in the three main cohorts (ICGC, TCGA, and GSE859216; Fig. S6A). The prognostic value of SALL4‐related 7‐gene signature was also investigated in ICGC and GSE85916 cohorts. Patients with high level of 7‐gene signature showed a poor prognosis (median OS = 15.8 months [95% CI = 13.6–20.0 months] and 21.9 months [95% CI = 19.1–27.5 months], in ICGC and GSE85916 cohorts, respectively; Fig. S6B). To validate the potential clinical relevance of this stromal signature, we examined the SALL4‐related signatures in samples derived from a case–control cohort of patients discriminated by their PDAC‐related specific survival using NanoString RNA quantification. Messenger RNA (mRNA) belonging to the SALL4‐related signatures was enriched in patients with short‐term survival (Fig. S6C). The expression of SALL4 in stromal cells was investigated using *in situ* hybridization (RNAscope). SALL4 mRNA was identified in fibroblasts from PDAC but not PanIN or normal pancreatic tissues (Fig. 5B). These results support the development of specific markers to better define the fibroblast characteristics in PDAC samples. Then, we decided to characterize PTK7 and SERPINH1 expression by immunohistochemistry in comparison to Fibroblast activation protein (FAP), alpha‐smooth muscle actin (αSMA), and LRRC15, markers of activated fibroblasts which are induced by TGF‐β1 and characterize myofibroblastic transdifferentiation of CAF [35, 36] (Fig. 5C; Fig. S6D).

To further sustain the correlation between SALL4‐related molecular network and PDAC invasiveness, PTK7 and SERPINH1 expressions were assessed by immunohistochemistry in human PanIN and PDAC samples (Fig. 5D). PTK7, SERPINH1, and FAP could discriminate PanIN versus tumor‐related stroma by contrast to αSMA and LRRC15. Interestingly, while αSMA, FAP, or LRRC15 failed to fully discriminate patients according to their risk of death, PTK7 and SERPINH1 were significantly increased in patients with short survival (Fig. 5E). CD3 was also considered in order to characterize the immune compartment and a high expression was more observed in long‐term survivors (Fig. 5E). Altogether, our results establish that SALL4‐related signaling contributes to define a specific PDAC stroma pattern related to invasiveness and worse clinical outcomes.

## 4 Discussion

We examined the potential clinical impact of stemness‐related transcription factors in PDAC databases. Consistent with results observed in other tumor types, our study pointed out a prognostic value of SALL4 expression in PDAC. Gene expression analyses revealed that the SALL4‐related molecular network defines a PDAC subset enriched in cancer stem cell functions and stromal organization. These findings underscore a direct role of TGF‐β1 in the regulation of SALL4 expression in stromal cells. SALL4 expressing fibroblasts then support the organization of a specific microenvironment promoting *in vitro* colony formation and invasive features in PDAC patients.

One strength here is the characterization of a 24‐gene signature able to predict PDAC OS in different cohorts. The SALL4‐related stromal network might also be of interest in discriminating stromal components associated with pre‐invasive and invasive pancreatic carcinomas. We propose a specific assay based on PTK7 and SERPINH1 detection by immunohistochemistry to better characterize pancreatic carcinoma invasiveness and prognosis.

To date, the molecular pattern of SALL4 expressing PDAC has not been characterized. In this work, SALL4 expression was not associated with genomic alterations (e.g. KRAS, TP53), nor tumor mutation burden. Of note, the copy number variation of tumor suppressor genes was not assessed. These observations raised the hypothesis that the SALL4 gene level is not related to a specific oncogenic pathway in PDAC. Interestingly, even if most of the genomic alterations reported in PDAC are frequently detected at early stages of oncogenesis and in pancreatic precancerous lesions (PanIN) [37], we could observe that the SALL4‐related molecular network occurred in a late stage of oncogenesis. Even if MYC activation in PanIN epithelium was previously reported to instruct PDAC phenotype, with an extensive αSMA–positive desmoplastic stroma, the specific pathways modulating fibroblasts organization during PDAC oncogenesis have not been investigated [38]. In the present work, MYC expression or transcriptional activity was not identified as a discrimination factor between pre‐invasive and invasive pancreatic lesions. It was also previously established that the expression of SALL4 and other stemness genes can be increased under hypoxia, but our investigations have not confirmed this observation [39]. By contrast, our investigations sustain a role for TGF‐β1 in the regulation of SALL4 expression in stromal cells (Fig. 4D).

Altogether, our results suggest that SALL4 expression is acquired lately in PDAC oncogenesis and by contrast with αSMA, seven of the genes included in the SALL4‐related stromal signature (COL1A1, COL5A2, COL11A1, MMP11, NUAK1, PTK7, SERPINH1) could discriminate preinvasive PanIN from PDAC. Pathway enrichment analyses revealed that the SALL4^high^ subset displayed a significant enrichment of ECM‐related genes that suggested a predominant role in stromal support through the production and maintenance of collagen networks. Besides, SERPINH1, also called HSP47, is a chaperone protein involved in modulating collagen production and contributes to EMT and angiogenesis through the TGF‐β pathway [40]. NUAK1, as known as ARK5, is associated with migratory and invasive properties, through stimulation of metalloproteinases (MMP) secretion [41]. MMP, such as MMP11 and MMP14, are involved in degrading the ECM in various cancers and therefore promote metastasis and angiogenesis. Of note, PTK7 is a major cleavage target of MMP14 in the plasma membrane, involved in cell mobility [42]. In addition, the cleavage of ECM components leads to the shedding and activation of molecules such as TGF‐β by MMP14. Collectively, our findings highlighted that the occurrence of a specific stromal pathway in the SALL4^high^ PDAC subset can coordinate pancreatic microenvironment plasticity and drive the transition from PanIN to invasive tumors.

In our study, we showed that SALL4 expression can be induced by TGF‐β in primary human embryonic fibroblasts (Fig. 4D), but not in mesenchymal stem cells (data not shown). Using scRNA‐seq data, we observed a cluster of CAF where TGF‐β1 transcriptional activity is enhanced (cluster 12; Fig. 4C). SALL4^high^ CAF determine a specific subset within this TGF‐β1 dependent cluster of CAF. In addition, the correlation between SALL4 expression and TGF‐β1 activated CAF signatures reported in our study might support the hypothesis that TGF‐β1 and SALL4 crosstalk promotes CAF subpopulation heterogeneity and stromal properties along PDAC oncogenesis. CAF interact with both cancer cells and other stromal cells through a network of signaling pathways and mediators. Recently, two signatures (F‐TBRS and TGF‐β CAF) defining the activation of fibroblasts with TGF‐β were previously reported in colorectal cancer and PDAC, respectively [27, 29]. Both these signatures were characterized by a dominant ECM gene feature, myofibroblastic properties, and were enriched in SALL4^high^ samples. The role of TGF‐β1 in SALL4^high^ CAF differentiation is supported by the presence of several TGF‐β1 regulated genes in the SALL4 signature, especially LRRC15 and ITGA11 [29]. LRRC15 is highly expressed on the cell surface of stromal fibroblasts in many solid tumors and identified as a novel marker of TGF‐β‐activated fibroblasts and mesenchymal stem cells [43]. ITGA11 driven by TGF‐β is an important mediator of EMT and the major collagen‐binding receptor overexpressed by fibroblasts to regulate ECM organization and stromal fibrosis formation [44].

However, the presence of high levels of SALL4 in CAF does not only recapitulate TGF‐β1 activity. One hypothesis raised by the correlation of the stem cell‐related gene and SALL4 signatures was that SALL4‐mediated functions contribute to the stemness properties conferred by TGF‐β1‐driven CAF. Indeed, the SALL4‐related signature also included some genes involved in signaling pathways driving stemness acquisition. In particular, ANTXR1 and PTK7 genes encode receptors involved in Wnt‐pathway activation, through binding the Wnt co‐receptor LRP6, and are markers of stem cells [45, 46]. Next, gene expressions belonging to the SALL4‐related molecular network were similar to a myofibroblastic CAF subset, predominantly associated with triple‐negative breast cancer (CAF‐S1) [47, 48]. Indeed this CAF‐S1 subset displayed also the Wnt signaling pathway and stemness‐related genes: WNT2, WISP2, and GREM1 [49]. In our results, SALL4, as well as SOX2, OCT3/4, and NANOG were increased in TGF‐β1‐activated MRC5 (Fig. 3F). These markers of pluripotency perform a spectrum of functions during embryonic development and may contribute to stromal and tumor cell renewal. Thus, we described that the TGF‐β pathway enhanced stemness in PDAC through the induction of a SALL4‐related molecular network in a subpopulation of fibroblasts.

Another interesting issue of the SALL4‐related molecular networks described in this study is the correlation of these particular stromal features with the expression of UNC5B, a Netrin‐1 receptor. This observation raises the hypothesis that Netrin‐1 might collaborate with TGF‐β1 and SALL4 to promote stemness phenotype in PDAC. In line with this hypothesis, we could observe that the cancer formation property of TGF‐β‐activated fibroblasts was enhanced by Netrin‐1 in colony formation assays. CAF may also be important stromal cells expressing Netrin‐1 [50]. Overexpression of Netrin‐1 was already reported in several cancers including PDAC and was shown to promote invasion and neural migration [51, 52]. As already described, cancer cells in cocultures with CAF upregulate the production of this ligand and UNC5B [50]. Interestingly, Netrin‐1 upregulation was also reported in CAF to be associated with increased cancer cell stemness, through IL6 modulation [50, 53]. Moreover, Netrin‐1 inhibits apoptosis in pluripotent embryonic stem cells by UNC5B [54].

Notwithstanding the presence and nature of CAF in PDAC is an already established prognostic factor in many gene expression analyses, this prognostic factor is still not used in the current management of PDAC patients or to predict the risk of death. Although fibroblasts play a critical role in tissue homeostasis and disease processes, understanding their function has been hampered by their inherent heterogeneity and a lack of robust markers.

Underlined by bioinformatic analyses, the occurrence of a stromal signature characterized the SALL4^high^ PDAC subset which might account for the unfavorable prognosis, in ICGC and GSE85916 cohorts. Unfortunately, TCGA survival features were not applied due to discordant data with the literature and pancreatic cancer statistics [55, 56]. Knudsen et al. [57] have identified three PDAC stromal compartments according to morphological characteristics, including the “immature” stroma with highly cellular and collagen‐poor stroma, associated with shorter OS. Other classifications showed the poor prognostic value of CAF, specifically stroma enriched in αSMA+ fibroblasts compared to stroma enriched in collagen [58, 59]. The PDAC microenvironment plays an important role in tumor growth and progression, and resistance to current systemic therapies [60]. Particularly, the expression in CAF of PRRX1, identified in the SALL4‐related prognostic signature, was associated with chemotherapy resistance in PDAC [61]. One of the main interests of our work is to provide the rational for the development of an immunohistochemical assay where characterization of PTK7 and SERPINH1 in the stroma might recapitulate the transcriptional activity of SALL4‐related stroma in PDAC. We have still reported that PTK7 and SERPINH1 stromal expression contribute to discriminating PanIN against invasive pancreatic carcinoma. Moreover, this immunohistochemistry also discriminated the stromal features of patients selected according to their probability of long‐term remission after surgery. While studies have already shown that SERPINH1 is a stromal marker and confers chemoresistance [62, 63], our study identified PTK7 as a novel marker of fibroblasts in invasive pancreatic carcinomas. Further investigations are mandatory to validate how this assay will allow OS in a prospective cohort of PDAC patients.

## 5 Conclusions

Together, our results show that SALL4 transcriptional activity controls a molecular network characterized by a specific stromal signature enriched in TGF‐β‐activated CAF and defined by a 7‐gene signature and an immunohistochemical assay based on PTK7 and SERPINH1 which might have clinical implementations.

## Conflict of interest

The authors declare no conflict of interest.

## Author contributions

AV, CB conceived the study. AV, FM collected patient cohort. VM performed cell culture, spheroids, and colony formation assay. VM, J‐RP, KA performed RT‐qPCR. AB performed cell transduction and WB. RL performed Nanostring assay. FM, CM, GA performed tissue microarray construction, immunohistochemistry, and RNAscope. AV, CT performed statistical and bioinformatic analyses. AV, FM, CT, VM, AB, J‐RP, CM, RL, KA, GA, FG, FB, PP, CB analyzed the data. AV, CB wrote the manuscript.

### Peer review

The peer review history for this article is available at https://publons.com/publon/10.1002/1878‐0261.13370.

## Supporting information


**Fig. S1.** Determination of the prognostic threshold to split groups according to *SALL4* expression in pancreatic ductal adenocarcinoma.
**Fig. S2.** The landscape of mutations in pancreatic ductal adenocarcinoma according to *SALL4* expression.
**Fig. S3.** Validation of the phenotype of *SALL4* expression in pancreatic ductal adenocarcinoma.
**Fig. S4.** Validation of the *SALL4* signature.
**Fig. S5.** Molecular pathways and functionality of SALL4‐expressing fibroblasts.
**Fig. S6.** Evaluation of stromal signature in immunochemistry.
**Table S1.** Immunohistochemistry antibodies.
**Table S2.** Characteristics of the selected publicly datasets.
**Table S3.** Univariate analyses of different stemness‐related genes for overall survival in the ICGC cohort.
**Table S4.** Patients' characteristics according to *SALL4* expression (using the upper tertile cut‐off) for 259 patients with localized pancreatic carcinoma in the ICGC cohort.
**Table S5.** Mutational profile according to *SALL4* expression for patients with localized pancreatic carcinoma in the GSE85916 and TCGA cohorts.
**Table S6.** List of correlated genes with SALL4 expression in the ICGC, GSE85916, and TCGA cohorts
**Table S7.** Univariate analyses of different pathways for overall survival in the ICGC cohort.

## Data Availability

All data generated or analyzed during this study are included in this published article and its Supporting Information files.

## References

[1] Rahib L , Smith BD , Aizenberg R , Rosenzweig AB , Fleshman JM , Matrisian LM . Projecting cancer incidence and deaths to 2030: the unexpected burden of thyroid, liver, and pancreas cancers in the United States. Cancer Res. 2014;74:2913–21. 10.1158/0008-5472.CAN-14-0155 24840647

[2] Bailey P , Chang DK , Nones K , Johns AL , Patch A‐M , Gingras M‐C , et al. Genomic analyses identify molecular subtypes of pancreatic cancer. Nature. 2016;531:47–52. 10.1038/nature16965 26909576

[3] Moffitt RA , Marayati R , Flate EL , Volmar KE , Loeza SGH , Hoadley KA , et al. Virtual microdissection identifies distinct tumor‐ and stroma‐specific subtypes of pancreatic ductal adenocarcinoma. Nat Genet. 2015;47:1168–78. 10.1038/ng.3398 26343385 PMC4912058

[4] Collisson EA , Sadanandam A , Olson P , Gibb WJ , Truitt M , Gu S , et al. Subtypes of pancreatic ductal adenocarcinoma and their differing responses to therapy. Nat Med. 2011;17:500–3. 10.1038/nm.2344 21460848 PMC3755490

[5] Puleo F , Nicolle R , Blum Y , Cros J , Marisa L , Demetter P , et al. Stratification of pancreatic ductal adenocarcinomas based on tumor and microenvironment features. Gastroenterology. 2018;155:1999–2013.e3. 10.1053/j.gastro.2018.08.033 30165049

[6] Clarke MF , Fuller M . Stem cells and cancer: two faces of eve. Cell. 2006;124:1111–5. 10.1016/j.cell.2006.03.011 16564000

[7] Friedmann‐Morvinski D , Verma IM . Dedifferentiation and reprogramming: origins of cancer stem cells. EMBO Rep. 2014;15:244–53. 10.1002/embr.201338254 24531722 PMC3989690

[8] Mani SA , Guo W , Liao M‐J , Eaton EN , Ayyanan A , Zhou AY , et al. The epithelial‐mesenchymal transition generates cells with properties of stem cells. Cell. 2008;133:704–15. 10.1016/j.cell.2008.03.027 18485877 PMC2728032

[9] Zhang J , Tam W‐L , Tong GQ , Wu Q , Chan H‐Y , Soh B‐S , et al. Sall4 modulates embryonic stem cell pluripotency and early embryonic development by the transcriptional regulation of Pou5f1. Nat Cell Biol. 2006;8:1114–23. 10.1038/ncb1481 16980957

[10] Aguila JR , Liao W , Yang J , Avila C , Hagag N , Senzel L , et al. SALL4 is a robust stimulator for the expansion of hematopoietic stem cells. Blood. 2011;118:576–85. 10.1182/blood-2011-01-333641 21602528 PMC3142902

[11] Gao C , Kong NR , Li A , Tatetu H , Ueno S , Yang Y , et al. SALL4 is a key transcription regulator in normal human hematopoiesis. Transfusion. 2013;53:1037–49. 10.1111/j.1537-2995.2012.03888.x 22934838 PMC3653586

[12] Tatetsu H , Kong NR , Chong G , Amabile G , Tenen DG , Chai L . SALL4, the missing link between stem cells, development and cancer. Gene. 2016;584:111–9. 10.1016/j.gene.2016.02.019 26892498 PMC4823161

[13] Yong KJ , Gao C , Lim JSJ , Yan B , Yang H , Dimitrov T , et al. Oncofetal gene SALL4 in aggressive hepatocellular carcinoma. N Engl J Med. 2013;368:2266–76. 10.1056/NEJMoa1300297 23758232 PMC3781214

[14] Zhang X , Yuan X , Zhu W , Qian H , Xu W . SALL4: an emerging cancer biomarker and target. Cancer Lett. 2015;357:55–62. 10.1016/j.canlet.2014.11.037 25444934

[15] Cheng J , Gao J , Shuai X , Tao K . Oncogenic protein SALL4 and ZNF217 as prognostic indicators in solid cancers: a meta‐analysis of individual studies. Oncotarget. 2016;7:24314–25. 10.18632/oncotarget.8237 27007163 PMC5029703

[16] Nicolè L , Sanavia T , Veronese N , Cappellesso R , Luchini C , Dabrilli P , et al. Oncofetal gene SALL4 and prognosis in cancer: a systematic review with meta‐analysis. Oncotarget. 2017;8:22968–79. 10.18632/oncotarget.14952 28160555 PMC5410278

[17] He J , Zhou M , Chen X , Yue D , Yang L , Qin G , et al. Inhibition of SALL4 reduces tumorigenicity involving epithelial‐mesenchymal transition via Wnt/β‐catenin pathway in esophageal squamous cell carcinoma. J Exp Clin Cancer Res. 2016;35:98. 10.1186/s13046-016-0378-z 27329034 PMC4915037

[18] Zhang X , Zhang P , Shao M , Zang X , Zhang J , Mao F , et al. SALL4 activates TGF‐β/SMAD signaling pathway to induce EMT and promote gastric cancer metastasis. Cancer Manag Res. 2018;10:4459–70. 10.2147/CMAR.S177373 30349378 PMC6188178

[19] Huynh DL , Zhang JJ , Chandimali N , Ghosh M , Gera M , Kim N , et al. SALL4 suppresses reactive oxygen species in pancreatic ductal adenocarcinoma phenotype via FoxM1/Prx III axis. Biochem Biophys Res Commun. 2018;503:2248–54. 10.1016/j.bbrc.2018.06.145 29958885

[20] Bankhead P , Loughrey MB , Fernández JA , Dombrowski Y , McArt DG , Dunne PD , et al. QuPath: open source software for digital pathology image analysis. Sci Rep. 2017;7:16878. 10.1038/s41598-017-17204-5 29203879 PMC5715110

[21] Jacquier A , Syrykh C , Bedgedjian I , Monnien F , Laurent C , Valmary‐Degano S , et al. Immunohistochemistry with anti‐MAL antibody and RNAscope with MAL probes are complementary techniques for diagnosis of primary mediastinal large B‐cell lymphoma. J Clin Pathol. 2020;74:396–9. 10.1136/jclinpath-2020-206747 32839159 PMC8142454

[22] Cancer Genome Atlas Research Network , Cancer Genome Atlas Research Network . Integrated genomic characterization of pancreatic ductal adenocarcinoma. Cancer Cell. 2017;32:185–203.e13. 10.1016/j.ccell.2017.07.007 28810144 PMC5964983

[23] Mayakonda A , Lin D‐C , Assenov Y , Plass C , Koeffler HP . Maftools: efficient and comprehensive analysis of somatic variants in cancer. Genome Res. 2018;28:1747–56. 10.1101/gr.239244.118 30341162 PMC6211645

[24] Ritchie ME , Phipson B , Wu D , Hu Y , Law CW , Shi W , et al. limma powers differential expression analyses for RNA‐sequencing and microarray studies. Nucleic Acids Res. 2015;43:e47. 10.1093/nar/gkv007 25605792 PMC4402510

[25] Neuzillet C , Tijeras‐Raballand A , Ragulan C , Cros J , Patil Y , Martinet M , et al. Inter‐ and intra‐tumoural heterogeneity in cancer‐associated fibroblasts of human pancreatic ductal adenocarcinoma. J Pathol. 2019;248:51–65. 10.1002/path.5224 30575030 PMC6492001

[26] Becht E , Giraldo NA , Lacroix L , Buttard B , Elarouci N , Petitprez F , et al. Estimating the population abundance of tissue‐infiltrating immune and stromal cell populations using gene expression. Genome Biol. 2016;17:218. 10.1186/s13059-016-1070-5 27765066 PMC5073889

[27] Calon A , Espinet E , Palomo‐Ponce S , Tauriello DVF , Iglesias M , Céspedes MV , et al. Dependency of colorectal cancer on a TGF‐β‐driven program in stromal cells for metastasis initiation. Cancer Cell. 2012;22:571–84. 10.1016/j.ccr.2012.08.013 23153532 PMC3512565

[28] Calon A , Lonardo E , Berenguer‐Llergo A , Espinet E , Hernando‐Momblona X , Iglesias M , et al. Stromal gene expression defines poor‐prognosis subtypes in colorectal cancer. Nat Genet. 2015;47:320–9. 10.1038/ng.3225 25706628

[29] Dominguez CX , Muller S , Keerthivasan S , Koeppen H , Hung J , Gierke S , et al. Single‐cell RNA sequencing reveals stromal evolution into LRRC15+ myofibroblasts as a determinant of patient response to cancer immunotherapy. Cancer Discov. 2019;10:232–53. 10.1158/2159-8290.CD-19-0644 31699795

[30] Elyada E , Bolisetty M , Laise P , Flynn WF , Courtois ET , Burkhart RA , et al. Cross‐species single‐cell analysis of pancreatic ductal adenocarcinoma reveals antigen‐presenting cancer‐associated fibroblasts. Cancer Discov. 2019;9:1102–23. 10.1158/2159-8290.CD-19-0094 31197017 PMC6727976

[31] Alshetaiwi H , Pervolarakis N , McIntyre LL , Ma D , Nguyen Q , Rath JA , et al. Defining the emergence of myeloid‐derived suppressor cells in breast cancer using single‐cell transcriptomics. Sci Immunol. 2020;5:eaay6017. 10.1126/sciimmunol.aay6017 32086381 PMC7219211

[32] Satija R , Farrell JA , Gennert D , Schier AF , Regev A . Spatial reconstruction of single‐cell gene expression data. Nat Biotechnol. 2015;33:495–502. 10.1038/nbt.3192 25867923 PMC4430369

[33] Peng J , Sun B‐F , Chen C‐Y , Zhou J‐Y , Chen Y‐S , Chen H , et al. Single‐cell RNA‐seq highlights intra‐tumoral heterogeneity and malignant progression in pancreatic ductal adenocarcinoma. Cell Res. 2019;29:725–38. 10.1038/s41422-019-0195-y 31273297 PMC6796938

[34] Hothorn T , Lausen B . On the exact distribution of maximally selected rank statistics. Comput Stat Data Anal. 2003;43:121–37. 10.1016/S0167-9473(02)00225-6

[35] Tillmanns J , Hoffmann D , Habbaba Y , Schmitto JD , Sedding D , Fraccarollo D , et al. Fibroblast activation protein alpha expression identifies activated fibroblasts after myocardial infarction. J Mol Cell Cardiol. 2015;87:194–203. 10.1016/j.yjmcc.2015.08.016 26319660

[36] Micallef L , Vedrenne N , Billet F , Coulomb B , Darby IA , Desmoulière A . The myofibroblast, multiple origins for major roles in normal and pathological tissue repair. Fibrogenesis Tissue Repair. 2012;5:S5. 10.1186/1755-1536-5-S1-S5 23259712 PMC3368789

[37] Murphy SJ , Hart SN , Lima JF , Kipp BR , Klebig M , Winters JL , et al. Genetic alterations associated with progression from pancreatic intraepithelial neoplasia to invasive pancreatic tumor. Gastroenterology. 2013;145:1098–109.e1. 10.1053/j.gastro.2013.07.049 23912084 PMC3926442

[38] Sodir NM , Kortlever RM , Barthet VJA , Campos T , Pellegrinet L , Kupczak S , et al. MYC instructs and maintains pancreatic adenocarcinoma phenotype. Cancer Discov. 2020;10:588–607. 10.1158/2159-8290.CD-19-0435 31941709

[39] Hung S‐P , Ho JH , Shih Y‐RV , Lo T , Lee OK . Hypoxia promotes proliferation and osteogenic differentiation potentials of human mesenchymal stem cells. J Orthop Res. 2012;30:260–6. 10.1002/jor.21517 21809383

[40] Duarte BDP , Bonatto D . The heat shock protein 47 as a potential biomarker and a therapeutic agent in cancer research. J Cancer Res Clin Oncol. 2018;144:2319–28. 10.1007/s00432-018-2739-9 30128672 PMC11813397

[41] Chang X‐Z , Yu J , Liu H‐Y , Dong R‐H , Cao X‐C . ARK5 is associated with the invasive and metastatic potential of human breast cancer cells. J Cancer Res Clin Oncol. 2012;138:247–54. 10.1007/s00432-011-1102-1 22105900 PMC11824327

[42] Golubkov VS , Prigozhina NL , Zhang Y , Stoletov K , Lewis JD , Schwartz PE , et al. Protein‐tyrosine pseudokinase 7 (PTK7) directs cancer cell motility and metastasis. J Biol Chem. 2014;289:24238–49. 10.1074/jbc.M114.574459 25006253 PMC4148854

[43] Purcell JW , Tanlimco SG , Hickson J , Fox M , Sho M , Durkin L , et al. LRRC15 is a novel mesenchymal protein and stromal target for antibody‐drug conjugates. Cancer Res. 2018;78:4059–72. 10.1158/0008-5472.CAN-18-0327 29764866

[44] Navab R , Strumpf D , To C , Pasko E , Kim KS , Park CJ , et al. Integrin α11β1 regulates cancer stromal stiffness and promotes tumorigenicity and metastasis in non‐small cell lung cancer. Oncogene. 2016;35:1899–908. 10.1038/onc.2015.254 26148229 PMC4833874

[45] Chen D , Bhat‐Nakshatri P , Goswami C , Badve S , Nakshatri H . ANTXR1, a stem cell‐enriched functional biomarker, connects collagen signaling to cancer stem‐like cells and metastasis in breast cancer. Cancer Res. 2013;73:5821–33. 10.1158/0008-5472.CAN-13-1080 23832666 PMC3778138

[46] Bin‐Nun N , Lichtig H , Malyarova A , Levy M , Elias S , Frank D . PTK7 modulates Wnt signaling activity via LRP6. Development. 2014;141:410–21. 10.1242/dev.095984 24353057

[47] Costa A , Kieffer Y , Scholer‐Dahirel A , Pelon F , Bourachot B , Cardon M , et al. Fibroblast heterogeneity and immunosuppressive environment in human breast cancer. Cancer Cell. 2018;33:463–79.e10. 10.1016/j.ccell.2018.01.011 29455927

[48] Kieffer Y , Hocine HR , Gentric G , Pelon F , Bernard C , Bourachot B , et al. Single‐cell analysis reveals fibroblast clusters linked to immunotherapy resistance in cancer. Cancer Discov. 2020;10:1330–51. 10.1158/2159-8290.CD-19-1384 32434947

[49] Ren J , Smid M , Iaria J , Salvatori DCF , van Dam H , Zhu HJ , et al. Cancer‐associated fibroblast‐derived Gremlin 1 promotes breast cancer progression. Breast Cancer Res. 2019;21:109. 10.1186/s13058-019-1194-0 31533776 PMC6751614

[50] Sung P‐J , Rama N , Imbach J , Fiore S , Ducarouge B , Neves D , et al. Cancer‐associated fibroblasts produce Netrin‐1 to control cancer cell plasticity. Cancer Res. 2019;79:3651–61. 10.1158/0008-5472.CAN-18-2952 31088838

[51] Dumartin L , Quemener C , Laklai H , Herbert J , Bicknell R , Bousquet C , et al. Netrin‐1 mediates early events in pancreatic adenocarcinoma progression, acting on tumor and endothelial cells. Gastroenterology. 2010;138:1595–1606.e1–8. 10.1053/j.gastro.2009.12.061 20080097

[52] Wang L , Zhi X , Zhu Y , Zhang Q , Wang W , Li Z , et al. MUC4‐promoted neural invasion is mediated by the axon guidance factor Netrin‐1 in PDAC. Oncotarget. 2015;6:33805–22. 10.18632/oncotarget.5668 26393880 PMC4741804

[53] Kesh K , Garrido VT , Dosch A , Durden B , Gupta VK , Sharma NS , et al. Stroma secreted IL6 selects for “stem‐like” population and alters pancreatic tumor microenvironment by reprogramming metabolic pathways. Cell Death Dis. 2020;11:967. 10.1038/s41419-020-03168-4 33177492 PMC7658205

[54] Ozmadenci D , Féraud O , Markossian S , Kress E , Ducarouge B , Gibert B , et al. Netrin‐1 regulates somatic cell reprogramming and pluripotency maintenance. Nat Commun. 2015;6:7398. 10.1038/ncomms8398 26154507 PMC4510695

[55] Peran I , Madhavan S , Byers SW , McCoy MD . Curation of the pancreatic ductal adenocarcinoma subset of the cancer genome atlas is essential for accurate conclusions about survival‐related molecular mechanisms. Clin Cancer Res. 2018;24:3813–9. 10.1158/1078-0432.CCR-18-0290 29739787

[56] Nicolle R , Raffenne J , Paradis V , Couvelard A , de Reynies A , Blum Y , et al. Prognostic biomarkers in pancreatic cancer: avoiding errata when using the TCGA dataset. Cancer. 2019;11:126. 10.3390/cancers11010126 PMC6357157 30669703

[57] Knudsen E , Vail P , Balaji U , Ngo H , Botros IW , Makarov V , et al. Stratification of pancreatic ductal adenocarcinoma: combinatorial genetic, stromal, and immunological markers. Clin Cancer Res. 2017;23:4429–40. 10.1158/1078-0432.CCR-17-0162 28348045 PMC5951386

[58] Mahajan UM , Langhoff E , Goni E , Costello E , Greenhalf W , Halloran C , et al. Immune cell and stromal signature associated with progression‐free survival of patients with resected pancreatic ductal adenocarcinoma. Gastroenterology. 2018;155:1625–39.e2. 10.1053/j.gastro.2018.08.009 30092175

[59] Ogawa Y , Masugi Y , Abe T , Yamazaki K , Ueno A , Fujii‐Nishimura Y , et al. Three distinct stroma types in human pancreatic cancer identified by image analysis of fibroblast subpopulations and collagen. Clin Cancer Res. 2021;27:107–19. 10.1158/1078-0432.CCR-20-2298 33046515

[60] Vienot A , Pallandre J‐R , Renaude E , Viot J , Bouard A , Spehner L , et al. Chemokine switch regulated by TGF‐β1 in cancer‐associated fibroblast subsets determines the efficacy of chemo‐immunotherapy. Oncoimmunology. 2022;11:2144669. 10.1080/2162402X.2022.2144669 36387055 PMC9662195

[61] Feldmann K , Maurer C , Peschke K , Teller S , Schuck K , Steiger K , et al. Mesenchymal plasticity regulated by Prrx1 drives aggressive pancreatic cancer biology. Gastroenterology. 2021;160:346–61.e24. 10.1053/j.gastro.2020.09.010 33007300

[62] Maitra A , Iacobuzio‐Donahue C , Rahman A , Sohn TA , Argani P , Meyer R , et al. Immunohistochemical validation of a novel epithelial and a novel stromal marker of pancreatic ductal adenocarcinoma identified by global expression microarrays: sea urchin fascin homolog and heat shock protein 47. Am J Clin Pathol. 2002;118:52–9. 10.1309/3PAM-P5WL-2LV0-R4EG 12109856

[63] Yoneda A , Minomi K , Tamura Y . Heat shock protein 47 confers chemoresistance on pancreatic cancer cells by interacting with calreticulin and IRE1α. Cancer Sci. 2021;112:2803–20. 10.1111/cas.14976 34109710 PMC8253297

