# Supplementary material for: SALL4‐related gene signature defines a specific stromal subset of pancreatic ductal adenocarcinoma with poor prognostic features
Source: Mol Oncol. 2023 Jan 21;17(7):1356–78. doi: 10.1002/1878-0261.13370 (PMC10323888; doi:10.1002/1878-0261.13370)
Supplement: Supplementary file 1 — Fig. S1. Determination of the prognostic threshold to split groups according to SALL4 expression in pancreatic ductal adenocarcinoma. Fig. S2. The landscape of mutations in pancreatic ductal adenocarcinoma according to SALL4 expression. Fig. S3. Validation of the phenotype of SALL4 expression in pancreatic ductal adenocarcinoma. Fig. S4. Validation of the SALL4 signature. Fig. S5. Molecular pathways and functionality of SALL4‐expressing fibroblasts. Fig. S6. Evaluation of stromal signature in immunochemistry. Table S1. Immunohistochemistry antibodies. Table S2. Characteristics of the selected publicly datasets. Table S3. Univariate analyses of different stemness‐related genes for overall survival in the ICGC cohort. Table S4. Patients' characteristics according to SALL4 expression (using the upper tertile cut‐off) for 259 patients with localized pancreatic carcinoma in the ICGC cohort. Table S5. Mutational profile according to SALL4 expression for patients with localized pancreatic carcinoma in the GSE85916 and TCGA cohorts. Table S6. List of correlated genes with SALL4 expression in the ICGC, GSE85916, and TCGA cohorts Table S7. Univariate analyses of different pathways for overall survival in the ICGC cohort. [file MOL2-17-1356-s001.pdf]

SALL4-related gene signature defines a specific stromal subset of pancreatic ductal adenocarcinoma with poor prognostic features

SUPPLEMENTARY FIGURES

Figure S1. Determination of the prognostic threshold to split groups according to *SALL4* expression in pancreatic ductal adenocarcinoma. A) Histograms of the distribution of *SALL4* expression in the ICGC and GSE85916 cohorts. The SALL4 gene expression assumed a normal distribution and showed no evident cohort-bias clustering. **B)** Kaplan-Meier curves of overall survival for 257 patients with localized pancreatic carcinoma in the ICGC cohort. Groups were split by high (red) or low (blue) levels of *SALL4* expression: median, tertile, quartile, and upper tertile cutoffs.

Figure S2. The landscape of mutations in pancreatic ductal adenocarcinoma according to *SALL4* expression. A) Color-coded matrix of individual mutations of the genetic and top most mutated genes and for *SALL4* high (left) and *SALL4* low (right) groups, in the ICGC cohort (n=224). **B)** Tumor mutation burden (TMB) for *SALL4* high and *SALL4* low groups (median TMB: 1.06 versus 1.03 mutation/Mb, respectively) in the ICGC cohort compared to different cancer types from TCGA.

Figure S3. Validation of the phenotype of *SALL4* expression in pancreatic ductal adenocarcinoma. A) Boxplots and barplots comparing the distribution of the *SALL4* expression in subtypes from molecular classifications (Moffitt *et al* and Puleo *et al*) in the TCGA cohort. **B)** Boxplots comparing the distribution of the fibroblast signature to the *SALL4* expression in TCGA and GSE85916 cohorts. **C)** Boxplots comparing the distribution of the stem cell signature to the *SALL4* expression in the TCGA and GSE85916 cohorts. **D)** Boxplots and barplots comparing the distribution of the *SALL4* expression in different subtypes from CAF classification (Neuzillet *et al*) in the ICGC cohort. **E)** Boxplots comparing the distribution of the F-TBRS and TGFB CAF signatures to the *SALL4* expression in TCGA and GSE85916 cohorts. Boxplots show the median and interquartile range. Medians were compared using Student's t-tests.

Figure S4. Validation of the *SALL4* signature. A) Heatmaps visualizing the relative average expression of indicated genes (rows) according to *SALL4* expression for patients with localized pancreatic carcinoma in the TCGA and GSE85916 cohorts. **B)** Boxplots comparing the distribution of the immune MCP-counter signatures to the *SALL4* expression in the ICGC cohort. Boxplots show the median and interquartile range. Medians were compared using Student's t-tests. **C)** Correlation matrix showing Pearson's correlation coefficients from comparisons between genes of the *SALL4* signature and MCP-counter signatures in the TCGA and GSE85916 cohorts. **D)** Signature-by signature correlation matrix visualizing the pairwise Pearson's correlation coefficients in the ICGC, TCGA and GSE85916 cohorts.

Figure S5. Molecular pathways and functionality of SALL4-expressing fibroblasts. A) Protein expression of SALL4 isoforms (SALL4A: 165 kDa; SALLB: 95 kDa) evaluated in the same culture condition, with Actin used as an internal control in $MRC5^{ctrl}$ and $MRC5^{SALL4}$ using lentivirus-mediated

transduction for one of a representative experiment. **B)** Number of colony formation with Colo320 ± MRC5 with or without SALL4 transduction using soft agar colony formation assay (Data are displayed as mean ± standard deviation; scale bars, 200 µm). One representative experiment is shown (n=3). **C)** Representative image of a spheroid with Panc-1 and MRC5 with or without SALL4 transduction (Day-4; scale bars, 200 µm).

Figure S6. Evaluation of stromal signature in immunochemistry. A) Heatmaps visualizing the relative pairwise Pearson's correlation coefficients between the 24 genes associated with *SALL4* expression in the ICGC, TCGA, and GSE85916 cohorts. **B)** Kaplan-Meier curves of overall survival according to 7-genes signature expression (best cutoff), compared using the log-rank test, in ICGC and GSE85916 cohorts. **C)** Heatmaps visualizing the relative average expression of indicated genes (rows) according to patients' survival from 22 localized pancreatic carcinomas from Nanostring analysis (top). Boxplots comparing the distribution of the 24-gene and 7-gene signatures to patients' survival in the same cohort (bottom). Boxplots show the median and interquartile range. Medians were compared using Student's t-tests. **D)** Semi-quantitative scale used to evaluate PTK7 in stromal cells.

Figure S1

A

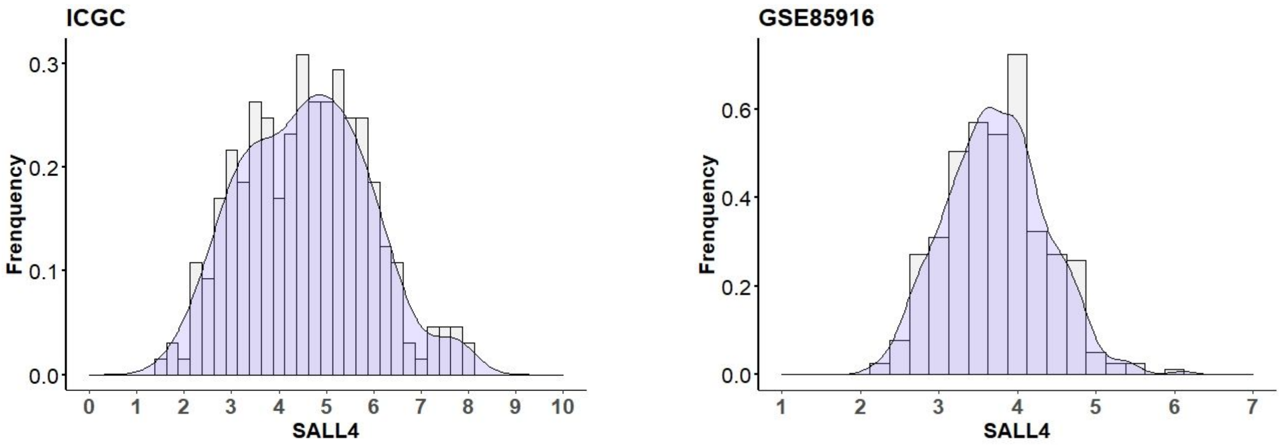
ICGC
Frenquency
SALL4
GSE85916
Frenquency
SALL4

B

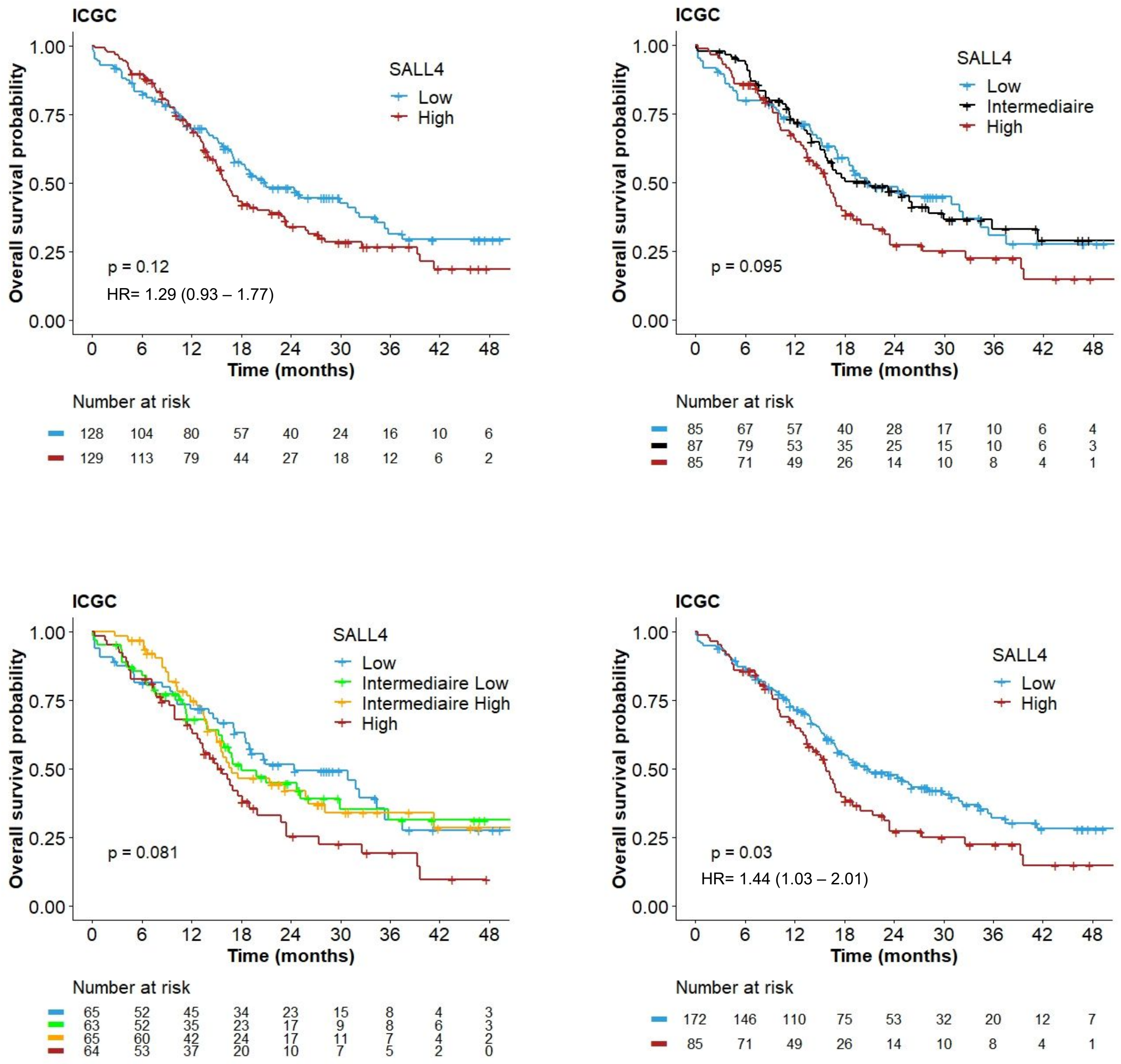
ICGC
SALL4
Low
High
p = 0.12
HR= 1.29 (0.93 – 1.77)
Overall survival probability
Time (months)
Number at risk
128 104 80 57 40 24 16 10 6
129 113 79 44 27 18 12 6 2
ICGC
SALL4
Low
Intermediaire
High
p = 0.095
Overall survival probability
Time (months)
Number at risk
85 67 57 40 28 17 10 6 4
87 79 53 35 25 15 10 6 3
85 71 49 26 14 10 8 4 1
ICGC
SALL4
Low
Intermediaire Low
Intermediaire High
High
p = 0.081
Overall survival probability
Time (months)
Number at risk
65 52 45 34 23 15 8 4 3
63 52 35 23 17 9 8 6 3
65 60 42 24 17 11 7 4 2
64 53 37 20 10 7 5 2 0
ICGC
SALL4
Low
High
p = 0.03
HR= 1.44 (1.03 – 2.01)
Overall survival probability
Time (months)
Number at risk
172 146 110 75 53 32 20 12 7
85 71 49 26 14 10 8 4 1

Figure S2

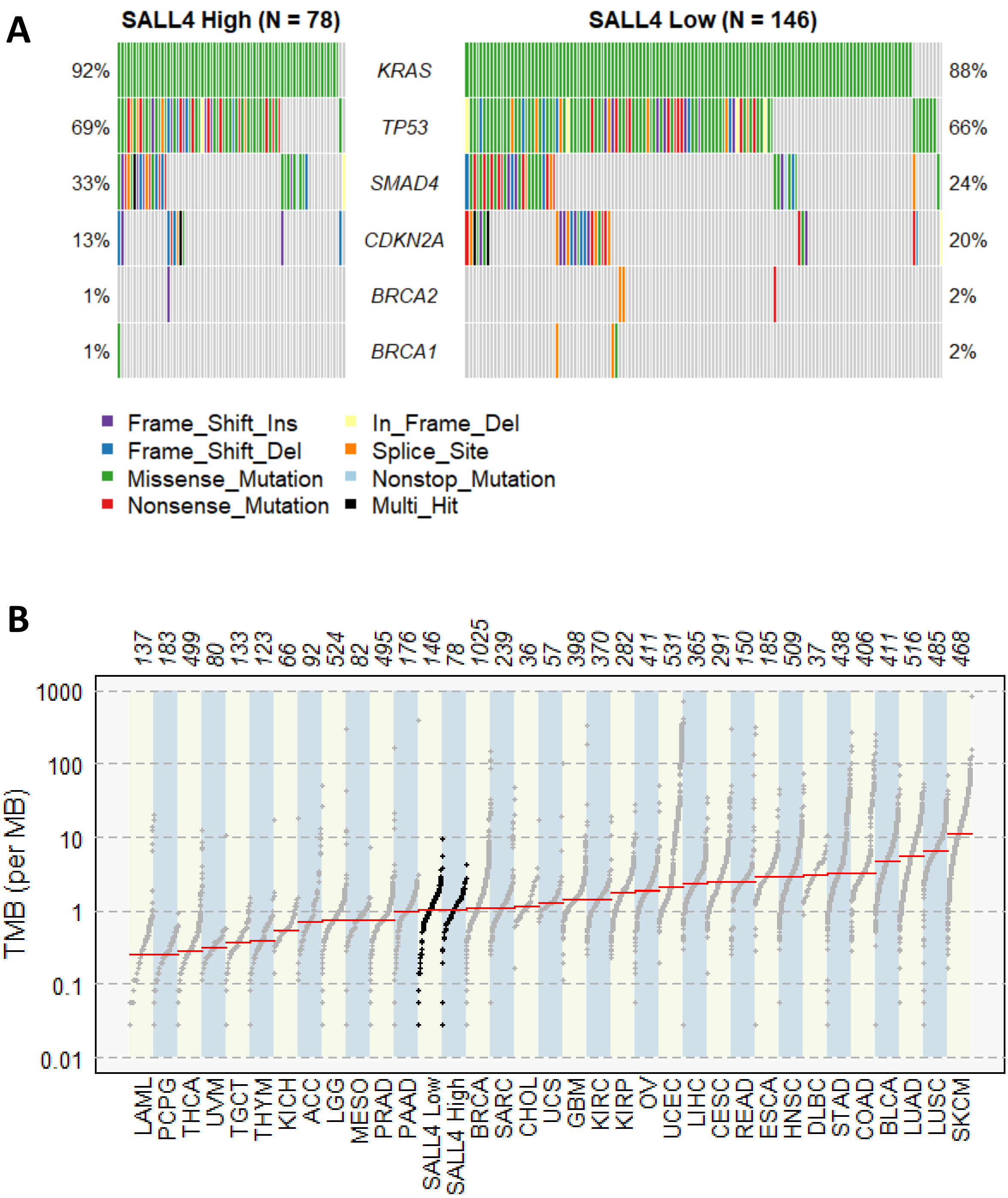
A
SALL4 High (N = 78)
SALL4 Low (N = 146)
92%
KRAS
88%
69%
TP53
66%
33%
SMAD4
24%
13%
CDKN2A
20%
1%
BRCA2
2%
1%
BRCA1
2%
Frame_Shift_Ins
Frame_Shift_Del
Missense_Mutation
Nonsense_Mutation
In_Frame_Del
Splice_Site
Nonstop_Mutation
Multi_Hit
B
137 183 499 80 133 123 66 92 524 82 495 176 146 78 1025 239 36 57 398 370 282 411 531 365 291 150 185 509 37 438 406 411 516 485 468
1000
100
10
1
0.1
0.01
TMB (per MB)
LAML PCPG THCA UVM TGCT THYM KICH ACC LGG MESO PRAD PAAD SALL4 Low SALL4 High BRCA SARC CHOL UCS GBM KIRC KIRP OV UCEC LIHC CESC READ ESCA HNSC DLBC STAD COAD BLCA LUAD LUSC SKCM

Figure S3

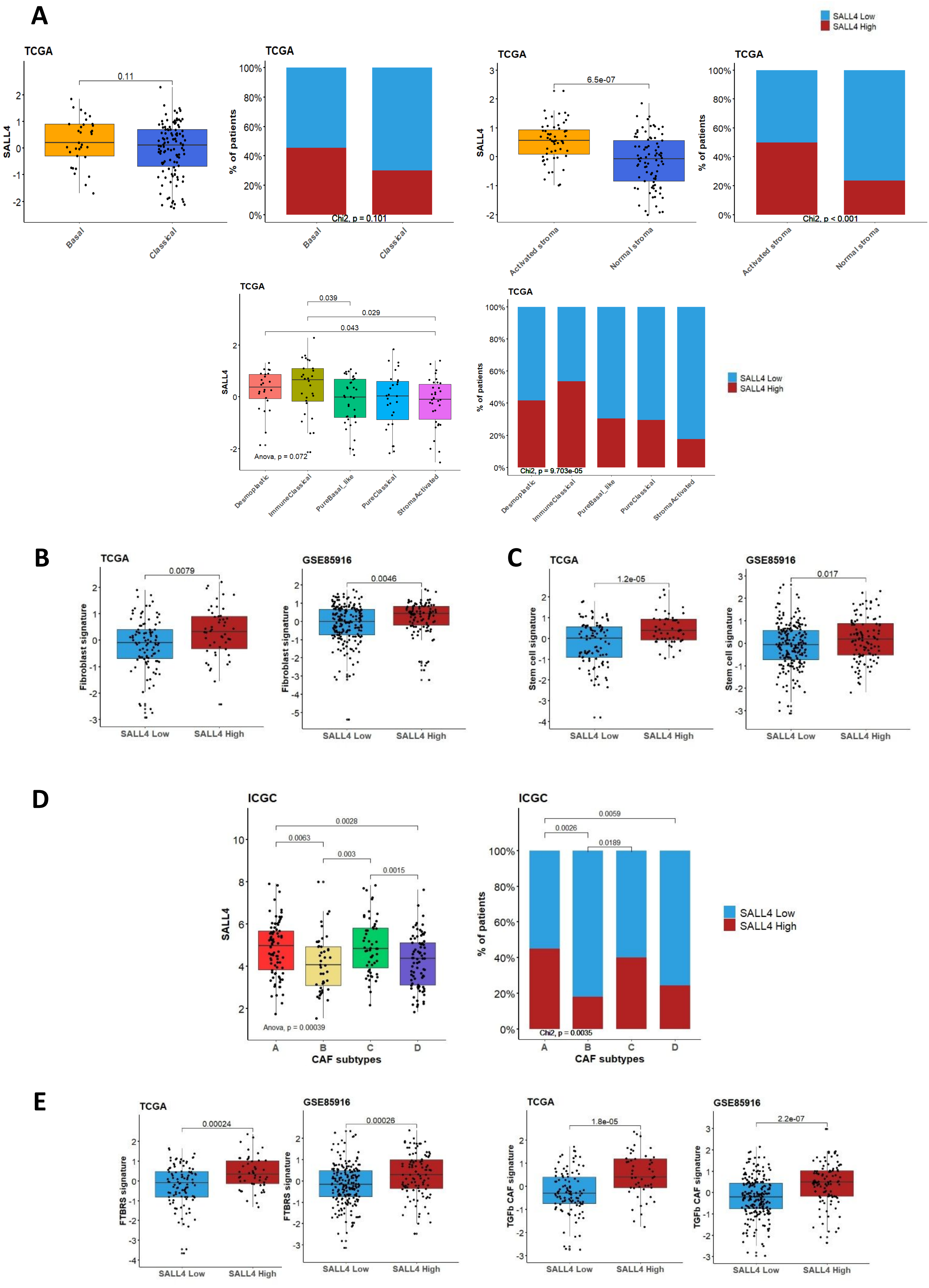

A
SALL4 Low
SALL4 High
TCGA
0.11
SALL4
Basal
Classical
% of patients
Chi2, p = 0.101
6.5e-07
Activated stroma
Normal stroma
Chi2, p < 0.001
0.039
0.029
0.043
Anova, p = 0.072
Desmoplastic
ImmuneClassical
PureBasal_like
PureClassical
StromaActivated
Chi2, p = 9.703e-05
B
0.0079
Fibroblast signature
GSE85916
0.0046
C
1.2e-05
Stem cell signature
0.017
D
ICGC
0.0028
0.0063
0.003
0.0015
Anova, p = 0.00039
CAF subtypes
0.0059
0.0026
0.0189
Chi2, p = 0.0035
E
0.00024
FTBRS signature
0.00026
1.8e-05
TGFb CAF signature
2.2e-07

Figure S4

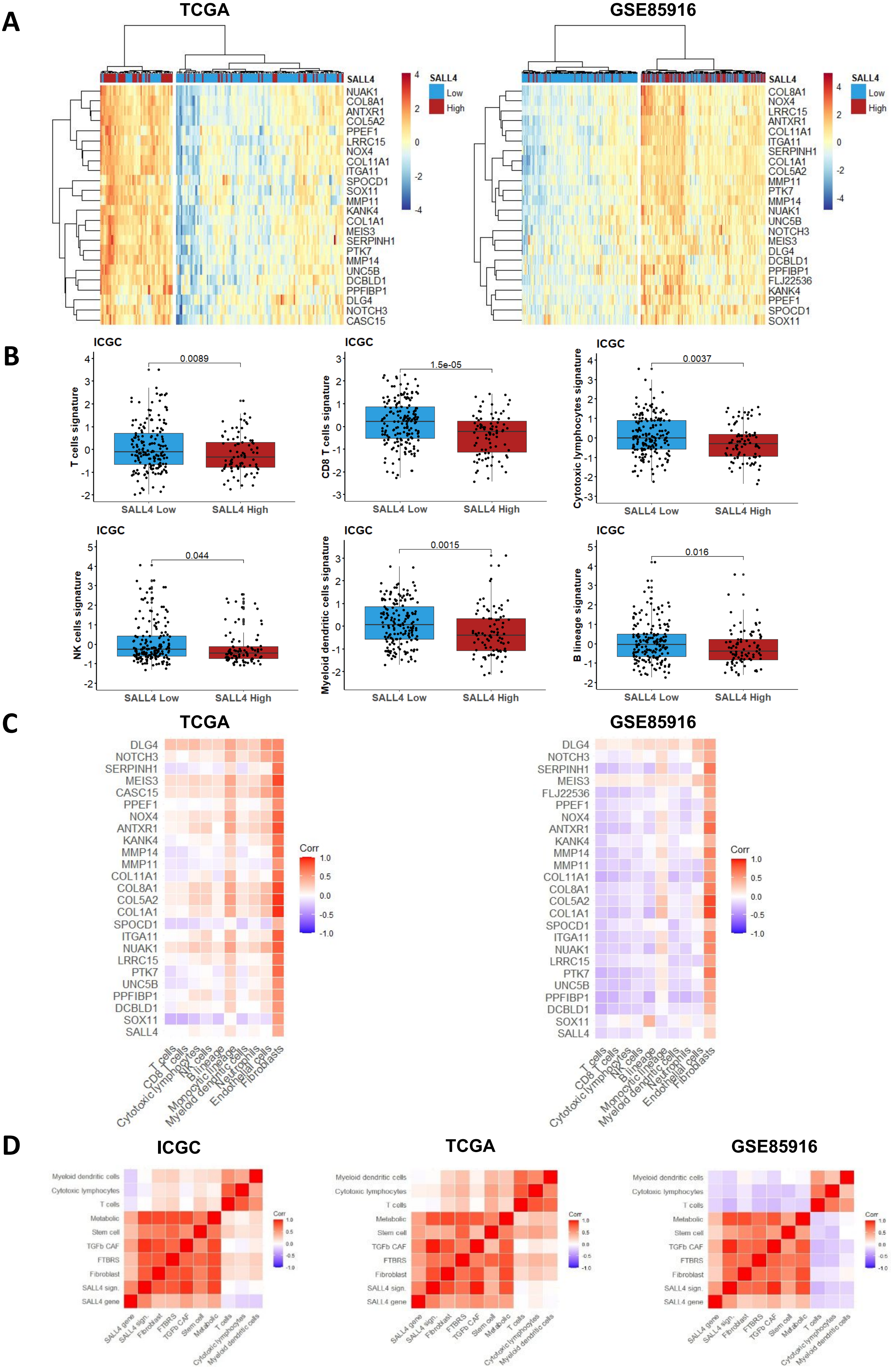
A
TCGA
GSE85916
SALL4
Low
High
NUAK1
COL8A1
ANTXR1
COL5A2
PPEF1
LRRC15
NOX4
COL11A1
ITGA11
SPOCD1
SOX11
MMP11
KANK4
COL1A1
MEIS3
SERPINH1
PTK7
MMP14
UNC5B
DCBLD1
PPFIBP1
DLG4
NOTCH3
CASC15
FLJ22536
B
ICGC
T cells signature
CD8 T cells signature
Cytotoxic lymphocytes signature
NK cells signature
Myeloid dendritic cells signature
B lineage signature
SALL4 Low
SALL4 High
0.0089
1.5e-05
0.0037
0.044
0.0015
0.016
C
Corr
T cells
CD8 T cells
Cytotoxic lymphocytes
NK cells
B lineage
Monocytic lineage
Myeloid dendritic cells
Neutrophils
Endothelial cells
Fibroblasts
D
ICGC
TCGA
GSE85916
Myeloid dendritic cells
Cytotoxic lymphocytes
T cells
Metabolic
Stem cell
TGFb CAF
FTBRS
Fibroblast
SALL4 sign.
SALL4 gene

Figure S5

A

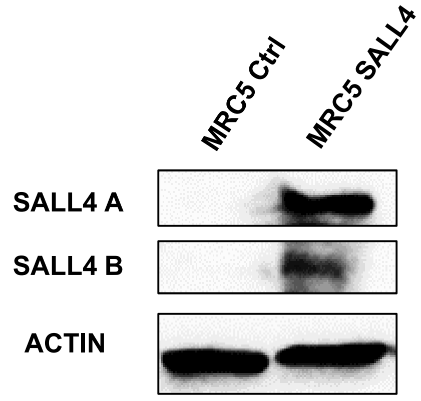

B

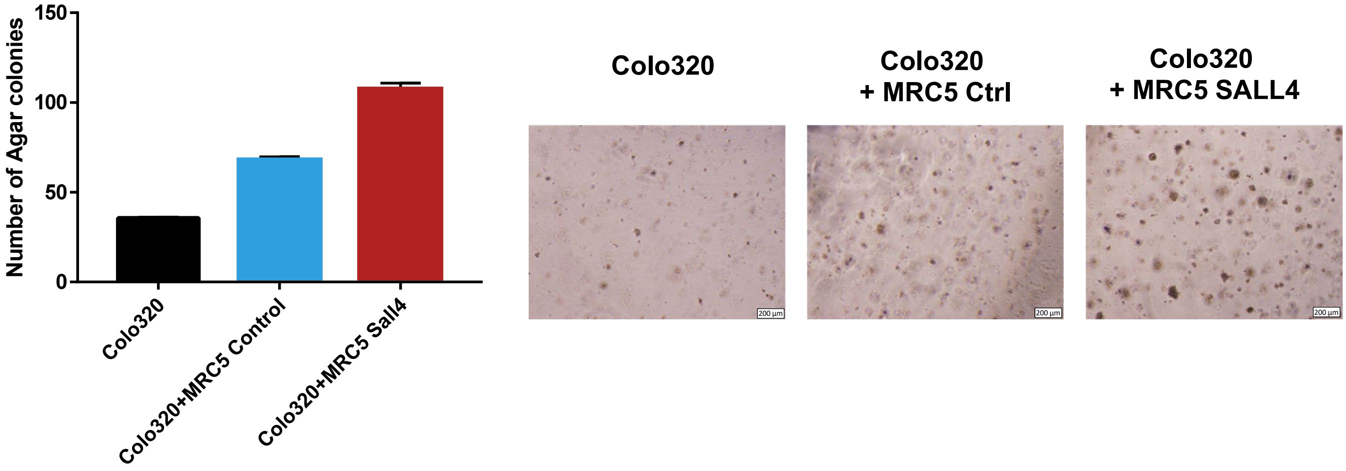

C

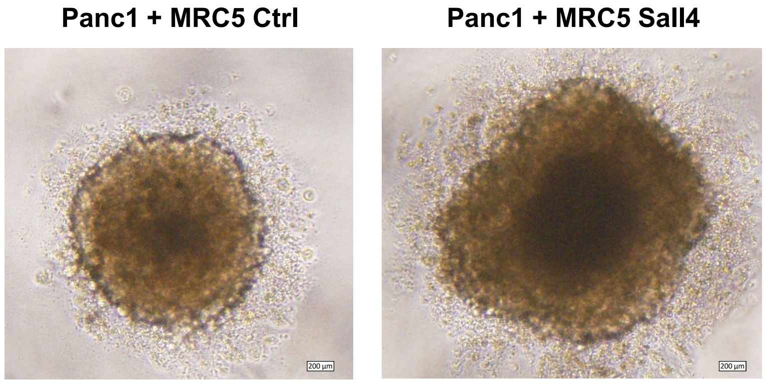

Figure S6

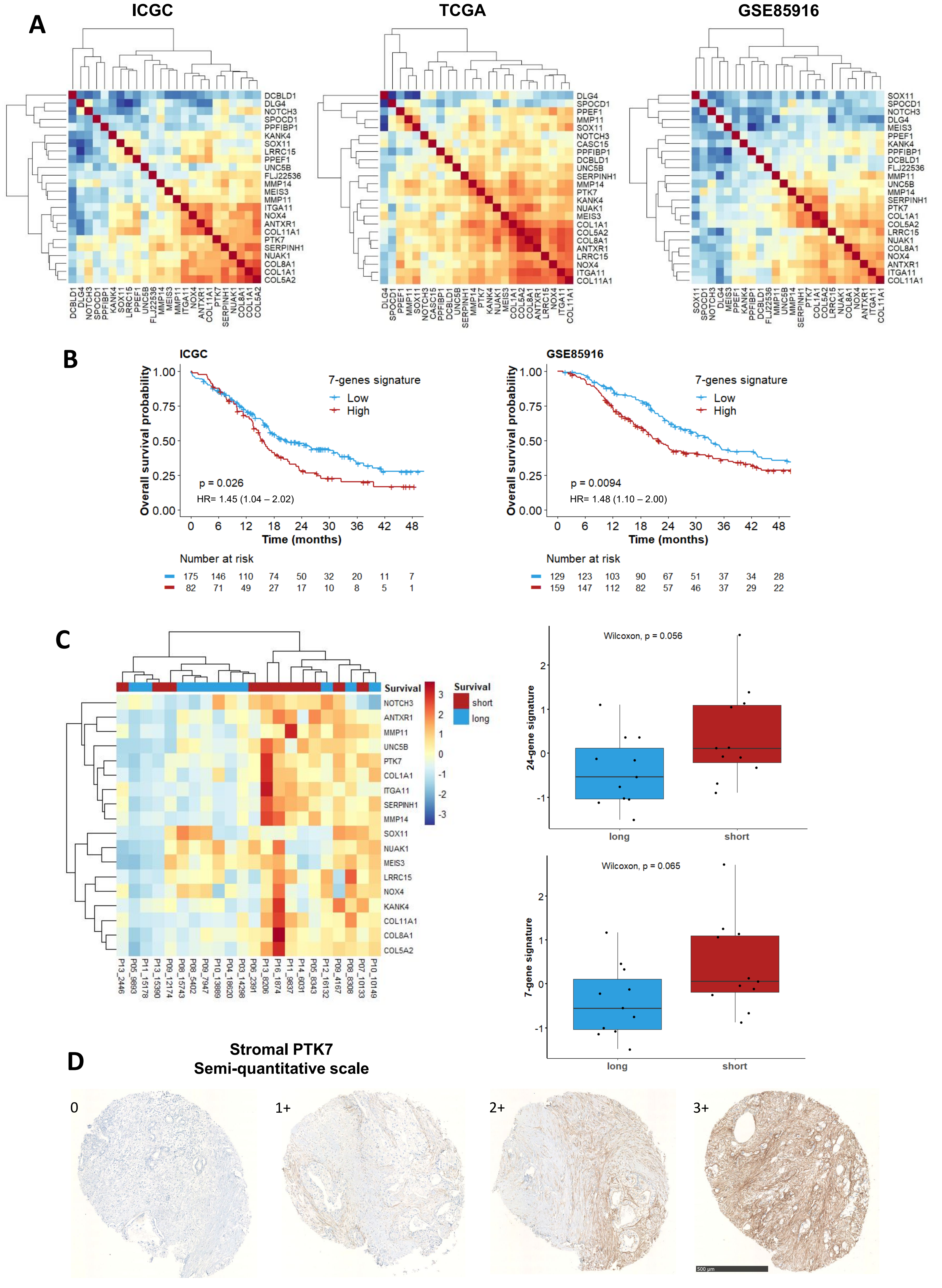
A
ICGC
TCGA
GSE85916
DCBLD1
DLG4
NOTCH3
SPOCD1
PPFIBP1
KANK4
SOX11
LRRC15
PPEF1
UNC5B
FLJ22536
MMP14
MEIS3
MMP11
ITGA11
NOX4
ANTXR1
COL11A1
PTK7
SERPINH1
NUAK1
COL8A1
COL1A1
COL5A2
CASC15
COL5A2
B
ICGC
GSE85916
7-genes signature
Low
High
Overall survival probability
p = 0.026
HR= 1.45 (1.04 – 2.02)
p = 0.0094
HR= 1.48 (1.10 – 2.00)
Time (months)
Number at risk
175 146 110 74 50 32 20 11 7
82 71 49 27 17 10 8 5 1
129 123 103 90 67 51 37 34 28
159 147 112 82 57 46 37 29 22
C
Survival
short
long
Wilcoxon, p = 0.056
24-gene signature
Wilcoxon, p = 0.065
7-gene signature
long
short
D
Stromal PTK7
Semi-quantitative scale
0
1+
2+
3+
500 µm

SUPPLEMENTARY TABLES

Table S1. Immunohistochemistry antibodies.

Antibody	Reference	Species	Clone	Source	Dilution
PTK7	#25618	Rabbit	D2Z1N	Cell Signaling	1/100
SERPINH1	sc-5293	Mouse	G-12	Santa Cruz	1/500
LRRC15	Ab150376	Rabbit	EPR8188(2)	Abcam	1/300
αSMA	M085129-2	Mouse	1A4	Agilent	1/300
FAP	Ab207178	Rabbit	Ab207178	Abcam	1/250
CD3	A045201	Rabbit	Polyclonal	Agilent	1/200

Table S2. Characteristics of the selected publicly datasets.

Cohort	Dataset	Platform	Technology	Transcriptomic data	Localized PDAC samples	Survival data available	SALL4 probe	References
Development	ICGC	NA	Illumina	Microarray	259	257	ILMN_1695687	Bailey *et al*, Nature 2016 [2]
Validation	TCGA	NA	Illumina	RNA-seq	145	145	NA	Cancer Genome Atlas, Cancer Cell 2017 [22]
	GSE85916	GPL13667	Affymetrix	microarray	309	288	11728065_a_at	Puleo *et al*, Gastroenterology 2018 [5]

Abbreviations: ICGC: International Cancer Genome Consortium; PDAC: pancreatic ductal adenocarcinoma; RNA-seq: RNA-sequencing, TCGA: The Cancer Genome Atlas, NA: not available.

Table S3. Univariate analyses of different stemness-related genes for overall survival in the ICGC cohort.

	Univariate analysis				Multivariate analysis	
**Parameters**	**No. of patients**	**No. of events**	**Hazard ratio (95%CI)**	**P-value***	**Hazard ratio (95%CI)**	**P-value***
**SOX2**						
Low	147	78	1		1	
High	112	76	1.45 (1.06 – 2.00)	0.02	1.50 (1.09 – 2.07)	0.01
**OCT4/POU5F1**						
Low	136	88	1			
High	123	66	0.78 (0.58 – 1.10)	0.17		
**NANOG**						
Low	51	25	1			
High	208	129	1.34 (0.87 – 2.06)	0.18		
**KLF4**						
Low	50	33	1			
High	209	121	0.69 (0.47 – 1.02)	0.06		
**MYC**						
Low	51	32	1			
High	208	122	0.70 (0.47 – 1.34)	0.07		
**SALL4**						
Low	158	88	1		1	
High	101	66	1.55 (1.12 – 2.15)	0.007	1.60 (1.16 – 2.22)	0.005

*Cox-proportional-hazard models used to estimate the association of the parameters with overall survival. Values of $P<0.05$ were considered statistically significant and all tests were two-sided.

Table S4. **Patients' characteristics according to *SALL4* expression (using the upper tertile cut-off) for 259 patients with localized pancreatic carcinoma in the ICGC cohort.**

Characteristics	$SALL4^{low}$ (N = 173)	$SALL4^{high}$ (N = 86)	P-value
**Demographic parameters**			
**Age**, median [IQR], years	30.8 [25.0 – 38.0]	29.7 [24.0 – 38.8]	0.42
**Sex**, No. (%)			0.14
Male	90 (52.0)	53 (61.6)	
Female	83 (48.0)	33 (38.4)	
**Ethnicity**, No. (%)			0.54
White	128 (92.1)	69 (89.6)	
Asian, black or african american	11 (7.9)	8 (10.4)	
Missing	34	9	
**Smoking status**, No. (%)			0.90
Never Smoked	58 (44.3)	30 (42.3)	
Stopped Smoking	20 (15.3)	10 (14.1)	
Still Smoking	53 (40.4)	31 (43.7)	
Missing	42	15	
**Pathologic parameters**			
**Histology**, No. (%)			0.64
Adenocarcinoma	118 (80.8)	65 (83.3)	
Other histology	28 (19.2)	13 (16.7)	
Missing	27	8	
**Stage**, No. (%)			0.04
I	14 (9.6)	4 (5.1)	
II	132 (90.4)	71 (91.0)	
III	0 (0.0)	3 (3.9)	
Missing	27	8	
**Primary tumor site**, No. (%)			0.51
Head	123 (84.2)	63 (80.8)	
Body and/or tail	23 (15.8)	15 (19.2)	
Missing	27	8	
**Tumor grade**, No. (%)			0.90
G1	7 (4.9)	3 (3.9)	
G2	89 (61.8)	47 (61.0)	
G3	46 (31.9)	25 (32.5)	
G4	2 (1.4)	2 (2.6)	
Missing	29	9	

*χ2 tests or Fisher's exact tests used to compare proportions, and Student's t-tests used to compare continuous variables between $SALL4^{high}$ and $SALL4^{low}$. Values of P<0.05 were considered statistically significant and all tests were two-sided.

Table S5. **Mutational profile according to *SALL4* expression for patients with localized pancreatic carcinoma in the GSE85916 and TCGA cohorts.**

	GSE85916 cohort			TCGA cohort		
Characteristics	$SALL4^{low}$ (N = 206)	$SALL4^{high}$ (N = 103)	P-value	$SALL4^{low}$ (N = 99)	$SALL4^{high}$ (N = 50)	P-value
**KRAS status**, No. (%)			0.73			0.73
Wild type	23 (12.3)	10 (10.9)		6 (6.1)	4 (8.0)	
Mutated	164 (87.7)	82 (89.1)		93 (93.9)	46 (92.0)	
Missing	19	11		0	0	
**TP53 status**, No. (%)			0.92			
Wild type	58 (31.0)	28 (30.4)		-	-	
Mutated	129 (69.0)	64 (69.6)		-	-	
Missing	19	11				
**CDKN2A status**, No. (%)			0.45			0.12
Wild type	159 (85.0)	75 (81.5)		63 (63.6)	22 (44.0)	
Mutated	28 (15.0)	17 (18.5)		15 (15.2)	11 (22.0)	
Deleted	-	-		18 (18.2)	15 (30.0)	
Methylated	-	-		3 (3.0)	2 (4.0)	
Missing	19	11		0	0	

*χ2 tests or Fisher's exact tests used to compare proportions between $SALL4^{high}$ and $SALL4^{low}$. Values of P<0.05 were considered statistically significant and all tests were two-sided.

<u>Table S6</u>. List of correlated genes with SALL4 expression in the ICGC, GSE85916, and TCGA cohorts

ICGC				
**Gene symbol**	**Correlation coefficient**	**p-value**	**p-value adjust**	**Gene description**
PTK7	0,564162349	3,63E-23	5,68E-19	Protein Tyrosine Kinase 7
GPX8	0,538922072	6,51E-21	5,86E-17	Glutathione Peroxidase 8
NUAK1	0,538203225	7,50E-21	5,86E-17	NUAK Family Kinase 1
PPFIBP1	0,520377904	2,25E-19	1,01E-15	PPFIA Binding Protein 1
DRD4	0,515496759	5,52E-19	2,16E-15	Dopamine Receptor D4
MGC24103	0,508884625	1,82E-18	5,88E-15	MGC24103
COL8A1	0,507631921	2,27E-18	6,46E-15	Collagen Type VIII Alpha 1 Chain
LOC100131139	0,504298201	4,10E-18	1,07E-14	Similar to double homeobox A
CHPF	0,503688575	4,56E-18	1,10E-14	Chondroitin Polymerizing Factor
LRRFIP1	0,50185281	6,28E-18	1,31E-14	LRR Binding FLII Interacting Protein 1
DCBLD1	0,498733799	1,08E-17	2,11E-14	Discoidin, CUB And LCCL Domain Containing 1
EDNRA	0,497770864	1,27E-17	2,35E-14	Endothelin Receptor Type A
FKBP14	0,495374592	1,92E-17	3,16E-14	FKBP Prolyl Isomerase 14
TAOK1	0,492210165	3,29E-17	4,68E-14	TAO Kinase 1
AEBP1	0,489673837	5,04E-17	6,30E-14	AE Binding Protein 1
MXRA5	0,482696883	1,60E-16	1,85E-13	Matrix Remodeling Associated 5
COL11A1	0,478846375	2,99E-16	3,34E-13	Collagen Type XI Alpha 1 Chain
SOX4	0,477320791	3,83E-16	3,99E-13	SRY-Box Transcription Factor 4
MMP14	0,476077479	4,67E-16	4,72E-13	Matrix Metallopeptidase 14
ZNF827	0,475128651	5,44E-16	5,32E-13	Zinc Finger Protein 827
LOC100134424	0,474263177	6,25E-16	5,92E-13	Similar to Immediate early response 5-like
KIRREL	0,473189859	7,41E-16	6,62E-13	Kirre Like Nephrin Family Adhesion Molecule 1
COL5A2	0,473069999	7,55E-16	6,62E-13	Collagen Type V Alpha 2 Chain
UNC5B	0,473016858	7,61E-16	6,62E-13	Unc-5 Netrin Receptor B
LOC100134134	0,471107551	1,03E-15	8,71E-13	Similar to peroxidasin homolog
VGLL4	0,466628067	2,08E-15	1,62E-12	Vestigial Like Family Member 4
LRRC15	0,465431431	2,50E-15	1,86E-12	Leucine Rich Repeat Containing 15
KLHL28	0,462406949	3,98E-15	2,90E-12	Kelch Like Family Member 28
IER5L	0,462087618	4,18E-15	2,97E-12	Immediate Early Response 5 Like
ITGA11	0,460015643	5,74E-15	3,99E-12	Integrin Subunit Alpha 11
N4BP2	0,456728232	9,43E-15	6,42E-12	NEDD4 Binding Protein 2
COL1A1	0,456041502	1,05E-14	6,75E-12	Collagen Type I Alpha 1 Chain
DCHS1	0,455967687	1,06E-14	6,75E-12	Dachsous Cadherin-Related 1
ZNF532	0,454214543	1,37E-14	8,12E-12	Zinc Finger Protein 532
LOC284297	0,453254601	1,59E-14	8,86E-12	Scavenger receptor cysteine rich family member with 5 domains
NOTCH3	0,451840517	1,96E-14	1,03E-11	Notch Receptor 3
FAM63B	0,451806765	1,97E-14	1,03E-11	MINDY Lysine 48 Deubiquitinase 2
CAMSAP1L1	0,451258382	2,13E-14	1,09E-11	Calmodulin Regulated Spectrin Associated Protein Family Member 2
LOC399900	0,451041582	2,20E-14	1,11E-11	Uncharacterized LOC399900
OSBPL5	0,449671522	2,69E-14	1,34E-11	Oxysterol Binding Protein Like 5
LPAR4	0,449206801	2,88E-14	1,39E-11	Lysophosphatidic Acid Receptor 4
CHD8	0,447860473	3,51E-14	1,64E-11	Chromodomain Helicase DNA Binding Protein 8
DENR	0,445929771	4,65E-14	2,11E-11	Density Regulated Re-Initiation And Release Factor

ZNF669	0,445212838	5,16E-14	2,31E-11	Zinc Finger Protein 669
THBS2	0,44492738	5,38E-14	2,37E-11	Thrombospondin 2
KIAA1217	0,444243317	5,94E-14	2,55E-11	KIAA1217
FLJ22536	0,443253953	6,85E-14	2,84E-11	Cancer Susceptibility 15
TMEM158	0,442537054	7,60E-14	3,05E-11	Transmembrane Protein 158
LOC100131541	0,440644984	9,96E-14	3,75E-11	uncharacterized LOC100131541
ZNF773	0,440400938	1,03E-13	3,84E-11	Zinc Finger Protein 773
C14ORF82	0,440321596	1,04E-13	3,84E-11	FRMD6 Antisense RNA 1
CROCC	0,43972745	1,13E-13	4,04E-11	Ciliary Rootlet Coiled-Coil, Rootletin
RNY3	0,439538463	1,17E-13	4,07E-11	RNA, Ro60-Associated Y3
LEPREL2	0,437204509	1,62E-13	5,40E-11	Prolyl 3-Hydroxylase 3
NUCKS1	0,435986258	1,93E-13	6,28E-11	Nuclear Casein Kinase And Cyclin Dependent Kinase Substrate 1
FCAR	0,43557624	2,04E-13	6,58E-11	Fc Fragment Of IgA Receptor
LOC100129539	0,435511388	2,06E-13	6,58E-11	Hypothetical LOC100129539
LOC440345	0,435111938	2,18E-13	6,88E-11	Nuclear pore complex interacting protein family member B4
EIF2C2	0,434668959	2,32E-13	7,11E-11	Argonaute RISC Catalytic Component 2
HTRA1	0,434085955	2,51E-13	7,56E-11	HtrA Serine Peptidase 1
C1ORF152	0,434002484	2,54E-13	7,58E-11	Profilin 1 Pseudogene 2
WDFY1	0,433701906	2,65E-13	7,83E-11	WD Repeat And FYVE Domain Containing 1
NOX4	0,433228079	2,83E-13	8,29E-11	NADPH Oxidase 4
MAR6	0,432568851	3,11E-13	9,00E-11	Retrotransposon Gag Like 6
LEPRE1	0,430966989	3,88E-13	1,08E-10	Prolyl 3-Hydroxylase 1
ZSWIM4	0,43052181	4,12E-13	1,12E-10	Zinc Finger SWIM-Type Containing 4
MAGT1	0,430003658	4,43E-13	1,18E-10	Magnesium Transporter 1
COL5A1	0,429691184	4,62E-13	1,22E-10	Collagen Type V Alpha 1 Chain
ANGPT2	0,429678196	4,63E-13	1,22E-10	Angiopoietin 2
COL7A1	0,42914062	4,99E-13	1,30E-10	Collagen Type VII Alpha 1 Chain
SPOCD1	0,427899242	5,91E-13	1,52E-10	SPOC Domain Containing 1
RUNDC2C	0,427071232	6,62E-13	1,67E-10	Sorting Nexin 29 Pseudogene 2
MFSD11	0,425338851	8,38E-13	2,08E-10	Major Facilitator Superfamily Domain Containing 11
LOC100133950	0,424994866	8,78E-13	2,16E-10	Hypothetical protein LOC100133950
PPEF1	0,424342501	9,60E-13	2,33E-10	Protein Phosphatase With EF-Hand Domain 1
TDRD1	0,423930625	1,01E-12	2,42E-10	Tudor Domain Containing 1
MMP11	0,423868776	1,02E-12	2,43E-10	Matrix Metallopeptidase 11
LOC728653	0,423675095	1,05E-12	2,47E-10	Uuncharacterized LOC728653
ANTXR1	0,422622234	1,21E-12	2,83E-10	ANTXR Cell Adhesion Molecule 1
SHROOM4	0,421913228	1,33E-12	3,07E-10	Shroom Family Member 4
SERPINH1	0,421241623	1,46E-12	3,33E-10	Serpin Family H Member 1
FLJ39632	0,420878977	1,53E-12	3,44E-10	Double Homeobox A Pseudogene 9
LRAP	0,420828369	1,54E-12	3,44E-10	Endoplasmic Reticulum Aminopeptidase 2
SLC38A7	0,420217815	1,67E-12	3,68E-10	Solute Carrier Family 38 Member 7
ZNF600	0,419867551	1,75E-12	3,83E-10	Zinc Finger Protein 600
COL6A3	0,419558094	1,82E-12	3,96E-10	Collagen Type VI Alpha 3 Chain
PIGX	0,419377263	1,87E-12	4,00E-10	Phosphatidylinositol Glycan Anchor Biosynthesis Class X
LOC648059	0,419120038	1,93E-12	4,09E-10	Hypothetical protein LOC648059
HIATL2	0,418131656	2,20E-12	4,51E-10	Major Facilitator Superfamily Domain Containing 14C

GLI3	0,417742293	2,32E-12	4,69E-10	GLI Family Zinc Finger 3
ZNF577	0,417487104	2,40E-12	4,81E-10	Zinc Finger Protein 577
BRD3	0,417063553	2,54E-12	5,06E-10	Bromodomain Containing 3
LPP	0,416660452	2,68E-12	5,27E-10	LIM Domain Containing Preferred Translocation Partner In Lipoma
SOX11	0,416174593	2,85E-12	5,58E-10	SRY-Box Transcription Factor 11
HNRNPU	0,4159681	2,93E-12	5,70E-10	Heterogeneous Nuclear Ribonucleoprotein U
LOC647389	0,415446212	3,14E-12	6,03E-10	Hypothetical protein LOC647389
LOC730313	0,415332368	3,19E-12	6,08E-10	Similar to postmeiotic segregation increased 2-like 2
LOC728779	0,415100641	3,29E-12	6,23E-10	Hypothetical LOC728779
MBTD1	0,414666146	3,48E-12	6,44E-10	Mbt Domain Containing 1
CTSB	0,414162084	3,72E-12	6,84E-10	Cathepsin B
KIAA1602	0,413298244	4,16E-12	7,48E-10	NCK Associated Protein 5 Like
LOC100132391	0,41312839	4,25E-12	7,55E-10	Hypothetical protein LOC100132391
ZNF148	0,413103283	4,27E-12	7,55E-10	Zinc Finger Protein 148
LOC400406	0,412307773	4,73E-12	8,32E-10	ADAMTS7 pseudogene 3
ANKRD38	0,411623393	5,17E-12	9,00E-10	KN Motif And Ankyrin Repeat Domains 4
LOC100130835	0,411615551	5,18E-12	9,00E-10	Hypothetical LOC100130835
ALPK2	0,411441305	5,30E-12	9,16E-10	Alpha Kinase 2
IGFBP3	0,410691168	5,84E-12	9,98E-10	Insulin Like Growth Factor Binding Protein 3
NPR3	0,408879614	7,37E-12	1,23E-09	Natriuretic Peptide Receptor 3
LOC100133144	0,408597926	7,64E-12	1,27E-09	Ubiquitin-conjugating enzyme E2Q family member 2 pseudogene 3
LOC100133516	0,407655461	8,63E-12	1,41E-09	Hypothetical protein LOC100133516
GPR1	0,406653332	9,80E-12	1,59E-09	G Protein-Coupled Receptor 1
COL6A1	0,406274212	1,03E-11	1,66E-09	Collagen Type VI Alpha 1 Chain
ARL16	0,40590477	1,08E-11	1,71E-09	ADP Ribosylation Factor Like GTPase 16
GDI1	0,403814621	1,41E-11	2,20E-09	GDP Dissociation Inhibitor 1
QRFPR	0,402746965	1,61E-11	2,48E-09	Pyroglutamylated RFamide Peptide Receptor
MEIS3	0,402522538	1,65E-11	2,54E-09	Meis Homeobox 3
SGIP1	0,402359766	1,69E-11	2,58E-09	SH3GL Interacting Endocytic Adaptor 1
XRCC2	0,402120899	1,74E-11	2,62E-09	X-Ray Repair Cross Complementing 2
ATXN3	0,40210111	1,74E-11	2,62E-09	Ataxin 3
DLG4	0,401860246	1,80E-11	2,68E-09	Discs Large MAGUK Scaffold Protein 4
TWIST1	0,401518382	1,88E-11	2,77E-09	Twist Family BHLH Transcription Factor 1
FZD2	0,401094332	1,98E-11	2,89E-09	Frizzled Class Receptor 2
KANK4	0,400522136	2,13E-11	3,07E-09	KN Motif And Ankyrin Repeat Domains 4
LOC440055	-0,400903124	2,03E-11	2,94E-09	Ribosomal protein S12 pseudogene 22
APIP	-0,400993198	2,00E-11	2,92E-09	APAF1 Interacting Protein
SDHA	-0,401201577	1,95E-11	2,87E-09	Succinate Dehydrogenase Complex Flavoprotein Subunit A
LOC100130707	-0,401645329	1,85E-11	2,74E-09	Hypothetical protein LOC100130707
TBL2	-0,40203217	1,76E-11	2,64E-09	Transducin Beta Like 2
LOC647081	-0,402151716	1,73E-11	2,62E-09	Succinate dehydrogenase complex subunit D pseudogene 2
MRPS18C	-0,40356406	1,45E-11	2,25E-09	Mitochondrial Ribosomal Protein S18C
NHP2	-0,403611487	1,44E-11	2,25E-09	NHP2 Ribonucleoprotein
LOC652864	-0,404983481	1,21E-11	1,91E-09	Similar to Mitochondrial import inner membrane translocase subunit Tim23
MRPS27	-0,405701168	1,11E-11	1,75E-09	Mitochondrial Ribosomal Protein S27
SFRS2B	-0,406020373	1,06E-11	1,70E-09	Serine And Arginine Rich Splicing Factor 8

MKI67IP	-0,406039073	1,06E-11	1,70E-09	Nucleolar Protein Interacting With The FHA Domain Of MKI67
C4ORF14	-0,407202565	9,14E-12	1,49E-09	Nitric Oxide Associated 1
CBWD5	-0,408011154	8,24E-12	1,36E-09	COBW Domain Containing 5
MRPL45	-0,409114589	7,15E-12	1,20E-09	Mitochondrial Ribosomal Protein L45
RPL36AL	-0,409583886	6,73E-12	1,13E-09	Ribosomal Protein L36a Like
LOC644762	-0,410144374	6,26E-12	1,06E-09	MFF pseudogene 2
IMPA2	-0,410509042	5,98E-12	1,02E-09	Inositol Monophosphatase 2
UBB	-0,411048122	5,57E-12	9,58E-10	Ubiquitin B
ATP5O	-0,413209248	4,21E-12	7,53E-10	ATP Synthase Peripheral Stalk Subunit OSCP
LOC100133372	-0,41349929	4,05E-12	7,33E-10	Similar to heterogeneous nuclear ribonucleoprotein A1
RPS5	-0,413587634	4,01E-12	7,29E-10	Ribosomal Protein S5
AHCY	-0,414099552	3,75E-12	6,86E-10	Adenosylhomocysteinase
RPS15A	-0,414710533	3,46E-12	6,44E-10	Ribosomal Protein S15a
RPL26	-0,414798721	3,42E-12	6,41E-10	Ribosomal Protein L26
ATP5J	-0,414929187	3,36E-12	6,34E-10	ATP Synthase Peripheral Stalk Subunit F6
ALG14	-0,415505111	3,12E-12	6,02E-10	ALG14 UDP-N-Acetylglucosaminyltransferase Subunit
C3ORF23	-0,416749337	2,65E-12	5,24E-10	T Cell Activation Inhibitor, Mitochondrial
HSPA8	-0,418084047	2,22E-12	4,51E-10	Heat Shock Protein Family A (Hsp70) Member 8
COX7C	-0,418657652	2,06E-12	4,23E-10	Cytochrome C Oxidase Subunit 7C
LOC728666	-0,41869406	2,05E-12	4,23E-10	PRELI domain containing 1 pseudogene 1
LOC728188	-0,418832318	2,01E-12	4,19E-10	Similar to phosphoglycerate mutase processed protein
LOC653566	-0,418963889	1,97E-12	4,14E-10	Signal peptidase complex subunit 2 pseudogene 4
IMMP2L	-0,419325259	1,88E-12	4,00E-10	Inner Mitochondrial Membrane Peptidase Subunit 2
NDUFB2	-0,419415928	1,86E-12	4,00E-10	NADH:Ubiquinone Oxidoreductase Subunit B2
LOC100129086	-0,420614697	1,58E-12	3,52E-10	HIG1 hypoxia inducible domain family member 1A pseudogene 1
LOC389386	-0,420985024	1,51E-12	3,42E-10	Leucine aminopeptidase 3 pseudogene 2
LOC644761	-0,421904687	1,33E-12	3,07E-10	Phosphatidylinositol glycan anchor biosynthesis class H pseudogene 1
RPRD1A	-0,424086236	9,93E-13	2,39E-10	Regulation Of Nuclear Pre-MRNA Domain Containing 1A
LOC399988	-0,424573111	9,30E-13	2,27E-10	Heat shock protein family A (Hsp70) member 8 pseudogene 5
LOC648390	-0,426621178	7,04E-13	1,76E-10	Similar to ubiquitin B
ATP5A1	-0,427778752	6,01E-13	1,53E-10	ATP Synthase F1 Subunit Alpha
FAM195A	-0,427866677	5,94E-13	1,52E-10	MAPK Regulated Corepressor Interacting Protein 2
MRPL20	-0,430153013	4,34E-13	1,17E-10	Mitochondrial Ribosomal Protein L20
MOCS2	-0,430793323	3,97E-13	1,09E-10	Molybdenum Cofactor Synthesis 2
LOC284821	-0,430913904	3,91E-13	1,08E-10	Ribosomal protein L13a pseudogene 7
LOC649049	-0,431018428	3,85E-13	1,08E-10	Similar to acidic ribosomal phosphoprotein P0
LOC100132717	-0,431277237	3,72E-13	1,06E-10	Hypothetical protein LOC100132717
RPL13A	-0,432133571	3,30E-13	9,47E-11	Ribosomal Protein L13a
DSCR3	-0,434100309	2,51E-13	7,56E-11	VPS26 Endosomal Protein Sorting Factor C
HINT1	-0,434868109	2,25E-13	6,98E-11	Histidine Triad Nucleotide Binding Protein 1
LOC148430	-0,434941694	2,23E-13	6,98E-11	Ribosomal protein S2 pseudogene
EIF3F	-0,436783494	1,72E-13	5,67E-11	Eukaryotic Translation Initiation Factor 3 Subunit F
LYSMD2	-0,437729902	1,51E-13	5,07E-11	LysM Domain Containing 2
CRADD	-0,438315928	1,39E-13	4,72E-11	CASP2 And RIPK1 Domain Containing Adaptor With Death Domain
RPL34	-0,438810942	1,29E-13	4,45E-11	Ribosomal Protein L34
LOC285053	-0,439503142	1,17E-13	4,07E-11	Ribosomal protein L18a pseudogene 6

MRPS33	-0,440044926	1,08E-13	3,90E-11	Mitochondrial Ribosomal Protein S33
C1QBP	-0,440167429	1,07E-13	3,88E-11	Complement C1q Binding Protein
LOC389203	-0,441124801	9,30E-14	3,55E-11	Small integral membrane protein 20
RPS13	-0,441558855	8,74E-14	3,38E-11	Ribosomal Protein S13
FAM165B	-0,441567392	8,73E-14	3,38E-11	Small Integral Membrane Protein 11A
LOC100129553	-0,442062187	8,13E-14	3,22E-11	Ribosomal protein L13a pseudogene 16
LOC441775	-0,44259501	7,53E-14	3,05E-11	Ribosomal protein L18 pseudogene 13
SLC25A3	-0,443218794	6,89E-14	2,84E-11	Solute Carrier Family 25 Member 3
UQCRFS1	-0,443959016	6,19E-14	2,62E-11	Ubiquinol-Cytochrome C Reductase, Rieske Iron-Sulfur Polypeptide 1
RPLP0	-0,444670705	5,58E-14	2,43E-11	Ribosomal Protein Lateral Stalk Subunit P0
LOC399804	-0,446224129	4,46E-14	2,05E-11	Nucleophosmin 1 pseudogene 25
LOC387867	-0,449106349	2,93E-14	1,39E-11	Ribosomal protein SA pseudogene 12
LOC648024	-0,449262442	2,86E-14	1,39E-11	Similar to eukaryotic translation initiation factor 4A, isoform 1
LOC651436	-0,451999967	1,91E-14	1,03E-11	Similar to ribosomal protein L9
LOC652624	-0,452177352	1,86E-14	1,02E-11	Ribosomal protein SA pseudogene 46
LOC641844	-0,453929676	1,43E-14	8,16E-12	Similar to Succinate dehydrogenase [ubiquinone] cytochrome b small subunit, mitochondrial precursor (CybS)
LOC286444	-0,454005275	1,42E-14	8,16E-12	Ribosomal protein S2 pseudogene 55
LOC100130562	-0,454808542	1,26E-14	7,57E-12	Ribosomal protein S2 pseudogene 5
LOC440589	-0,454933405	1,23E-14	7,57E-12	Ribosomal protein S2 pseudogene 8
SLC25A5	-0,45548581	1,14E-14	7,11E-12	Solute Carrier Family 25 Member 5
LOC100128086	-0,45606176	1,04E-14	6,75E-12	APAF1 interacting protein pseudogene
LOC729617	-0,465916537	2,32E-15	1,77E-12	Ribosomal protein L23a pseudogene 74
RPL17	-0,469235765	1,38E-15	1,11E-12	Ribosomal Protein L17
AUH	-0,469590919	1,31E-15	1,08E-12	AU RNA Binding Methylglutaconyl-CoA Hydratase
ACP1	-0,477478779	3,73E-16	3,99E-13	Acid Phosphatase 1
LOC642817	-0,483144697	1,49E-16	1,79E-13	Uncharacterized LOC642817
RPS2	-0,490932924	4,08E-17	5,32E-14	Ribosomal Protein S2
LOC347544	-0,491881917	3,47E-17	4,73E-14	Ribosomal protein L18a pseudogene 16
DPH5	-0,492221142	3,28E-17	4,68E-14	Diphthamide Biosynthesis 5
RPS4X	-0,494702789	2,15E-17	3,37E-14	Ribosomal Protein S4 X-Linked
MTMR12	-0,495791821	1,79E-17	3,11E-14	Myotubularin Related Protein 12
DCTPP1	-0,503205939	4,96E-18	1,11E-14	DCTP Pyrophosphatase 1
PRDX3	-0,508702215	1,88E-18	5,88E-15	Peroxiredoxin 3
ATP5F1	-0,525538618	8,58E-20	4,47E-16	ATP Synthase Peripheral Stalk-Membrane Subunit B
EEF1B2	-0,532906366	2,10E-20	1,32E-16	Eukaryotic Translation Elongation Factor 1 Beta 2

**TCGA**				
**Gene symbol**	**Correlation coefficient**	**p-value**	**p-value adjust**	**Gene description**
DVL3	0,604502803	3,26E-16	5,51E-12	Dishevelled Segment Polarity Protein 3
PTK7	0,591884389	1,88E-15	1,38E-11	Protein Tyrosine Kinase 7
COL11A1	0,591649536	1,94E-15	1,38E-11	Collagen Type XI Alpha 1 Chain
RUNX1	0,59124053	2,05E-15	1,38E-11	RUNX Family Transcription Factor 1
BTBD19	0,58983932	2,47E-15	1,39E-11	BTB Domain Containing 19
TRIO	0,577290794	1,29E-14	6,23E-11	Trio Rho Guanine Nucleotide Exchange Factor
FN1	0,573453432	2,11E-14	8,49E-11	Fibronectin 1

SEMA4C	0,572891124	2,27E-14	8,49E-11	Semaphorin 4C
DCBLD1	0,572083459	2,51E-14	8,49E-11	Discoidin, CUB And LCCL Domain Containing 1
PPFIBP1	0,565440911	5,77E-14	1,56E-10	PPFIA Binding Protein 1
LINC01561	0,565372857	5,82E-14	1,56E-10	Long Intergenic Non-Protein Coding RNA 1561
SGIP1	0,565112951	6,01E-14	1,56E-10	SH3GL Interacting Endocytic Adaptor 1
NUAK1	0,563283572	7,54E-14	1,82E-10	NUAK Family Kinase 1
RUNX2	0,560206124	1,10E-13	2,28E-10	RUNX Family Transcription Factor 2
ZNF532	0,560183506	1,10E-13	2,28E-10	Zinc Finger Protein 532
KIAA1217	0,559609135	1,18E-13	2,28E-10	KIAA1217
ITGA11	0,559373584	1,22E-13	2,28E-10	Integrin Subunit Alpha 11
LINC01429	0,556935296	1,63E-13	2,84E-10	Long Intergenic Non-Protein Coding RNA 1429
ADAMTS7	0,556683495	1,68E-13	2,84E-10	ADAM Metallopeptidase With Thrombospondin Type 1 Motif 7
SLC24A2	0,552886074	2,65E-13	4,26E-10	Solute Carrier Family 24 Member 2
MEX3A	0,55166318	3,06E-13	4,70E-10	Mex-3 RNA Binding Family Member A
SPOCD1	0,547652682	4,91E-13	7,15E-10	SPOC Domain Containing 1
LINC01614	0,547357655	5,08E-13	7,15E-10	Long Intergenic Non-Protein Coding RNA 1614
COL7A1	0,546867182	5,38E-13	7,23E-10	Collagen Type VII Alpha 1 Chain
KANK4	0,546298816	5,74E-13	7,23E-10	KN Motif And Ankyrin Repeat Domains 4
COL12A1	0,546242385	5,78E-13	7,23E-10	Collagen Type XII Alpha 1 Chain
CTHRC1	0,543822298	7,65E-13	9,23E-10	Collagen Triple Helix Repeat Containing 1
NCKAP5L	0,539625837	1,24E-12	1,44E-09	NCK Associated Protein 5 Like
COL10A1	0,536977216	1,67E-12	1,88E-09	Collagen Type X Alpha 1 Chain
CLTCL1	0,534634911	2,17E-12	2,37E-09	Clathrin Heavy Chain Like 1
IGFL4	0,534125959	2,30E-12	2,43E-09	IGF Like Family Member 4
PLPP4	0,533260128	2,53E-12	2,59E-09	Phospholipid Phosphatase 4
EPYC	0,532086023	2,88E-12	2,87E-09	Epiphycan
COL8A2	0,53098368	3,26E-12	3,14E-09	Collagen Type VIII Alpha 2 Chain
LRRC15	0,530193166	3,56E-12	3,34E-09	Leucine Rich Repeat Containing 15
C10orf55	0,529613462	3,79E-12	3,44E-09	Chromosome 10 Open Reading Frame 55
ADAMTS14	0,529415596	3,87E-12	3,44E-09	ADAM Metallopeptidase With Thrombospondin Type 1 Motif 14
THBS2	0,527356746	4,85E-12	4,20E-09	Thrombospondin 2
SPATA20P1	0,526511752	5,32E-12	4,49E-09	Spermatogenesis Associated 20 Pseudogene 1
POFUT2	0,525111139	6,20E-12	5,11E-09	Protein O-Fucosyltransferase 2
INTU	0,524204612	6,83E-12	5,37E-09	Inturned Planar Cell Polarity Protein
RAI14	0,524093253	6,92E-12	5,37E-09	Retinoic Acid Induced 14
YEATS2	0,5239849	7,00E-12	5,37E-09	YEATS Domain Containing 2
INPPL1	0,522447431	8,26E-12	6,20E-09	Inositol Polyphosphate Phosphatase Like 1
STAT2	0,521031672	9,61E-12	7,06E-09	Signal Transducer And Activator Of Transcription 2
CNIH3	0,520584762	1,01E-11	7,25E-09	Cornichon Family AMPA Receptor Auxiliary Protein 3
ADAMTS6	0,520342497	1,03E-11	7,28E-09	ADAM Metallopeptidase With Thrombospondin Type 1 Motif 6
STARD4-AS1	0,519270032	1,16E-11	7,86E-09	TARD4 Antisense RNA 1
GJB2	0,519242441	1,16E-11	7,86E-09	Gap Junction Protein Beta 2
ARHGEF17	0,517980344	1,33E-11	8,81E-09	Rho Guanine Nucleotide Exchange Factor 17
MICAL2	0,517384403	1,42E-11	9,20E-09	Microtubule Associated Monooxygenase, Calponin And LIM Domain Containing 2
SHANK1	0,516004388	1,64E-11	1,04E-08	SH3 And Multiple Ankyrin Repeat Domains 1
MMP14	0,514560207	1,91E-11	1,19E-08	Matrix Metallopeptidase 14

MINDY2	0,513109235	2,22E-11	1,36E-08	MINDY Lysine 48 Deubiquitinase 2
ANTXR1	0,509698981	3,16E-11	1,89E-08	ANTXR Cell Adhesion Molecule 1
MEX3B	0,509587313	3,20E-11	1,89E-08	Mex-3 RNA Binding Family Member B
NTM	0,508743922	3,49E-11	2,00E-08	Neurotrimin
CASC15	0,508736153	3,49E-11	2,00E-08	Cancer Susceptibility 15
MXRA5	0,50830795	3,65E-11	2,05E-08	Matrix Remodeling Associated 5
MXRA8	0,507669527	3,89E-11	2,16E-08	Matrix Remodeling Associated 8
COL5A1	0,507399694	4,00E-11	2,17E-08	Collagen Type V Alpha 1 Chain
KIF26B	0,50713357	4,11E-11	2,17E-08	Kinesin Family Member 26B
ATXN1	0,507090173	4,13E-11	2,17E-08	Ataxin 1
PLEKHG2	0,506975354	4,18E-11	2,17E-08	Pleckstrin Homology And RhoGEF Domain Containing G2
CMTM3	0,506606601	4,34E-11	2,22E-08	CKLF Like MARVEL Transmembrane Domain Containing 3
SLC26A10	0,506021234	4,61E-11	2,32E-08	Solute Carrier Family 26 Member 10
LOXL2	0,505630751	4,79E-11	2,38E-08	Lysyl Oxidase Like 2
FAM225B	0,505193061	5,01E-11	2,45E-08	Family With Sequence Similarity 225 Member B
SLC6A1	0,504534462	5,36E-11	2,54E-08	Solute Carrier Family 6 Member 1
FAT3	0,5045318	5,36E-11	2,54E-08	FAT Atypical Cadherin 3
GPR176	0,504416406	5,42E-11	2,54E-08	G Protein-Coupled Receptor 176
SSH1	0,504035425	5,64E-11	2,61E-08	Slingshot Protein Phosphatase 1
AEBP1	0,503428548	5,99E-11	2,74E-08	AE Binding Protein 1
NLGN2	0,502909536	6,32E-11	2,84E-08	Neuroligin 2
UBE2Q2P1	0,500403324	8,12E-11	3,61E-08	Ubiquitin Conjugating Enzyme E2 Q2 Pseudogene 1
CDH11	0,499397283	8,98E-11	3,94E-08	Cadherin 11
POSTN	0,499217451	9,14E-11	3,96E-08	Periostin
COL8A1	0,498917822	9,42E-11	4,03E-08	Collagen Type VIII Alpha 1 Chain
ADAMTS12	0,498675044	9,65E-11	4,08E-08	ADAM Metallopeptidase With Thrombospondin Type 1 Motif 12
PHF21A	0,498058484	1,03E-10	4,28E-08	PHD Finger Protein 21A
LOXL3	0,497789895	1,05E-10	4,34E-08	Lysyl Oxidase Like 3
BMP1	0,497291675	1,11E-10	4,51E-08	Bone Morphogenetic Protein 1
MRC2	0,496509636	1,20E-10	4,77E-08	Mannose Receptor C Type 2
HOXB3	0,49648139	1,20E-10	4,77E-08	Homeobox B3
NBPF9	0,495801077	1,28E-10	4,98E-08	NBPF Member 9
HSD17B6	0,495800882	1,28E-10	4,98E-08	Hydroxysteroid 17-Beta Dehydrogenase 6
NOTCH3	0,495590722	1,31E-10	5,03E-08	Notch Receptor 3
VGLL4	0,494753603	1,42E-10	5,40E-08	Vestigial Like Family Member 4
INHBA	0,49228157	1,81E-10	6,80E-08	Inhibin Subunit Beta A
ZCCHC14	0,491326974	1,99E-10	7,25E-08	Zinc Finger CCHC-Type Containing 14
IRS1	0,491304968	1,99E-10	7,25E-08	Insulin Receptor Substrate 1
RAD51AP2	0,491239191	2,00E-10	7,25E-08	RAD51 Associated Protein 2
MIR4635	0,491178883	2,02E-10	7,25E-08	MicroRNA 4635
SOX11	0,490280442	2,20E-10	7,82E-08	SRY-Box Transcription Factor 11
NKD2	0,490140102	2,23E-10	7,85E-08	NKD Inhibitor Of WNT Signaling Pathway 2
SPOCK1	0,489980165	2,26E-10	7,89E-08	SPARC (Osteonectin), Cwcv And Kazal Like Domains Proteoglycan 1
CYTH3	0,489834776	2,30E-10	7,92E-08	Cytohesin 3
MFAP2	0,488069514	2,72E-10	9,29E-08	Microfibril Associated Protein 2
ACTA2-AS1	0,486644362	3,12E-10	1,05E-07	ACTA2 Antisense RNA 1

RN7SKP296	0,486475443	3,17E-10	1,06E-07	RN7SK Pseudogene 296
ZNF469	0,486271669	3,23E-10	1,07E-07	Zinc Finger Protein 469
SULF1	0,486161923	3,27E-10	1,07E-07	Sulfatase 1
SETD5	0,485977408	3,33E-10	1,08E-07	SET Domain Containing 5
ARL4C	0,485711219	3,41E-10	1,10E-07	ADP Ribosylation Factor Like GTPase 4C
PTPN14	0,484147502	3,96E-10	1,26E-07	Protein Tyrosine Phosphatase Non-Receptor Type 14
FZD1	0,48363504	4,16E-10	1,31E-07	Frizzled Class Receptor 1
IGF1R	0,483463943	4,23E-10	1,32E-07	Insulin Like Growth Factor 1 Receptor
HOOK3	0,481748326	4,97E-10	1,54E-07	Hook Microtubule Tethering Protein 3
PRSS53	0,481332583	5,17E-10	1,59E-07	Serine Protease 53
FAP	0,480734638	5,47E-10	1,66E-07	Fibroblast Activation Protein Alpha
LINC00922	0,480328933	5,68E-10	1,71E-07	Long Intergenic Non-Protein Coding RNA 922
NKX3-2	0,48022535	5,73E-10	1,71E-07	NK3 Homeobox 2
COL1A1	0,48001739	5,85E-10	1,73E-07	Collagen Type I Alpha 1 Chain
LEF1-AS1	0,479950048	5,88E-10	1,73E-07	LEF1 Antisense RNA 1
MANBA	0,479274226	6,27E-10	1,82E-07	Mannosidase Beta
COL5A2	0,478943097	6,46E-10	1,87E-07	Collagen Type V Alpha 2 Chain
HOXB4	0,478119537	6,98E-10	1,98E-07	Homeobox B4
METTL11B	0,47810701	6,99E-10	1,98E-07	Methyltransferase Like 11B
RBMS2	0,477722326	7,24E-10	2,04E-07	RNA Binding Motif Single Stranded Interacting Protein 2
TNFSF4	0,47607893	8,43E-10	2,35E-07	TNF Superfamily Member 4
IGFL3	0,474530975	9,73E-10	2,69E-07	IGF Like Family Member 3
MAPKBP1	0,474457525	9,79E-10	2,69E-07	Mitogen-Activated Protein Kinase Binding Protein 1
UNC5B	0,473859346	1,03E-09	2,82E-07	Unc-5 Netrin Receptor B
PLXNA1	0,473537825	1,07E-09	2,86E-07	Plexin A1
ZSWIM4	0,473504978	1,07E-09	2,86E-07	Zinc Finger SWIM-Type Containing 4
MYHAS	0,473452978	1,07E-09	2,86E-07	Myosin Heavy Chain Gene Cluster Antisense RNA
SYNGAP1	0,473341054	1,09E-09	2,86E-07	Synaptic Ras GTPase Activating Protein 1
C1QTNF6	0,472944728	1,13E-09	2,95E-07	C1q And TNF Related 6
PPFIA4	0,472473922	1,17E-09	3,04E-07	PTPRF Interacting Protein Alpha 4
TIMP2	0,472412792	1,18E-09	3,04E-07	TIMP Metallopeptidase Inhibitor 2
SCUBE3	0,472278535	1,20E-09	3,04E-07	Signal Peptide, CUB Domain And EGF Like Domain Containing 3
MAML2	0,472210948	1,20E-09	3,04E-07	Mastermind Like Transcriptional Coactivator 2
FZD7	0,472189661	1,21E-09	3,04E-07	Frizzled Class Receptor 7
ADAM12	0,471885241	1,24E-09	3,10E-07	ADAM Metallopeptidase Domain 12
COL24A1	0,471622491	1,27E-09	3,15E-07	Collagen Type XXIV Alpha 1 Chain
NOX4	0,470864644	1,36E-09	3,34E-07	NADPH Oxidase 4
TANC2	0,470817937	1,37E-09	3,34E-07	NADPH Oxidase 4
RFX8	0,470675245	1,38E-09	3,36E-07	RFX Family Member 8, Lacking RFX DNA Binding Domain
KIRREL	0,470153755	1,45E-09	3,50E-07	Kirre Like Nephrin Family Adhesion Molecule 1
DOCK1	0,469820431	1,50E-09	3,58E-07	Dedicator Of Cytokinesis 1
SAMD4B	0,46955248	1,53E-09	3,65E-07	Sterile Alpha Motif Domain Containing 4B
FNDC1	0,468615392	1,67E-09	3,92E-07	Fibronectin Type III Domain Containing 1
LIMS1	0,468590757	1,67E-09	3,92E-07	LIM Zinc Finger Domain Containing 1
PACERR	0,468216608	1,73E-09	4,03E-07	PTGS2 Antisense NFKB1 Complex-Mediated Expression Regulator RNA
RASAL2	0,46810162	1,75E-09	4,04E-07	RAS Protein Activator Like 2

USP32P3	0,467820507	1,79E-09	4,12E-07	Ubiquitin Specific Peptidase 32 Pseudogene 3
DLX5	0,467752962	1,80E-09	4,12E-07	Distal-Less Homeobox 5
C1QTNF3	0,46759919	1,83E-09	4,14E-07	C1q And TNF Related 3
KLK4	0,467329486	1,87E-09	4,22E-07	Kallikrein Related Peptidase 4
COL6A3	0,467214064	1,89E-09	4,23E-07	Collagen Type VI Alpha 3 Chain
ICAM5	0,466495378	2,02E-09	4,47E-07	Intercellular Adhesion Molecule 5
PXDN	0,466467897	2,02E-09	4,47E-07	Peroxidasin
TMEM158	0,466125052	2,09E-09	4,54E-07	Transmembrane Protein 158
LINC01705	0,466090688	2,09E-09	4,54E-07	Long Intergenic Non-Protein Coding RNA 1705
TRIM59	0,466068562	2,10E-09	4,54E-07	Tripartite Motif Containing 59
PIAS3	0,466009709	2,11E-09	4,54E-07	Protein Inhibitor Of Activated STAT 3
USP35	0,465334623	2,24E-09	4,79E-07	Ubiquitin Specific Peptidase 35
MYH9	0,464467844	2,42E-09	5,14E-07	Myosin Heavy Chain 9
RNU6-403P	0,464374278	2,44E-09	5,15E-07	RNA, U6 Small Nuclear 403, Pseudogene
APBB2	0,464217577	2,47E-09	5,19E-07	Amyloid Beta Precursor Protein Binding Family B Member 2
VCAN	0,463977359	2,53E-09	5,27E-07	Versican
TENM4	0,463630788	2,61E-09	5,40E-07	Teneurin Transmembrane Protein 4
IGFL2	0,463476769	2,64E-09	5,44E-07	IGF Like Family Member 2
ST8SIA2	0,462797222	2,81E-09	5,71E-07	ST8 Alpha-N-Acetyl-Neuraminide Alpha-2,8-Sialyltransferase 2
TWIST1	0,462678125	2,84E-09	5,71E-07	Twist Family BHLH Transcription Factor 1
LATS2	0,462657648	2,84E-09	5,71E-07	Large Tumor Suppressor Kinase 2
ANOS1	0,462634731	2,85E-09	5,71E-07	Anosmin 1
BMP8A	0,462597046	2,86E-09	5,71E-07	Bone Morphogenetic Protein 8a
PIP4K2B	0,462373586	2,91E-09	5,79E-07	Phosphatidylinositol-5-Phosphate 4-Kinase Type 2 Beta
RASA2	0,462141015	2,97E-09	5,88E-07	RAS P21 Protein Activator 2
SLC12A4	0,46197346	3,02E-09	5,91E-07	Solute Carrier Family 12 Member 4
ZNF496	0,461938899	3,03E-09	5,91E-07	Zinc Finger Protein 496
AZIN2	0,461876777	3,05E-09	5,91E-07	Antizyme Inhibitor 2
COL1A2	0,461616047	3,12E-09	6,02E-07	Collagen Type I Alpha 2 Chain
CTDSP2	0,461119828	3,26E-09	6,25E-07	CTD Small Phosphatase 2
PHC1	0,460730752	3,37E-09	6,43E-07	Polyhomeotic Homolog 1
FARP1	0,460402979	3,47E-09	6,58E-07	FERM, ARH/RhoGEF And Pleckstrin Domain Protein 1
SLC25A36	0,460295663	3,50E-09	6,61E-07	Solute Carrier Family 25 Member 36
UCN2	0,460168013	3,54E-09	6,64E-07	Urocortin 2
CNN2	0,459645371	3,71E-09	6,92E-07	Calponin 2
ARAP1-AS2	0,458977909	3,93E-09	7,29E-07	ARAP1 Antisense RNA 2
RTEL1	0,458615537	4,06E-09	7,49E-07	Regulator Of Telomere Elongation Helicase 1
SYDE1	0,458476416	4,11E-09	7,54E-07	Synapse Defective Rho GTPase Homolog 1
APBA2	0,458300765	4,17E-09	7,61E-07	Amyloid Beta Precursor Protein Binding Family A Member 2
GOLGA6L5P	0,457348286	4,53E-09	8,23E-07	Golgin A6 Family Like 5, Pseudogene
CD276	0,457214105	4,58E-09	8,28E-07	CD276 Molecule
RYK	0,457050903	4,65E-09	8,35E-07	Receptor Like Tyrosine Kinase
PODNL1	0,456691371	4,80E-09	8,57E-07	Podocan Like 1
PCDH7	0,456361121	4,94E-09	8,75E-07	Protocadherin 7
SMO	0,456275221	4,97E-09	8,75E-07	Smoothened, Frizzled Class Receptor
DTX3	0,456274939	4,97E-09	8,75E-07	Deltex E3 Ubiquitin Ligase 3

MMP11	0,455838004	5,17E-09	9,04E-07	Matrix Metallopeptidase 11
HTRA1	0,454903699	5,60E-09	9,75E-07	HtrA Serine Peptidase 1
MEIS3	0,454788601	5,66E-09	9,80E-07	Meis Homeobox 3
MIAT	0,454080319	6,01E-09	1,04E-06	Myocardial Infarction Associated Transcript
PRKAB2	0,453907643	6,10E-09	1,05E-06	Protein Kinase AMP-Activated Non-Catalytic Subunit Beta 2
CRAMP1	0,453631087	6,25E-09	1,07E-06	Cramped Chromatin Regulator Homolog 1
CSMD2	0,453400296	6,37E-09	1,08E-06	CUB And Sushi Multiple Domains 2
LINC01449	0,453235029	6,47E-09	1,09E-06	Long Intergenic Non-Protein Coding RNA 1449
NUMA1	0,453145968	6,52E-09	1,10E-06	Nuclear Mitotic Apparatus Protein 1
CYP27C1	0,452959886	6,62E-09	1,11E-06	Cytochrome P450 Family 27 Subfamily C Member 1
FRMD6	0,452911109	6,65E-09	1,11E-06	FERM Domain Containing 6
LAMB1	0,452558334	6,85E-09	1,13E-06	Laminin Subunit Beta 1
GLIS2	0,45251407	6,88E-09	1,13E-06	GLIS Family Zinc Finger 2
KDM2A	0,452380129	6,96E-09	1,14E-06	Lysine Demethylase 2A
ADAM19	0,452371861	6,96E-09	1,14E-06	ADAM Metallopeptidase Domain 19
PLOD2	0,452201506	7,07E-09	1,15E-06	Procollagen-Lysine,2-Oxoglutarate 5-Dioxygenase 2
IGFBP3	0,451818775	7,30E-09	1,18E-06	Insulin Like Growth Factor Binding Protein 3
DNAH17	0,450968703	7,85E-09	1,26E-06	Dynein Axonemal Heavy Chain 17
WNT2	0,45096612	7,85E-09	1,26E-06	Wnt Family Member 2
GLIS1	0,450843153	7,93E-09	1,26E-06	GLIS Family Zinc Finger 1
MPRIP	0,45081549	7,95E-09	1,26E-06	Myosin Phosphatase Rho Interacting Protein
TEAD3	0,450759424	7,99E-09	1,26E-06	TEA Domain Transcription Factor 3
GLI3	0,450713552	8,02E-09	1,26E-06	GLI Family Zinc Finger 3
PPEF1	0,45062292	8,09E-09	1,26E-06	Protein Phosphatase With EF-Hand Domain 1
PLAU	0,450179325	8,40E-09	1,31E-06	Plasminogen Activator, Urokinase
NPR2	0,44992643	8,58E-09	1,33E-06	Natriuretic Peptide Receptor 2
C2orf27A	0,449782902	8,68E-09	1,34E-06	Chromosome 2 Open Reading Frame 27A
SERPINH1	0,449559388	8,85E-09	1,35E-06	Serpin Family H Member 1
YPEL4	0,449546402	8,86E-09	1,35E-06	Yippee Like 4
SLC6A6	0,449329302	9,03E-09	1,37E-06	Solute Carrier Family 6 Member 6
LINC01711	0,449253061	9,08E-09	1,38E-06	Long Intergenic Non-Protein Coding RNA 1711
ZNF512B	0,448938253	9,33E-09	1,41E-06	Zinc Finger Protein 512B
UAP1L1	0,448471363	9,71E-09	1,46E-06	UDP-N-Acetylglucosamine Pyrophosphorylase 1 Like 1
ZC2HC1A	0,447971452	1,01E-08	1,51E-06	Zinc Finger C2HC-Type Containing 1A
LLGL1	0,447853808	1,02E-08	1,52E-06	LLGL Scribble Cell Polarity Complex Component 1
WDR86	0,447785785	1,03E-08	1,52E-06	WD Repeat Domain 86
GPR68	0,447755757	1,03E-08	1,52E-06	G Protein-Coupled Receptor 68
EFEMP2	0,447441971	1,06E-08	1,55E-06	EGF Containing Fibulin Extracellular Matrix Protein 2
EFS	0,447416998	1,06E-08	1,55E-06	Embryonal Fyn-Associated Substrate
TIMP3	0,447148412	1,09E-08	1,58E-06	TIMP Metallopeptidase Inhibitor 3
WDR19	0,447137817	1,09E-08	1,58E-06	WD Repeat Domain 19
FNDC3B	0,44687069	1,11E-08	1,60E-06	Fibronectin Type III Domain Containing 3B
TBX19	0,446696909	1,13E-08	1,62E-06	T-Box Transcription Factor 19
CTIF	0,446529038	1,14E-08	1,63E-06	Cap Binding Complex Dependent Translation Initiation Factor
HECW1	0,446497027	1,15E-08	1,63E-06	HECT, C2 And WW Domain Containing E3 Ubiquitin Protein Ligase 1
WTIP	0,445669904	1,23E-08	1,74E-06	WT1 Interacting Protein

ADGRB2	0,445449905	1,25E-08	1,77E-06	Adhesion G Protein-Coupled Receptor B2
SKI	0,445215704	1,28E-08	1,80E-06	SKI Proto-Oncogene
KIF7	0,445072318	1,29E-08	1,80E-06	Kinesin Family Member 7
FKBP9	0,445043929	1,30E-08	1,80E-06	FKBP Prolyl Isomerase 9
RNF144A	0,445027885	1,30E-08	1,80E-06	Ring Finger Protein 144A
FAM225A	0,444600702	1,34E-08	1,86E-06	Family With Sequence Similarity 225 Member A
LEF1	0,444354365	1,37E-08	1,89E-06	Lymphoid Enhancer Binding Factor 1
TNS3	0,444059308	1,41E-08	1,92E-06	Tensin 3
ISLR	0,444054632	1,41E-08	1,92E-06	Immunoglobulin Superfamily Containing Leucine Rich Repeat
BICD2	0,444035744	1,41E-08	1,92E-06	BICD Cargo Adaptor 2
RAB31	0,443775956	1,44E-08	1,95E-06	RAB31, Member RAS Oncogene Family
PLCG1	0,443755841	1,44E-08	1,95E-06	Phospholipase C Gamma 1
DLG5	0,443516252	1,47E-08	1,98E-06	Discs Large MAGUK Scaffold Protein 5
AMZ1	0,443319431	1,50E-08	1,99E-06	Archaelysin Family Metallopeptidase 1
TIA1	0,443315548	1,50E-08	1,99E-06	TIA1 Cytotoxic Granule Associated RNA Binding Protein
ZNF281	0,443291192	1,50E-08	1,99E-06	Zinc Finger Protein 281
SCARF2	0,443232315	1,51E-08	2,00E-06	Scavenger Receptor Class F Member 2
GALNS	0,442964298	1,54E-08	2,03E-06	Galactosamine (N-Acetyl)-6-Sulfatase
NBPF15	0,442464904	1,61E-08	2,11E-06	NBPF Member 15
ASAP1	0,442185465	1,64E-08	2,15E-06	ArfGAP With SH3 Domain, Ankyrin Repeat And PH Domain 1
SH3PXD2B	0,442117062	1,65E-08	2,16E-06	SH3 And PX Domains 2B
ITGB5	0,441895032	1,68E-08	2,19E-06	Integrin Subunit Beta 5
NAB2	0,441825715	1,69E-08	2,19E-06	NGFI-A Binding Protein 2
PDGFC	0,441392565	1,75E-08	2,25E-06	Platelet Derived Growth Factor C
STK36	0,441334182	1,76E-08	2,25E-06	Serine/Threonine Kinase 36
HOXB8	0,441332462	1,76E-08	2,25E-06	Homeobox B8
EDNRA	0,441324285	1,76E-08	2,25E-06	Endothelin Receptor Type A
LOXL1	0,441303101	1,77E-08	2,25E-06	Lysyl Oxidase Like 1
HOXB-AS2	0,441059968	1,80E-08	2,28E-06	HOXB Cluster Antisense RNA 2
BCORL1	0,440959654	1,82E-08	2,29E-06	BCL6 Corepressor Like 1
PHF12	0,440847533	1,84E-08	2,31E-06	PHD Finger Protein 12
VSNL1	0,440723175	1,85E-08	2,32E-06	Visinin Like 1
FAM19A5	0,440683712	1,86E-08	2,32E-06	TAFA Chemokine Like Family Member 5
IGFL1P1	0,440596854	1,87E-08	2,33E-06	IGF Like Family Member 1 Pseudogene 1
VCAN-AS1	0,440203146	1,94E-08	2,39E-06	VCAN Antisense RNA 1
WISP1	0,440168109	1,94E-08	2,39E-06	Cellular Communication Network Factor 4
CCDC93	0,440070168	1,96E-08	2,40E-06	Coiled-Coil Domain Containing 93
PRDM6	0,439658767	2,02E-08	2,48E-06	PR/SET Domain 6
TBX5-AS1	0,438972007	2,14E-08	2,61E-06	TBX5 Antisense RNA 1
DNMT3A	0,438960228	2,14E-08	2,61E-06	DNA Methyltransferase 3 Alpha
ANKRD36	0,438782044	2,18E-08	2,63E-06	Ankyrin Repeat Domain 36
DCAF5	0,438576059	2,21E-08	2,67E-06	DDB1 And CUL4 Associated Factor 5
LINC01450	0,438189748	2,28E-08	2,75E-06	Long Intergenic Non-Protein Coding RNA 1450
KLF7	0,43777792	2,36E-08	2,83E-06	Kruppel Like Factor 7
LINC00506	0,437322938	2,45E-08	2,93E-06	Long Intergenic Non-Protein Coding RNA 506
LINC01655	0,437246462	2,47E-08	2,93E-06	Long Intergenic Non-Protein Coding RNA 1655

TMEM200A	0,436775169	2,56E-08	3,04E-06	Transmembrane Protein 200A
NBPF8	0,436198499	2,69E-08	3,17E-06	NBPF Member 8
CTSK	0,436146166	2,70E-08	3,17E-06	Cathepsin K
PKD1L2	0,436136407	2,70E-08	3,17E-06	Polycystin 1 Like 2 (Gene/Pseudogene)
ZMYM3	0,435709882	2,80E-08	3,27E-06	Zinc Finger MYM-Type Containing 3
ALPK2	0,435536368	2,84E-08	3,30E-06	Alpha Kinase 2
CLASP1	0,435127888	2,93E-08	3,40E-06	Cytoplasmic Linker Associated Protein 1
RBFOX2	0,435026816	2,96E-08	3,41E-06	RNA Binding Fox-1 Homolog 2
AMIGO2	0,435021692	2,96E-08	3,41E-06	Adhesion Molecule With Ig Like Domain 2
TMEM200B	0,434850996	3,00E-08	3,44E-06	Transmembrane Protein 200B
ANKH	0,43458978	3,06E-08	3,51E-06	ANKH Inorganic Pyrophosphate Transport Regulator
ZFHX4	0,434332982	3,13E-08	3,57E-06	Zinc Finger Homeobox 4
HIP1	0,43341943	3,37E-08	3,82E-06	Huntingtin Interacting Protein 1
MYOSLID	0,433402794	3,37E-08	3,82E-06	Myocardin-Induced Smooth Muscle LncRNA, Inducer Of Differentiation
COL3A1	0,432782022	3,54E-08	4,00E-06	Collagen Type III Alpha 1 Chain
ZNF23	0,432653911	3,58E-08	4,02E-06	Zinc Finger Protein 23
ARL10	0,432633149	3,59E-08	4,02E-06	ADP Ribosylation Factor Like GTPase 10
WDR27	0,432081497	3,75E-08	4,19E-06	WD Repeat Domain 27
PRPF40B	0,432005371	3,77E-08	4,20E-06	Pre-MRNA Processing Factor 40 Homolog B
TET1	0,431782112	3,84E-08	4,27E-06	Tet Methylcytosine Dioxygenase 1
SPECC1	0,431720124	3,86E-08	4,27E-06	Sperm Antigen With Calponin Homology And Coiled-Coil Domains 1
ISM1	0,431271152	4,00E-08	4,41E-06	Isthmin 1
ZC3HAV1L	0,430896658	4,12E-08	4,53E-06	Zinc Finger CCCH-Type Containing, Antiviral 1 Like
XXYLT1	0,430700506	4,19E-08	4,59E-06	Xyloside Xylosyltransferase 1
THBS3	0,430431786	4,28E-08	4,68E-06	Thrombospondin 3
DIRC3	0,430007545	4,42E-08	4,82E-06	Disrupted In Renal Carcinoma 3
PROSER3	0,429906459	4,46E-08	4,84E-06	Proline And Serine Rich 3
ZNF827	0,429198454	4,72E-08	5,11E-06	Zinc Finger Protein 827
RASGRF2	0,428928826	4,82E-08	5,20E-06	Ras Protein Specific Guanine Nucleotide Releasing Factor 2
ZNF362	0,428699916	4,91E-08	5,28E-06	Zinc Finger Protein 362
CMTM1	0,428613005	4,94E-08	5,30E-06	CKLF Like MARVEL Transmembrane Domain Containing 1
CHST11	0,427810161	5,27E-08	5,63E-06	Carbohydrate Sulfotransferase 11
DLGAP4	0,427725039	5,30E-08	5,65E-06	DLG Associated Protein 4
GAN	0,427640642	5,34E-08	5,67E-06	Gigaxonin
FGF11	0,427551039	5,38E-08	5,69E-06	Fibroblast Growth Factor 11
SPSB1	0,427403941	5,44E-08	5,74E-06	SplA/Ryanodine Receptor Domain And SOCS Box Containing 1
ZNF70	0,427369268	5,45E-08	5,74E-06	Zinc Finger Protein 70
ROR2	0,427138195	5,55E-08	5,82E-06	Receptor Tyrosine Kinase Like Orphan Receptor 2
LINC01096	0,427117317	5,56E-08	5,82E-06	Long Intergenic Non-Protein Coding RNA 1096
TMEM200C	0,427008688	5,61E-08	5,84E-06	Transmembrane Protein 200C
FGD1	0,427004219	5,61E-08	5,84E-06	FYVE, RhoGEF And PH Domain Containing 1
GTF3C1	0,426621473	5,79E-08	6,00E-06	General Transcription Factor IIIC Subunit 1
ST3GAL2	0,426104622	6,03E-08	6,23E-06	ST3 Beta-Galactoside Alpha-2,3-Sialyltransferase 2
KLKP1	0,426013998	6,07E-08	6,24E-06	Kallikrein Pseudogene 1
RAI1	0,425996549	6,08E-08	6,24E-06	Retinoic Acid Induced 1
BICD1	0,425833415	6,16E-08	6,30E-06	BICD Cargo Adaptor 1

DCHS1	0,425721684	6,21E-08	6,34E-06	Dachsous Cadherin-Related 1
LSM11	0,425654803	6,24E-08	6,35E-06	LSM11, U7 Small Nuclear RNA Associated
SUPT7L	0,42557697	6,28E-08	6,36E-06	SPT7 Like, STAGA Complex Gamma Subunit
OLFML2A	0,425572935	6,28E-08	6,36E-06	Olfactomedin Like 2A
S100PBP	0,425375422	6,38E-08	6,44E-06	S100P Binding Protein
KCND2	0,425064086	6,54E-08	6,58E-06	Potassium Voltage-Gated Channel Subfamily D Member 2
SLC38A7	0,424976493	6,59E-08	6,60E-06	Solute Carrier Family 38 Member 7
CEP170	0,424874521	6,64E-08	6,63E-06	Centrosomal Protein 170
CPZ	0,424805204	6,67E-08	6,65E-06	Carboxypeptidase Z
SMAD7	0,424767094	6,69E-08	6,65E-06	SMAD Family Member 7
DDX50P1	0,424330249	6,93E-08	6,86E-06	DEAD-Box Helicase 50 Pseudogene 1
CDKL5	0,424293051	6,95E-08	6,86E-06	Cyclin Dependent Kinase Like 5
PHC1P1	0,424199108	7,00E-08	6,89E-06	Polyhomeotic Homolog 1 Pseudogene 1
NPC1	0,424042802	7,09E-08	6,96E-06	NPC Intracellular Cholesterol Transporter 1
MTMR2	0,423548743	7,36E-08	7,21E-06	Myotubularin Related Protein 2
TAF6	0,423145541	7,60E-08	7,42E-06	TATA-Box Binding Protein Associated Factor 6
KCTD7	0,423005969	7,68E-08	7,47E-06	Potassium Channel Tetramerization Domain Containing 7
HOXB5	0,422993228	7,69E-08	7,47E-06	Homeobox B5
SYNC	0,422941119	7,72E-08	7,47E-06	Syncoilin, Intermediate Filament Protein
KERA	0,422005369	8,30E-08	8,02E-06	Keratocan
FAM168A	0,421762303	8,46E-08	8,15E-06	Family With Sequence Similarity 168 Member A
FLNA	0,421580342	8,58E-08	8,24E-06	Filamin A
NXN	0,420711708	9,18E-08	8,79E-06	Nucleoredoxin
RAB11FIP1P1	0,420555294	9,29E-08	8,87E-06	RAB11 Family Interacting Protein 1 Pseudogene 1
SOX4	0,419959744	9,73E-08	9,26E-06	SRY-Box Transcription Factor 4
TGFB1I1	0,419744709	9,89E-08	9,39E-06	Transforming Growth Factor Beta 1 Induced Transcript 1
SLC11A1	0,41925229	1,03E-07	9,71E-06	Solute Carrier Family 11 Member 1
COL6A1	0,419230542	1,03E-07	9,71E-06	Collagen Type VI Alpha 1 Chain
ZFYVE1	0,41895519	1,05E-07	9,89E-06	Zinc Finger FYVE-Type Containing 1
BCL9	0,418586604	1,08E-07	1,01E-05	BCL9 Transcription Coactivator
TET3	0,418544541	1,08E-07	1,01E-05	Tet Methylcytosine Dioxygenase 3
ZNF713	0,418517332	1,09E-07	1,01E-05	Zinc Finger Protein 713
BGN	0,418419889	1,10E-07	1,02E-05	Biglycan
PACS1	0,418393984	1,10E-07	1,02E-05	Phosphofurin Acidic Cluster Sorting Protein 1
NKAIN4	0,418116081	1,12E-07	1,04E-05	Sodium/Potassium Transporting ATPase Interacting 4
VCL	0,417643603	1,16E-07	1,07E-05	Vinculin
PHLDB1	0,417339269	1,19E-07	1,09E-05	Pleckstrin Homology Like Domain Family B Member 1
DIP2C	0,417309163	1,19E-07	1,09E-05	Disco Interacting Protein 2 Homolog C
NPHP3	0,417247449	1,20E-07	1,10E-05	Nephrocystin 3
ST6GAL2	0,417150297	1,21E-07	1,10E-05	ST6 Beta-Galactoside Alpha-2,6-Sialyltransferase 2
WNT5A	0,417083686	1,21E-07	1,10E-05	Wnt Family Member 5A
GGCX	0,417073185	1,21E-07	1,10E-05	Gamma-Glutamyl Carboxylase
COL6A2	0,417063563	1,22E-07	1,10E-05	Collagen Type VI Alpha 2 Chain
CD70	0,417050083	1,22E-07	1,10E-05	CD70 Molecule
CORIN	0,416981483	1,22E-07	1,10E-05	Corin, Serine Peptidase
SUSD6	0,416848189	1,24E-07	1,11E-05	Sushi Domain Containing 6

FIBIN	0,416586817	1,26E-07	1,13E-05	Fin Bud Initiation Factor Homolog
FGF1	0,416565357	1,26E-07	1,13E-05	Fibroblast Growth Factor 1
STRA6	0,416366159	1,28E-07	1,14E-05	Stimulated By Retinoic Acid 6
ACAD11	0,416250487	1,29E-07	1,15E-05	Acyl-CoA Dehydrogenase Family Member 11
SLC4A3	0,416188291	1,30E-07	1,15E-05	Solute Carrier Family 4 Member 3
MAP4K5	0,415892468	1,33E-07	1,18E-05	Mitogen-Activated Protein Kinase Kinase Kinase Kinase 5
PRKD1	0,41575972	1,34E-07	1,18E-05	Protein Kinase D1
MEGF6	0,41569179	1,35E-07	1,19E-05	Multiple EGF Like Domains 6
ANGPTL2	0,41534124	1,39E-07	1,22E-05	Angiopoietin Like 2
TRIM6	0,415146564	1,41E-07	1,23E-05	Tripartite Motif Containing 6
IGLON5	0,414941951	1,43E-07	1,25E-05	IgLON Family Member 5
SULF2	0,414748071	1,45E-07	1,26E-05	Sulfatase 2
ATP1B3-AS1	0,41425368	1,51E-07	1,31E-05	ATP1B3 Antisense RNA 1
HOXA3	0,414216162	1,51E-07	1,31E-05	Homeobox A3
SS18L1	0,413945986	1,54E-07	1,33E-05	SS18L1 Subunit Of BAF Chromatin Remodeling Complex
ANKIB1	0,413941229	1,54E-07	1,33E-05	Ankyrin Repeat And IBR Domain Containing 1
NCOR2	0,413257733	1,62E-07	1,39E-05	Nuclear Receptor Corepressor 2
ZSWIM8	0,413246792	1,62E-07	1,39E-05	Zinc Finger SWIM-Type Containing 8
XKR5	0,412397193	1,73E-07	1,48E-05	XK Related 5
NINL	0,412364121	1,74E-07	1,48E-05	Ninein Like
ODF2L	0,412331838	1,74E-07	1,48E-05	Outer Dense Fiber Of Sperm Tails 2 Like
ACTN1	0,411869838	1,80E-07	1,53E-05	Actinin Alpha 1
LIMS1-AS1	0,411725172	1,82E-07	1,54E-05	LIMS1 Antisense RNA 1
RASAL2-AS1	0,411679228	1,83E-07	1,54E-05	RASAL2 Antisense RNA 1
SRGAP2	0,411532378	1,85E-07	1,55E-05	SLIT-ROBO Rho GTPase Activating Protein 2
NETO1	0,411348845	1,87E-07	1,57E-05	Neuropilin And Tolloid Like 1
MIR1254-1	0,411251367	1,89E-07	1,57E-05	MicroRNA 1254-1
ZNF841	0,411213731	1,89E-07	1,57E-05	Zinc Finger Protein 841
SEPT8	0,411207222	1,89E-07	1,57E-05	Septin 8
EXOC7	0,411207204	1,89E-07	1,57E-05	Exocyst Complex Component 7
SYNDIG1	0,411114713	1,91E-07	1,58E-05	Synapse Differentiation Inducing 1
SNCAIP	0,410939044	1,93E-07	1,60E-05	Synuclein Alpha Interacting Protein
ADAMTS2	0,410690984	1,97E-07	1,62E-05	ADAM Metallopeptidase With Thrombospondin Type 1 Motif 2
DMTF1	0,410628636	1,98E-07	1,63E-05	Cyclin D Binding Myb Like Transcription Factor 1
UBE2D3P3	0,410081914	2,06E-07	1,69E-05	Ubiquitin Conjugating Enzyme E2 D3 Pseudogene 3
EMILIN1	0,410055328	2,06E-07	1,69E-05	Elastin Microfibril Interfacer 1
CERCAM	0,40994481	2,08E-07	1,70E-05	Cerebral Endothelial Cell Adhesion Molecule
TSPAN9	0,409807266	2,10E-07	1,71E-05	Tetraspanin 9
SALL1	0,409685101	2,12E-07	1,72E-05	Spalt Like Transcription Factor 1
KDM5B	0,409562295	2,14E-07	1,73E-05	Lysine Demethylase 5B
PLEKHH2	0,409554027	2,14E-07	1,73E-05	Pleckstrin Homology, MyTH4 And FERM Domain Containing H2
HPS3	0,408873799	2,25E-07	1,82E-05	HPS3 Biogenesis Of Lysosomal Organelles Complex 2 Subunit 1
MYOF	0,408817204	2,26E-07	1,82E-05	Myoferlin
RUSC2	0,408740961	2,28E-07	1,83E-05	RUN And SH3 Domain Containing 2
TP53BP1	0,408475851	2,32E-07	1,86E-05	Tumor Protein P53 Binding Protein 1
PXDNL	0,408420107	2,33E-07	1,86E-05	Peroxidasin Like

MMP2	0,408236384	2,36E-07	1,88E-05	Matrix Metallopeptidase 2
EDIL3	0,408225755	2,37E-07	1,88E-05	EGF Like Repeats And Discoidin Domains 3
CDC42EP3	0,408015303	2,40E-07	1,91E-05	CDC42 Effector Protein 3
FOXK1	0,407985796	2,41E-07	1,91E-05	Forkhead Box K1
CTNNB1	0,407880133	2,43E-07	1,92E-05	Catenin Beta 1
SOCS5	0,407789239	2,44E-07	1,92E-05	Suppressor Of Cytokine Signaling 5
RPSAP52	0,407654822	2,47E-07	1,94E-05	Ribosomal Protein SA Pseudogene 52
FUT11	0,407525287	2,49E-07	1,95E-05	Fucosyltransferase 11
VPS8	0,407311915	2,53E-07	1,98E-05	VPS8 Subunit Of CORVET Complex
PRRX1	0,407303071	2,53E-07	1,98E-05	Paired Related Homeobox 1
GRIP1	0,407248501	2,54E-07	1,98E-05	Glutamate Receptor Interacting Protein 1
COL5A3	0,407221389	2,55E-07	1,98E-05	Collagen Type V Alpha 3 Chain
PDGFRB	0,407089232	2,57E-07	1,99E-05	Platelet Derived Growth Factor Receptor Beta
HHIP	0,40702379	2,59E-07	2,00E-05	Hedgehog Interacting Protein
LAMA4	0,406853895	2,62E-07	2,02E-05	Laminin Subunit Alpha 4
AXL	0,406848779	2,62E-07	2,02E-05	AXL Receptor Tyrosine Kinase
FZD6	0,406820294	2,63E-07	2,02E-05	Frizzled Class Receptor 6
PLA2R1	0,406731667	2,64E-07	2,02E-05	Phospholipase A2 Receptor 1
SPIN1	0,406578873	2,67E-07	2,04E-05	Spindlin 1
ERMN	0,406551429	2,68E-07	2,04E-05	Ermin
NAV2	0,406529086	2,68E-07	2,04E-05	Neuron Navigator 2
PLEKHA8	0,406445126	2,70E-07	2,05E-05	Pleckstrin Homology Domain Containing A8
MARVELD1	0,405970575	2,80E-07	2,12E-05	MARVEL Domain Containing 1
FGD6	0,405714391	2,85E-07	2,15E-05	FYVE, RhoGEF And PH Domain Containing 6
TNFRSF19	0,405591191	2,87E-07	2,17E-05	TNF Receptor Superfamily Member 19
SLC5A3	0,405157429	2,97E-07	2,23E-05	Solute Carrier Family 5 Member 3
RBMS1P1	0,405089351	2,98E-07	2,24E-05	RNA Binding Motif Single Stranded Interacting Protein 1 Pseudogene 1
N4BP1	0,405016187	3,00E-07	2,24E-05	NEDD4 Binding Protein 1
TFAP2A	0,405000558	3,00E-07	2,24E-05	Transcription Factor AP-2 Alpha
LIMK1	0,404314437	3,16E-07	2,35E-05	LIM Domain Kinase 1
HMCN1	0,4042713	3,17E-07	2,36E-05	Hemicentin 1
MFGE8	0,404206749	3,18E-07	2,36E-05	Milk Fat Globule-EGF Factor 8 Protein
OPN3	0,404191172	3,19E-07	2,36E-05	Opsin 3
TSPY26P	0,403577428	3,33E-07	2,46E-05	Testis Specific Protein Y-Linked 26, Pseudogene
PCDHGA7	0,403222122	3,42E-07	2,52E-05	Protocadherin Gamma Subfamily A, 7
SHOX2	0,403133234	3,44E-07	2,53E-05	Short Stature Homeobox 2
SORCS2	0,402948961	3,49E-07	2,55E-05	Sortilin Related VPS10 Domain Containing Receptor 2
LDB1	0,402948303	3,49E-07	2,55E-05	LIM Domain Binding 1
XPO1	0,402671702	3,56E-07	2,60E-05	Exportin 1
LINC01050	0,402577849	3,59E-07	2,61E-05	Long Intergenic Non-Protein Coding RNA 1050
INHBA-AS1	0,402341047	3,65E-07	2,65E-05	INHBA Antisense RNA 1
LOX	0,402329397	3,65E-07	2,65E-05	Lysyl Oxidase
AXIN2	0,402263596	3,67E-07	2,65E-05	Axin 2
DLG4	0,402238402	3,68E-07	2,65E-05	Discs Large MAGUK Scaffold Protein 4
FLCN	0,402173576	3,69E-07	2,66E-05	Folliculin
ITGA5	0,402072429	3,72E-07	2,67E-05	Integrin Subunit Alpha 5

PHTF2	0,401948153	3,75E-07	2,69E-05	Putative Homeodomain Transcription Factor 2
NOD2	0,401922412	3,76E-07	2,69E-05	Nucleotide Binding Oligomerization Domain Containing 2
RPS20P33	0,401604879	3,85E-07	2,75E-05	Ribosomal Protein S20 Pseudogene 33
HOXB2	0,401350622	3,92E-07	2,79E-05	Homeobox B2
SLC39A13	0,400670584	4,12E-07	2,93E-05	Solute Carrier Family 39 Member 13
MIR1249	0,400534699	4,16E-07	2,95E-05	MicroRNA 1249
NRBP1	0,40052878	4,16E-07	2,95E-05	Nuclear Receptor Binding Protein 1
RPL23AP64	0,400371627	4,21E-07	2,97E-05	Ribosomal Protein L23a Pseudogene 64
FBXO32	0,400031147	4,31E-07	3,04E-05	F-Box Protein 32
MRPL54	-0,404213848	3,18E-07	2,36E-05	Mitochondrial Ribosomal Protein L54
ATP2A3	-0,41342115	1,60E-07	1,38E-05	ATPase Sarcoplasmic/Endoplasmic Reticulum Ca2+ Transporting 3

**GSE85916**				
**Gene symbol**	**Correlation coefficient**	**p-value**	**p-value adjust**	**Gene description**
COL8A1	0,432805778	1,54E-15	1,54E-11	Collagen Type VIII Alpha 1 Chain
KIF26B	0,419576696	1,32E-14	8,76E-11	Kinesin Family Member 26B
TNFAIP6	0,414842211	2,78E-14	1,38E-10	TNF Alpha Induced Protein 6
UNC5B	0,411158434	4,92E-14	1,65E-10	Unc-5 Netrin Receptor B
PTK7	0,411077309	4,98E-14	1,65E-10	Protein Tyrosine Kinase 7
PPEF1	0,404628888	1,33E-13	3,51E-10	Protein Phosphatase With EF-Hand Domain 1
KANK4	0,404249067	1,41E-13	3,51E-10	KN Motif And Ankyrin Repeat Domains 4
MMP14	0,401239049	2,22E-13	4,91E-10	Matrix Metallopeptidase 14
PPAPDC1A	0,397679141	3,76E-13	7,49E-10	Phospholipid Phosphatase 4
NOX4	0,396957525	4,18E-13	7,57E-10	NADPH Oxidase 4
Septin 5	0,392711033	7,77E-13	1,29E-09	Septin 5
MFAP2	0,389270156	1,28E-12	1,96E-09	Microfibril Associated Protein 2
NUAK1	0,387681903	1,60E-12	2,28E-09	NUAK Family Kinase 1
C7orf10	0,386552833	1,88E-12	2,40E-09	Succinyl-CoA:Glutarate-CoA Transferase
LOXL2	0,386365491	1,93E-12	2,40E-09	Lysyl Oxidase Like 2
FGF1	0,385345026	2,23E-12	2,62E-09	Fibroblast Growth Factor 1
PODNL1	0,384002633	2,70E-12	2,98E-09	Podocan Like 1
ADC	0,381937005	3,60E-12	3,78E-09	Antizyme Inhibitor 2
GPC1	0,37999502	4,72E-12	4,70E-09	Glypican 1
COL11A1	0,379029072	5,40E-12	4,89E-09	Collagen Type XI Alpha 1 Chain
CCNJL	0,377973115	6,25E-12	5,24E-09	Cyclin J Like
BMP1	0,375432359	8,85E-12	7,05E-09	Bone Morphogenetic Protein 1
CERCAM	0,375152878	9,20E-12	7,05E-09	Cerebral Endothelial Cell Adhesion Molecule
NKX3-2	0,374473663	1,01E-11	7,45E-09	NK3 Homeobox 2
IGFL2	0,373467803	1,16E-11	8,24E-09	IGF Like Family Member 2
FLJ22536	0,37282767	1,26E-11	8,67E-09	Cancer Susceptibility 15
FLJ39632	0,371627161	1,48E-11	9,81E-09	Double Homeobox A Pseudogene 9
MEIS3	0,371424472	1,53E-11	9,81E-09	Meis Homeobox 3
SLC12A4	0,370536852	1,72E-11	1,04E-08	Solute Carrier Family 12 Member 4
ITGA11	0,369245408	2,04E-11	1,19E-08	Integrin Subunit Alpha 11
ADAMTS12	0,369077827	2,09E-11	1,19E-08	ADAM Metallopeptidase With Thrombospondin Type 1 Motif 12
EFEMP2	0,367528384	2,57E-11	1,42E-08	EGF Containing Fibulin Extracellular Matrix Protein 2

PHLDA3	0,366691559	2,87E-11	1,55E-08	Pleckstrin Homology Like Domain Family A Member 3
GJB2	0,36538078	3,42E-11	1,79E-08	Gap Junction Protein Beta 2
ANTXR1	0,362457246	5,02E-11	2,56E-08	ANTXR Cell Adhesion Molecule 1
PXDN	0,360930226	6,13E-11	2,98E-08	Peroxidasin
ST6GAL2	0,359254021	7,61E-11	3,61E-08	ST6 Beta-Galactoside Alpha-2,6-Sialyltransferase 2
SCARF2	0,358731498	8,14E-11	3,66E-08	Scavenger Receptor Class F Member 2
C5orf46	0,358619174	8,26E-11	3,66E-08	Chromosome 5 Open Reading Frame 46
NTM	0,357933203	9,03E-11	3,91E-08	Neurotrimin
EMILIN1	0,357443378	9,61E-11	4,08E-08	Elastin Microfibril Interfacer 1
ATP10A	0,357176781	9,95E-11	4,13E-08	ATPase Phospholipid Transporting 10A
SHOX2	0,355485597	1,24E-10	4,92E-08	Short Stature Homeobox 2
APCDD1L	0,354090368	1,48E-10	5,62E-08	APC Down-Regulated 1 Like
MEX3A	0,354041248	1,48E-10	5,62E-08	Mex-3 RNA Binding Family Member A
SC65	0,35398703	1,49E-10	5,62E-08	Prolyl 3-Hydroxylase Family Member 4
LRRC15	0,351458214	2,06E-10	7,19E-08	Leucine Rich Repeat Containing 15
BMP8A	0,351065681	2,16E-10	7,43E-08	Bone Morphogenetic Protein 8a
C14orf37	0,350858797	2,22E-10	7,49E-08	Armadillo Like Helical Domain Containing 4
OLFML2A	0,350413178	2,35E-10	7,64E-08	Olfactomedin Like 2A
COL5A2	0,35039715	2,35E-10	7,64E-08	Collagen Type V Alpha 2 Chain
COL12A1	0,350306472	2,38E-10	7,64E-08	Collagen Type XII Alpha 1 Chain
LEPREL2	0,348925294	2,83E-10	8,94E-08	Prolyl 3-Hydroxylase 3
TMEM132A	0,348167811	3,11E-10	9,59E-08	Transmembrane Protein 132A
FNTB	0,348109838	3,13E-10	9,59E-08	Farnesyltransferase, CAAX Box, Beta
LAMA4	0,347988065	3,18E-10	9,59E-08	Laminin Subunit Alpha 4
ADAM12	0,346433309	3,85E-10	1,15E-07	ADAM Metallopeptidase Domain 12
RASGRF2	0,346174408	3,98E-10	1,16E-07	Ras Protein Specific Guanine Nucleotide Releasing Factor 2
ARSI	0,345952616	4,09E-10	1,18E-07	Arylsulfatase Family Member I
DLG4	0,345323578	4,42E-10	1,24E-07	Discs Large MAGUK Scaffold Protein 4
CMTM3	0,344814709	4,70E-10	1,28E-07	CKLF Like MARVEL Transmembrane Domain Containing 3
LRRC17	0,343628237	5,44E-10	1,45E-07	Leucine Rich Repeat Containing 17
GPR162	0,343618904	5,44E-10	1,45E-07	G Protein-Coupled Receptor 162
HOMER3	0,343489812	5,53E-10	1,45E-07	Homer Scaffold Protein 3
DCBLD1	0,34252292	6,22E-10	1,61E-07	Discoidin, CUB And LCCL Domain Containing 1
COL8A2	0,341615249	6,95E-10	1,75E-07	Collagen Type VIII Alpha 2 Chain
TRIO	0,34119097	7,32E-10	1,82E-07	Trio Rho Guanine Nucleotide Exchange Factor
MRC2	0,3408348	7,64E-10	1,88E-07	Mannose Receptor C Type 2
WDR86	0,340624662	7,84E-10	1,90E-07	WD Repeat Domain 86
TRO	0,340511886	7,94E-10	1,91E-07	Trophinin
COL6A2	0,340128312	8,32E-10	1,97E-07	Collagen Type VI Alpha 2 Chain
FKBP9	0,338428427	1,02E-09	2,34E-07	FKBP Prolyl Isomerase 9
SAMD14	0,338186595	1,05E-09	2,38E-07	Sterile Alpha Motif Domain Containing 14
COL5A3	0,337719255	1,11E-09	2,49E-07	Collagen Type V Alpha 3 Chain
SSH1	0,337275079	1,17E-09	2,58E-07	Slingshot Protein Phosphatase 1
SERPINH1	0,337221713	1,18E-09	2,58E-07	Serpin Family H Member 1
LEPRE1	0,336815292	1,24E-09	2,68E-07	Prolyl 3-Hydroxylase 1
RNF144A	0,336661861	1,26E-09	2,69E-07	Ring Finger Protein 144A

MMP11	0,336447448	1,29E-09	2,72E-07	Matrix Metallopeptidase 11
SOX11	0,33550045	1,45E-09	2,98E-07	SRY-Box Transcription Factor 11
SORCS2	0,333341168	1,87E-09	3,81E-07	Sortilin Related VPS10 Domain Containing Receptor 2
PDLIM7	0,330558133	2,59E-09	5,22E-07	PDZ And LIM Domain 7
CLEC11A	0,330074141	2,75E-09	5,47E-07	C-Type Lectin Domain Containing 11A
KAL1	0,329312049	3,00E-09	5,86E-07	Anosmin 1
PTPRD	0,329034154	3,10E-09	5,98E-07	Protein Tyrosine Phosphatase Receptor Type D
EFS	0,328966816	3,12E-09	5,98E-07	Embryonal Fyn-Associated Substrate
NKD2	0,328284145	3,38E-09	6,42E-07	NKD Inhibitor Of WNT Signaling Pathway 2
CHPF	0,328069975	3,47E-09	6,45E-07	Chondroitin Polymerizing Factor
COL1A1	0,327683036	3,62E-09	6,69E-07	Collagen Type I Alpha 1 Chain
SH3PXD2A	0,327530804	3,69E-09	6,74E-07	SH3 And PX Domains 2A
CTHRC1	0,326079987	4,36E-09	7,83E-07	Collagen Triple Helix Repeat Containing 1
FGF11	0,325816175	4,49E-09	7,99E-07	Fibroblast Growth Factor 11
GLIS2	0,325524615	4,65E-09	8,19E-07	GLIS Family Zinc Finger 2
SRPX2	0,325359791	4,73E-09	8,28E-07	Sushi Repeat Containing Protein X-Linked 2
INPPL1	0,325204884	4,82E-09	8,28E-07	Inositol Polyphosphate Phosphatase Like 1
RAI14	0,324512843	5,22E-09	8,88E-07	Retinoic Acid Induced 14
CCDC8	0,324238815	5,38E-09	9,09E-07	Coiled-Coil Domain Containing 8
CNIH3	0,322696662	6,42E-09	1,07E-06	Cornichon Family AMPA Receptor Auxiliary Protein 3
SYDE1	0,322077637	6,88E-09	1,14E-06	Synapse Defective Rho GTPase Homolog 1
SPHK1	0,321946051	6,99E-09	1,15E-06	Sphingosine Kinase 1
NXN	0,321805366	7,10E-09	1,16E-06	Nucleoredoxin
NOTCH3	0,320766673	7,98E-09	1,27E-06	Notch Receptor 3
HS3ST3A1	0,32063183	8,10E-09	1,28E-06	Heparan Sulfate-Glucosamine 3-Sulfotransferase 3A1
ADAMTS7	0,320588124	8,14E-09	1,28E-06	ADAM Metallopeptidase With Thrombospondin Type 1 Motif 7
ITGB5	0,319951095	8,75E-09	1,36E-06	Integrin Subunit Beta 5
ARFGAP1	0,318319128	1,05E-08	1,62E-06	ADP Ribosylation Factor GTPase Activating Protein 1
NRP2	0,317247827	1,18E-08	1,81E-06	Neuropilin 2
C1QTNF3	0,31629929	1,32E-08	1,99E-06	C1q And TNF Related 3
NLGN2	0,315411185	1,45E-08	2,15E-06	Neuroligin 2
PLXNA1	0,31539405	1,45E-08	2,15E-06	Plexin A1
TGFB1I1	0,315146358	1,49E-08	2,19E-06	Transforming Growth Factor Beta 1 Induced Transcript 1
BTBD19	0,315049387	1,51E-08	2,20E-06	BTB Domain Containing 19
PLAU	0,31494878	1,53E-08	2,20E-06	Plasminogen Activator, Urokinase
CDH13	0,313491142	1,79E-08	2,53E-06	Cadherin 13
C1QTNF6	0,313225354	1,85E-08	2,59E-06	C1q And TNF Related 6
CDH11	0,312797098	1,93E-08	2,70E-06	Cadherin 11
PPFIBP1	0,31076789	2,41E-08	3,32E-06	PPFIA Binding Protein 1
PRRX1	0,309807278	2,68E-08	3,63E-06	Paired Related Homeobox 1
MAPK8IP3	0,309733064	2,70E-08	3,63E-06	Mitogen-Activated Protein Kinase 8 Interacting Protein 3
PTPDC1	0,309275454	2,84E-08	3,74E-06	Protein Tyrosine Phosphatase Domain Containing 1
SLC39A13	0,308957671	2,94E-08	3,85E-06	Solute Carrier Family 39 Member 13
COL1A2	0,308649397	3,04E-08	3,90E-06	Collagen Type I Alpha 2 Chain
SPOCD1	0,30799625	3,26E-08	4,13E-06	SPOC Domain Containing 1
DUOXA1	0,306435363	3,85E-08	4,76E-06	Dual Oxidase Maturation Factor 1

DTX3	0,305528496	4,24E-08	5,11E-06	Deltex E3 Ubiquitin Ligase 3
FHOD3	0,305213573	4,38E-08	5,20E-06	Formin Homology 2 Domain Containing 3
FN1	0,3051991	4,39E-08	5,20E-06	Fibronectin 1
FSCN1	0,304849928	4,56E-08	5,31E-06	Fascin Actin-Bundling Protein 1
ZNF469	0,302980072	5,56E-08	6,40E-06	Zinc Finger Protein 469
GP1BB	0,302815703	5,65E-08	6,47E-06	Glycoprotein Ib Platelet Subunit Beta
FAM116B	0,302633018	5,76E-08	6,56E-06	DENN Domain Containing 6B
PRRX2	0,302325529	5,95E-08	6,64E-06	Paired Related Homeobox 2
ISLR	0,300846888	6,95E-08	7,61E-06	Immunoglobulin Superfamily Containing Leucine Rich Repeat
HEY1	0,300548747	7,17E-08	7,73E-06	Hes Related Family BHLH Transcription Factor With YRPW Motif 1
B4GALT7	0,300182683	7,45E-08	7,98E-06	Beta-1,4-Galactosyltransferase 7
ZNF33A	-0,300612867	7,12E-08	7,72E-06	Zinc Finger Protein 33A
OMA1	-0,30079303	6,99E-08	7,61E-06	OMA1 Zinc Metallopeptidase
RPS6KA3	-0,301339438	6,60E-08	7,27E-06	Ribosomal Protein S6 Kinase A3
PTGER4	-0,301797346	6,29E-08	6,97E-06	Prostaglandin E Receptor 4
ABHD14B	-0,302299369	5,97E-08	6,64E-06	Abhydrolase Domain Containing 14B
SNHG8	-0,302344062	5,94E-08	6,64E-06	Small Nucleolar RNA Host Gene 8
RPS6KA1	-0,3024153	5,90E-08	6,64E-06	Ribosomal Protein S6 Kinase A1
CD74	-0,304387976	4,79E-08	5,55E-06	CD74 Molecule
RFX5	-0,305005302	4,48E-08	5,25E-06	Regulatory Factor X5
AMD1	-0,305161937	4,41E-08	5,20E-06	Adenosylmethionine Decarboxylase 1
HNRNPA1	-0,30548938	4,26E-08	5,11E-06	Heterogeneous Nuclear Ribonucleoprotein A1
KAT2B	-0,305521261	4,24E-08	5,11E-06	Lysine Acetyltransferase 2B
CMPK1	-0,306007682	4,03E-08	4,93E-06	Cytidine/Uridine Monophosphate Kinase 1
RCSD1	-0,306356789	3,88E-08	4,77E-06	RCSD Domain Containing 1
ATP5O	-0,307162303	3,56E-08	4,43E-06	ATP Synthase Peripheral Stalk Subunit OSCP
PNRC2	-0,307680969	3,37E-08	4,22E-06	Proline Rich Nuclear Receptor Coactivator 2
CASP1	-0,307892943	3,29E-08	4,15E-06	Caspase 1
ATP5I	-0,308531634	3,07E-08	3,93E-06	ATP Synthase Membrane Subunit E
SUCLG2	-0,308775459	2,99E-08	3,87E-06	Succinate-CoA Ligase GDP-Forming Beta Subunit
AHCYL2	-0,308888991	2,96E-08	3,85E-06	Adenosylhomocysteinase Like 2
KLRB1	-0,309504761	2,77E-08	3,68E-06	Killer Cell Lectin Like Receptor B1
ALDH3A2	-0,309513868	2,76E-08	3,68E-06	Aldehyde Dehydrogenase 3 Family Member A2
PDE12	-0,310345485	2,53E-08	3,45E-06	Phosphodiesterase 12
SFPQ	-0,312610183	1,97E-08	2,73E-06	Splicing Factor Proline And Glutamine Rich
ARHGEF3	-0,314851527	1,54E-08	2,20E-06	Rho Guanine Nucleotide Exchange Factor 3
BRCC3	-0,314855005	1,54E-08	2,20E-06	BRCA1/BRCA2-Containing Complex Subunit 3
IL6ST	-0,315890481	1,38E-08	2,06E-06	Interleukin 6 Signal Transducer
APPL1	-0,317058116	1,21E-08	1,84E-06	Adaptor Protein, Phosphotyrosine Interacting With PH Domain And Leucine Zipper 1
GSPT1	-0,320852309	7,90E-09	1,27E-06	G1 To S Phase Transition 1
ANAPC16	-0,320871327	7,89E-09	1,27E-06	Anaphase Promoting Complex Subunit 16
RPS28	-0,325254533	4,79E-09	8,28E-07	Ribosomal Protein S28
ACADM	-0,3263636	4,22E-09	7,64E-07	Acyl-CoA Dehydrogenase Medium Chain
GIMAP4	-0,328137878	3,44E-09	6,45E-07	GTPase, IMAP Family Member 4
SOD1	-0,32956924	2,91E-09	5,74E-07	Superoxide Dismutase 1
C19orf43	-0,335916253	1,38E-09	2,86E-07	Telomerase RNA Component Interacting RNase

RPL11	-0,336626181	1,27E-09	2,69E-07	Ribosomal Protein L11
OXNAD1	-0,338413064	1,02E-09	2,34E-07	Oxidoreductase NAD Binding Domain Containing 1
MID1IP1	-0,339678657	8,79E-10	2,06E-07	MID1 Interacting Protein 1
ATPAF1	-0,342164976	6,50E-10	1,66E-07	ATP Synthase Mitochondrial F1 Complex Assembly Factor 1
C6	-0,345075903	4,55E-10	1,26E-07	Complement C6
HIPK2	-0,345301718	4,43E-10	1,24E-07	Homeodomain Interacting Protein Kinase 2
EPB41	-0,351513652	2,04E-10	7,19E-08	Erythrocyte Membrane Protein Band 4.1
LYN	-0,352511516	1,80E-10	6,53E-08	LYN Proto-Oncogene, Src Family Tyrosine Kinase
MAOA	-0,353664177	1,56E-10	5,75E-08	Monoamine Oxidase A
HSDL2	-0,355551641	1,22E-10	4,92E-08	Hydroxysteroid Dehydrogenase Like 2
GSTK1	-0,35861191	8,27E-11	3,66E-08	Glutathione S-Transferase Kappa 1
MBNL3	-0,362224591	5,17E-11	2,58E-08	Muscleblind Like Splicing Regulator 3
RPL14	-0,371086121	1,60E-11	9,94E-09	Ribosomal Protein L14
IQGAP2	-0,377897675	6,31E-12	5,24E-09	IQ Motif Containing GTPase Activating Protein 2
C3orf23	-0,379039121	5,39E-12	4,89E-09	T Cell Activation Inhibitor, Mitochondrial

Table S7. Univariate analyses of different pathways for overall survival in the ICGC cohort.

	**Univariate analysis**			
**Pathways**	**No. of patients**	**No. of events**	**Hazard ratio (95%CI)**	**P-value***
**Stemness**	257	153	0.96 (0.26 – 3.53)	0.96
**Metabolic**	257	153	0.96 (0.26 – 3.53)	0.96
**Fibroblasts**	257	153	1.03 (0.85 – 1.24)	0.78
**F-TBRS**	257	153	1.439 (0.77 – 2.67)	0.25
**TGFB CAF**	257	153	1.03 (0.88 – 1.21)	0.72

*Cox-proportional-hazard models used to estimate the association of the parameters with overall survival. Values of P<0.05 were considered statistically significant and all tests were two-sided.
